# Prediction and analysis of tumor infiltrating lymphocytes across 28 cancers by TILScout using deep learning

**DOI:** 10.1038/s41698-025-00866-0

**Published:** 2025-03-19

**Authors:** Huibo Zhang, Lulu Chen, Lan Li, Yang Liu, Barnali Das, Shuang Zhai, Juan Tan, Yan Jiang, Simona Turco, Yi Yao, Dmitrij Frishman

**Affiliations:** 1 Department of Bioinformatics, TUM School of Life Sciences, Technical University of Munich, Freising, Germany; 2 Cancer Center, Renmin Hospital of Wuhan University, Wuhan, China; 3 Department of Pathology, The Third Xiangya Hospital, Central South University, Changsha, China; 4 Electrical Engineering, Eindhoven University of Technology, Den Dolech 12, Eindhoven, 5612AZ the Netherlands

**Keywords:** Computational biology and bioinformatics, Cancer microenvironment, Tumour immunology

## Abstract

The density of tumor-infiltrating lymphocytes (TILs) serves as a valuable indicator for predicting anti-tumor responses, but its broad impact across various types of cancers remains underexplored. We introduce TILScout, a pan-cancer deep-learning approach to compute patch-level TIL scores from whole slide images (WSIs). TILScout achieved accuracies of 0.9787 and 0.9628, and AUCs of 0.9988 and 0.9934 in classifying WSI patches into three categories—TIL-positive, TIL-negative, and other/necrotic—on validation and independent test sets, respectively, surpassing previous studies. The biological significance of TILScout-derived TIL scores across 28 cancers was validated through comprehensive functional and correlational analyses. A consistent decrease in TIL scores with an increase in cancer stage provides direct evidence that the lower TIL content may stimulate cancer progression. Additionally, TIL scores correlated with immune checkpoint gene expression and genomic variation in common cancer driver genes. Our comprehensive pan-cancer survey highlights the critical prognostic significance of TILs within the tumor microenvironment.

## Introduction

Growing evidence suggests that the tumor microenvironment significantly influences cancer development, progression, therapeutic response, outcomes, and resistance through complex interactions of its components^1–3^. Tumor-infiltrating lymphocytes (TILs), a vital population of immune cells in the tumor microenvironment, play a crucial role in effective anti-tumor immune responses. High densities of TILs have been associated with improved outcomes of immune checkpoint blockade (ICB) therapy and prognosis in breast cancer^4–7^, melanoma^8^, colon cancer^9^, non-small cell lung cancer^10–12^, gastric cancer^13^, and laryngeal squamous cell carcinoma^14^. Distinct spatial patterns of TILs have been linked to survival in melanoma^15,16^. However, for tumors with low immunogenicity, such as adenoid cystic carcinoma, TIL patterns do not seem to affect prognosis, even in the presence of abundant lymphocytes^17^, suggesting that effects of TILs on prognosis and therapy response vary depending on the molecular mechanisms underlying cancer progression. A comprehensive and systematic investigation of the relationships between TILs and therapeutic response and prognosis across diverse cancers has so far been lacking.

As the gold standard in cancer diagnosis, histopathology slides provide a wealth of information about tumor tissue architecture, encompassing both tumor cells and the surrounding microenvironment. Due to the subjective nature and lack of reproducibility in manual assessments of histopathological images, computational analyses have gained prominence in recent years^18,19^. Numerous studies have explored prognostic features from whole slide images (WSIs) using deep learning models across various cancer types^20–26^. As the most critical component of anti-tumor response in the tumor microenvironment, TILs exhibit distinctive morphological characteristics, facilitating their computational identification in pathological images. Typically appearing as small, round cells with high nuclear-to-cytoplasmic ratios and darkly stained nuclei, TILs are easily distinguishable from tumor cells, which generally feature enlarged nuclei, irregular cell shapes, and increased nucleocytoplasmic ratios. The large size of WSIs makes it challenging for pathologists to evaluate TIL proportions throughout the entire tissue slides, which is laborious and time-consuming. Therefore, machine learning-aided approaches emerge as an efficient tool for WSI analysis.

In the past five years, computer-based methodologies have made notable advancements in the detection of TILs from hematoxylin-eosin slides, particularly in the context of individual cancer types such as breast cancer^6,27–30^ and lung cancer^11,12,31^. These approaches have primarily focused on cellular-level identification and evaluation of TILs. Despite variations in methodology and some deviation from the visual TIL assessment (VTA) guidelines of The International Immuno-Oncology Biomarker Working Group on Breast Cancer (TILs-WG)^32^, the outcomes of these studies have consistently demonstrated excellent concordance with VTA and statistical association with the prognosis. Notably, applications of many of these algorithms relied on initial manual annotation of tumor regions by pathologists. Moreover, there is a shortage of evidence regarding the applicability of these approaches to slide images of other cancer types, while the prediction accuracy remains below 90%^29,33–36^.

The patch-based approach is one of three main computational approaches for quantifying tissues and cells in slide images^37^. A pivotal study by Saltz et al.^38^ presented patch-level mapping of TILs based on slide images in 13 cancer types. Their methodology incorporated a semi-supervised convolutional neural network (CNN) for patch-level classification of infiltrated lymphocytes and necrosis segmentation. However, the approach of Saltz et al. requires integrating multiple separate deep learning-based methods and manual analytical steps, which complicates its use in clinical applications. Additionally, the model initially published by Saltz et al. achieved a patch-level prediction accuracy of 0.7956, and the best accuracy of their updated deep-learning workflow reached 0.8743 across 23 cancer types^39^. The DeepTILs approach developed by Xu et al.^40^ for patch-level TILs prediction showed a test accuracy value of 0.8006 in colorectal cancer. Another study by Le et al.^29^ developed two types of convolutional neural network models for detecting breast cancer regions and TILs at the patch level, while the optimal accuracy for TIL patch classification was 0.89. A more accurate, easy-to-use, and pan-cancer-applicable model for TIL detection and assessment in WSIs would thus be essential in the context of immunotherapy.

Building upon the experience of the previous studies, we developed a pan-cancer applicable deep learning-based approach named TILScout for fully automatic TIL assessment at the patch level from WSIs. The reliability and practicality of TILScout-generated TIL scores in evaluating the extent of TIL infiltration across various cancers were validated through comprehensive analyses encompassing both qualification and quantification. We also investigated the influence of genomic variation on TIL infiltration. Furthermore, prognostic models integrating clinical characteristics were established to comprehensively explore the prediction performance of TIL scores for survival and therapeutic response and elucidate potential application in clinical practice.

## Results

### Overview of the study

**Fig. 1:**
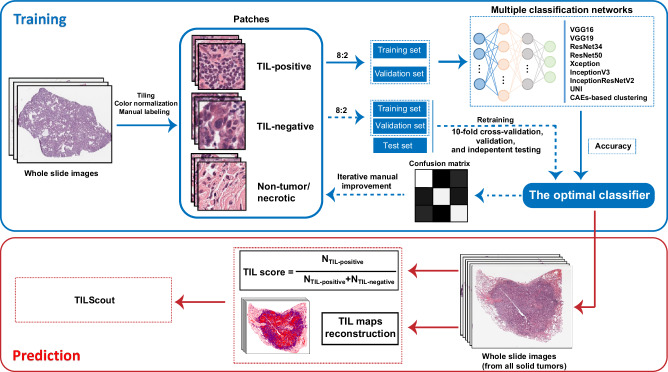
Workflow of model training and TIL score prediction.

The goal of this study was to develop a computational method to predict the amount of tumor infiltration lymphocytes (TIL) present in tumor tissues based on whole slide images (WSI) (Fig. 1). WSIs were split into thousands of patches, which were manually labeled as TIL-positive, TIL-negative, and non-tumor/necrotic by experienced pathologists. This dataset was used to train and test nine machine-learning models, with InceptionResNetV2 emerging as the optimal classifier for predicting patch labels. Iterative manual improvement and relabeling of the patch dataset was performed according to the confusion matrix of the InceptionResNetV2 classifier, after which it was retrained and the best model resulting from the ten-fold cross-validation was chosen as the final model for patch prediction. We provide our approach called TILScout to compute TIL scores (the fraction of TIL-positive patches in tumor regions of a given WSI) and to construct TIL maps illustrating the extent of TIL infiltration within tumor tissue.

### Selection of an optimal patch classifier

Eight pre-trained convolutional neural networks (VGG16, VGG19, ResNet34, ResNet50, Xception, InceptionV3, InceptionResNetV2, UNI), CAE-based clustering were utilized to discriminate between TIL-positive, TIL-negative, and non-tumor/necrotic patches in the initial dataset of 90,488 patches. InceptionResNetV2 showed the best performance on the validation set with the highest accuracy (0.9838), Kappa value (0.9753), AUC (0.9989), precision (0.9838), recall (0.9838), specificity (0.9909), and F1 score (0.9838) (Supplementary Table 10) and the lowest loss value (Supplementary Fig. 2). On the other hand, unsupervised learning methods CAE-based deep clustering yielded the least favorable results, with the lowest accuracy value (0.5096). Consequently, InceptionResNetV2 was chosen for retraining the final classifier.

### Manual improvement and re-labeling of the patch dataset

As described in “Methods”, a team of experienced pathologists at the Third Xiangya Hospital of Central South University conducted manual improvement of patches for which the prediction results did not match the actual labels based on the confusion matrix. These potentially erroneously labeled patches were reviewed and relabeled. After one iteration of manual improvement, the final dataset of 90,488 patches was produced.

### Model retraining to obtain the final classifier for patch prediction

**Fig. 2:**
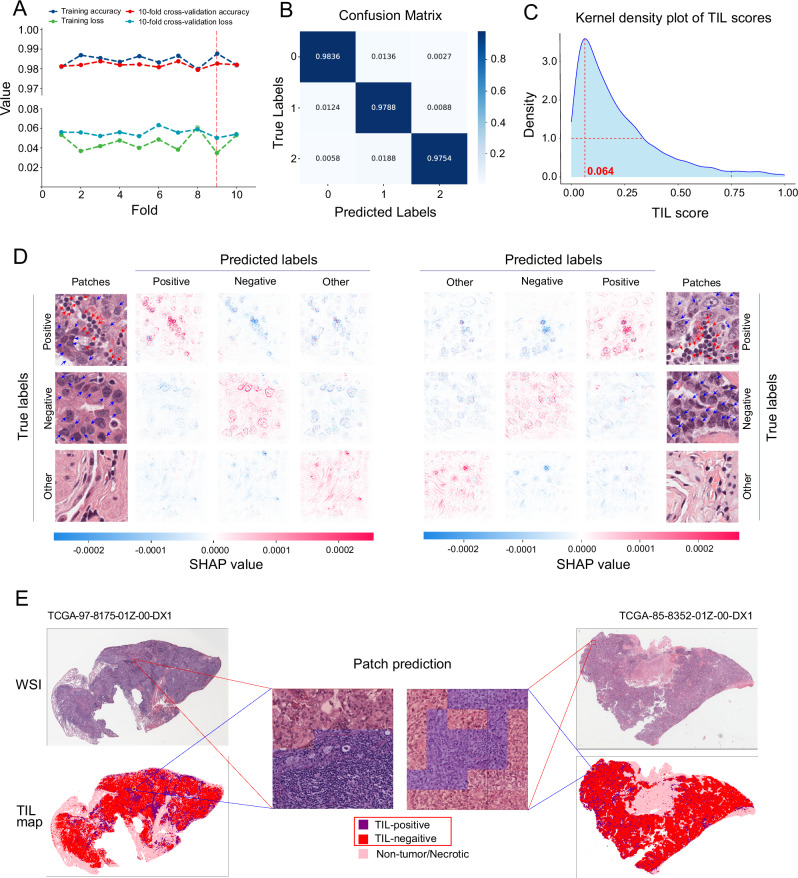
Performance of the InceptionResNetV2 model and examples of patch prediction and patch-level TIL map construction. **A** InceptionResNetV2 model performance using 10-fold cross-validation. The fold-9 model was selected for optimal performance (with the highest accuracy and lowest loss value). **B** Confusion matrix of the fold-9 InceptionResNetV2 model on the validation set. 0, TIL-positive patches; 1, TIL-negative patches; 2, non-tumor/necrotic patches. **C** kernel density estimation of TIL scores across all samples. The red dotted line marks the TIL scores corresponding to the highest density. **D** Examples of explaining individual predictions by SHAP visualization. SHAP values for each imputed feature (RGB values of each pixel) were computed and visualized according to the trained model. Positive values indicate a positive contribution to the prediction, while negative values indicate a negative contribution. **E** Two examples of WSIs, patch prediction and patch-level TIL map construction. Different colors mark different patch labels according to prediction results. A patch-level TIL map was constructed according to the positions of each patch within the WSI.

The final dataset of patches was randomly split into a training (72,272 patches) and a validation (18,216 patches) set. We retrained the InceptionResNetV2-based model using 10-fold cross-validation, which achieved an average accuracy of 0.9842 on the training set and 0.9820 on the cross-validation set (Fig. 2A). The best results were obtained on fold 9), with excellent accuracy (0.9787), Kappa (0.9669), AUC (0.9988), precision (0.9788), recall (0.9787), specificity (0.9705), and F1 score (0.9787) on the validation set (Supplementary Table 10, *V*_imi_). According to the confusion matrix (Fig. 2B) in the validation set, the model is able to discriminate between distinct label categories. Furthermore, we conducted a separate analysis on a subset of 5512 patches in the validation set that originated from cases different from those in the training set. The results indicate that the model achieved an accuracy of 0.9777 and an AUC of 0.9991 (Supplementary Table 10, *V*_isub_), demonstrating its ability to generalize effectively even when dealing with patches from entirely new slides. The model achieved an average accuracy of 0.9628 (RUMC-BRCA: 0.9643, CPTAC-LUAD: 0.9613, CPTAC-LUSC: 0.9627) and an average AUC of 0.9934 (RUMC-BRCA: 0.9925, CPTAC-LUAD: 0.9917, CPTAC-LUSC: 0.9960) on the independent test set (Supplementary Table 10). The best model from fold 9 was selected as the final TILScout predictor and subsequently applied for patch prediction and TIL score calculation. This model was compared with the previously published models (Supplementary Table 11).

### TIL score computation by TILScout and construction of patch-level TIL maps for WSIs

Using the final trained model, we conducted large-scale classification of TIL patches and calculated TIL scores in 8,888 WSIs derived from 7,699 samples across 28 cancer types (ACC, BLCA, BRCA, CESC, CHOL, COAD-READ, ESCA, HNSC, KICH, KIRC, KIRP, LIHC, LUAD, LUSC, MESO, OV, PAAD, PCPG, PRAD, SARC, SKCM, STAD, TGCT, THCA, THYM, UCEC, UCS, UVM).

According to the kernel density estimation distribution presented in Fig. 2C, TIL scores are concentrated between 0.000 and 0.339 (densities greater than 1.0), accounting for 79.4% of all values. The TIL score corresponding to the highest density value was around 0.064. Only a few WSIs exhibited TIL scores greater than 0.750 (3.3%).

**Fig. 3:**
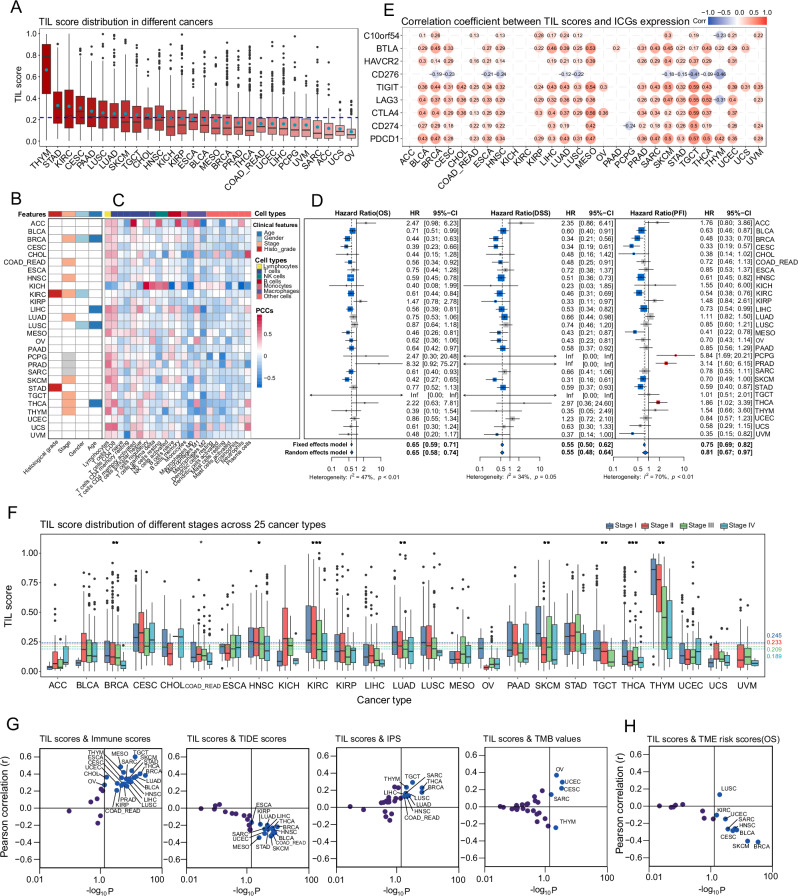
Pan-cancer analysis of TIL scores. **A** TIL score distribution across 28 cancer types. Blue dashed line: the average TIL score across all cancer types; blue dots: mean values of TIL scores for each cancer type. **B** Correlations between TIL score and clinical features. Color-filled squares indicate a significant relationship between TIL scores and features (Kruskal–Wallis test, *P* < 0.05). Grey squares indicate that no information about these features was provided for the corresponding cancer types. **C** Correlations between TIL scores and immune cell fractions. PCCs Pearson correlation coefficients. Color-filled squares indicate significant relationships (*P* < 0.05). **D** Forest plots of survival analysis. HR values below and greater that 1 indicate that TIL scores are associated with a decrease and increase in the risk of death (OS and DSS) or recurrence (PFI), respectively. 95% CI 95% confidence interval (CI) of HR. Blue squares indicate significant effects of TIL scores on outcomes (OS, DSS, PFI) (the upper 95% CI less than 1). *I*^2^ inter-group heterogeneity test index, assessing the degree of variability among studies (different cancer types in our study) that was attributable to heterogeneity rather than to chance^84,85^. A value of *P* > 0.1 indicates a lack of heterogeneity among effects (different cancer types). A fixed-effects model was used if the *P*-value of *I*^2^ was greater than 0.1, otherwise, a random-effects model was considered^86^. The size of the square represents the weight or contribution of each cancer type to the overall effect estimate. **E** Pearson correlations between TIL score and expressions of immune checkpoint genes (ICGs). Color-filled circles indicate a significant relationship (*P* < 0.05). **F** TIL score distribution in different stages across 25 cancer types. A *P* < 0.05 indicates that TIL score distribution has a significant difference. “*”, *P* < 0.05; “**”, *P* < 0.01; “***”, *P* < 0.001. **G** Correlations between TIL scores and Immune scores, TIDE scores, IPS and TMB values. The vertical solid line is the dividing line where the *P*-value equals 0.05. Cancer types with *P* < 0.05 are labeled. **H** Correlations between TIL scores and OS-based TME risk scores. The vertical solid line is the dividing line where the *P*-value equals 0.05. Cancer types with *P* < 0.05 are labeled.

In Fig. 2D, we present two examples explaining individual predictions by the SHapley Additive exPlanations (SHAP)^41^. Shapley values reflect the importance of imputed features, where each imputed feature for every patch corresponds to the RGB value of each pixel. Positive SHAP values signify a positive contribution to the prediction, while negative values indicate a negative contribution. In accordance with our defined criteria (Method, 2.2.2), patches containing more than one tumor cell (blue arrows) and three lymphocytes (indicated by red arrows) would be predicted as TIL-positive. Conversely, patches with more than one tumor cell and fewer than three lymphocytes would be predicted as TIL-negative, while patches devoid of any tumor cells would be predicted as other/necrotic. The predictive model successfully identifies TIL-positive patches based on the criteria specified in “Methods”. Figure 3D illustrates the predicted labels and their correspondence to the actual labels.

Additionally, we present two instances of patch-level TIL map construction (TCGA id: TCGA-66-2794-01Z-00-DX1, TCGA-85-8532-01Z-00-DX1) (Fig. 2E). Different colors were employed to delineate distinct predicted labels of patches in accordance with the prediction results. The construction of the patch-level TIL map involved positioning each patch within its corresponding WSI.

### Association of TIL scores with clinical characteristics of patients

Across multiple cancers, the overall mean TIL score was 0.219, with thymoma (THYM) and ovarian cancer (OV) exhibiting the highest (0.664) and the lowest (0.088) average TIL scores, respectively (Fig. 3A). We assessed correlations between TIL scores and clinical and pathological characteristics, including age, gender, stage, and histological grade, across 28 cancer types (Fig. 3B). Notably, a significant association between TIL scores and cancer stages were identified in 9 out of 25 (36.0%) cancer types. A consistent decreasing trend in TIL scores with the increase in cancer stage was observed (Fig. 3F) (average TIL scores across all cancer types: stage I, 0.245; stage II, 0.233; stage III, 0.209; stage IV, 0.189).

### TIL scores reflect the extent of TILs infiltration in tumor microenvironments

TIL scores exhibit a significant positive correlation with lymphocyte fractions (estimated by the CIBERSORT algorithm) in 23 out of 28 cancer types, primarily due to the contribution of CD8 + T cells (Fig. 3C). Notably, an inverse correlation was observed in KICH, which is a specific type of renal cell carcinoma (RCC) with significantly decreased immune cell gene-specific signatures compared to other RCC types^42,43^. However, TIL scores exhibited a notably positive correlation with regulatory T cells in KICH.

### TIL scores as predictive pan-cancer biomarkers

The high TIL score group exhibited reduced risks of death (OS benefits) in 22 out of 28 cancer types, reduced risk of cancer-specific death (DSS benefits) in 22 out of 28 cancer types and reduced risk of recurrence (PFI benefits) in 20 out of 28 cancer types (HR < 1) (Fig. 3D). High TIL score groups demonstrated significant overall survival (OS) benefits in 12 out of 28 cancer types (BLCA, BRCA, CESC, COADREAD, HNSC, KIRC, LIHC, MESO, OV, PAAD, SARC, SKCM) with a 29–61% decrease in the risk of death (HR 0.39–0.71). Disease-specific survival (DSS) benefits were evident in 15 out of 28 cancer types (BLCA, BRCA, CESC, HNSC, KIRC, KIRP, LIHC, LUAD, MESO, OV, PAAD, SKCM, STAD, UVM), presenting a 34–69% decrease in the risk of death (HR 0.31–0.66). Progression-free interval (PFI) benefits were observed in 11 out of 28 cancer types (BLCA, BRCA, CESC, CHOL, HNSC, KIRC, LIHC, MESO, SKCM, STAD, UVM) with a 27–67% decrease in the risk of recurrence (HR 0.33–0.73). However, the low TIL score group in three cancer types (PCPG, PRAD, THCA) showed significant PFI benefits, possibly due to the heterogeneity of different cancers. Overall, the high TIL scores group exhibited a 35% decrease in the risk of death (OS, HR 0.65, 95 CI [0.58, 0.74], random-effects model), a 45% decrease in the risk of cancer-specific death (DSS, HR 0.55, 95 CI [0.50, 0.62], fixed-effects model), and a 19% decrease in the risk of cancer recurrence (PFI, HR 0.81, 95 CI [0.67, 0.97], random-effects model) (Fig. 3D) across all cancer types.

### TIL scores exhibit strong positive correlations with the expression of immune checkpoint genes

We investigated the associations between TIL scores and the expression levels of nine common ICGs (Fig. 3E). In addition to the identified negative correlation between the expression of CD276 and TIL scores, the remaining 8 ICGs (PDCD1, CD274, CTLA4, LAG3, TIGIT, HAVCR2, BTLA, C10orf54) displayed significant positive correlations with TIL scores across most cancer types (Pearson *P* < 0.05), with the highest correlation observed in TGCT. Notably, expression levels of two pivotal ICGs, PDCD1 and CTLA4, which are mainly expressed on lymphocytes^44^, exhibited correlations with TIL scores in 20 out of 28 cancer types. Overall, TIL scores showed a statistically significant correlation with ICG expression in most cancers, which was substantially higher than the same values for randomly chosen sets of 9 human genes (Supplementary Fig. 5). Moreover, TIL scores exhibited a positive correlation with immune scores in 21 out of 28 cancer types and IPS in 10 out of 28 cancer types, while showing a negative correlation with TIDE scores in 14 out of 28 cancer types (Fig. 3G). These findings suggest that tumor microenvironments of patients with high TIL scores are characterized by elevated ICGs expression in most cancer types, which might contribute to potential response to immunotherapy. Regarding TMB, correlations with TIL scores were observed in only five cancer types (CESC, THYM, UCEC, OV, SARC) (Fig. 3G).

### TIL scores are significantly associated with TME risk scores across most cancer types

We developed three separate TME risk scoring systems for predicting the OS in 17 cancer types (Supplementary Table 5), DSS in 17 cancer types (Supplementary Table 7), and PFI in 18 cancer types (Supplementary Table 9). Notably, the TME risk scoring systems exhibited robust prognostic predictive capabilities. The high TME risk score group demonstrated a 2.61 times higher risk of death (OS, HR 2.61, 95% CI [2.39, 2.86], fixed-effects model) (Supplementary Fig. 6), a 3.37 times higher risk of cancer-specific death (DSS, HR 3.37, 95% CI [2.70, 4.19], random-effects model) (Supplementary Fig. 7), and a 3 times higher risk of cancer recurrence (PFI, HR 3.00, 95% CI [2.51, 3.59], random-effects model) (Supplementary Fig. 8). More importantly, TIL scores revealed statistically significant correlations with OS-based TME risk scores in 9 out of 17 cancer types (BLCA, BRCA, CESC, HNSC, KIRC, LUSC, SARC, SKCM, UCEC) (Fig. 3H), with particularly strong correlations observed in BRCA and SKCM. TIL scores also showed statistically significant correlations with DSS-based TME risk scores in 9 out of 17 cancer types (BLCA, BRCA, CESC, HNSC, KIRC, KIRP, SARC, SKCM, UCEC) (Supplementary Fig. 9A), and with PFI-based TME risk scores in 8 out of 18 cancer types (BLCA, BRCA, CESC, HNSC, KIRC, PRAD, SKCM, UCEC) (Supplementary Fig. 9B). These findings provide robust evidence supporting the potential relevance of TIL scores on prognosis across different cancer types.

### Elevated TIL scores reflect upregulated immune activity in the tumor microenvironments

**Fig. 4:**
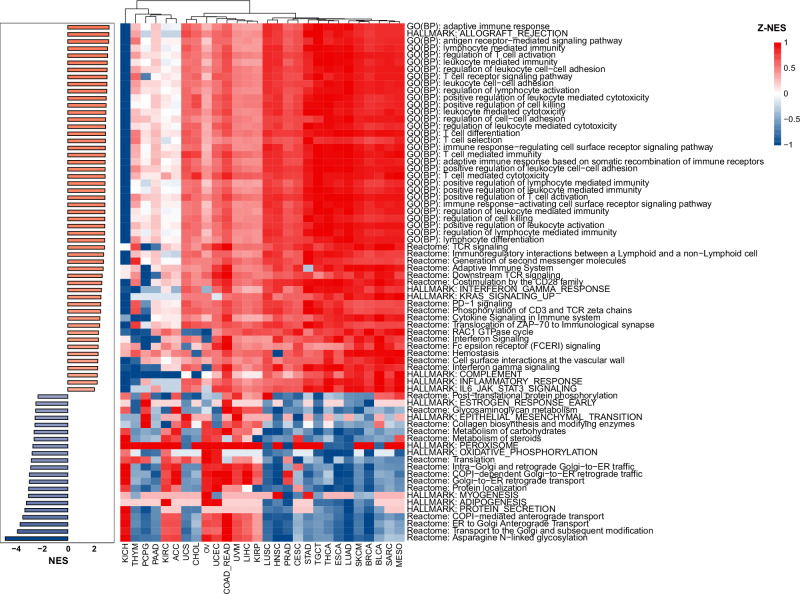
Gene Set Enrichment Analysis. NES normalized enrichment score. Z-NES, Z-score normalized NES.

Gene set enrichment analysis was conducted for three types of gene annotations (GO, Reactome, and Hallmark). We identified 432 significantly enriched biological functional gene sets/pathways (354 GO terms, 13 hallmark gene sets, and 65 Reactome pathways) correlated with TIL scores (Supplementary Data 3). The top-ranked gene sets/pathways of each type (30 GO terms, 13 hallmark gene sets, and 30 Reactome pathways) are presented in Fig. 4. To ensure comparability across different cancer types for selected terms or pathways, we normalized the NES of each term/pathway by Z-score. All 52 upregulated gene sets/pathways (NES > 0) were associated with the immune system. These gene sets/pathways were identified as upregulated in more than 75% of cancer types, encompassing the entire process of the adaptive immune response, including antigen processing and presentation, immune cell activation, and immune killing. For example, GO terms “regulation of lymphocyte activation”, “leukocyte cell−cell adhesion” and “ T cell differentiation” were enriched in 27 out of 28 cancer types. Reactome pathways such as “PD-1 signaling” and “co-stimulation by the CD28 family” were highly upregulated in 26 out of 28 cancer types. The Hallmark gene set “HALLMARK_ALLOGRAFT_REJECTION,” a direct reflection of immune system activation, exhibited the highest positive NES score and was upregulated in 24 out of 28 cancer types. At the same time, some cancer types, especially KICH, exhibited significant downregulation of these gene sets/pathways. This phenomenon might be attributed to the specificity and heterogeneity of KICH itself, which has been discussed before^42,43^. The 21 gene sets/pathways (NES < 0), which were predominantly involved in the regulation of biological processes in tumor cells (metabolic activity, protein synthesis and secretion, cell proliferation, invasive and migratory), were found to be downregulated in most cancer types, but with significant heterogeneity observed across different cancer types. These results underscore that the TIL score can be considered a valuable biomarker for the immune activity in the tumor microenvironment.

### The impact of genome variation on the extent of TME infiltration by TILs

**Fig. 5:**
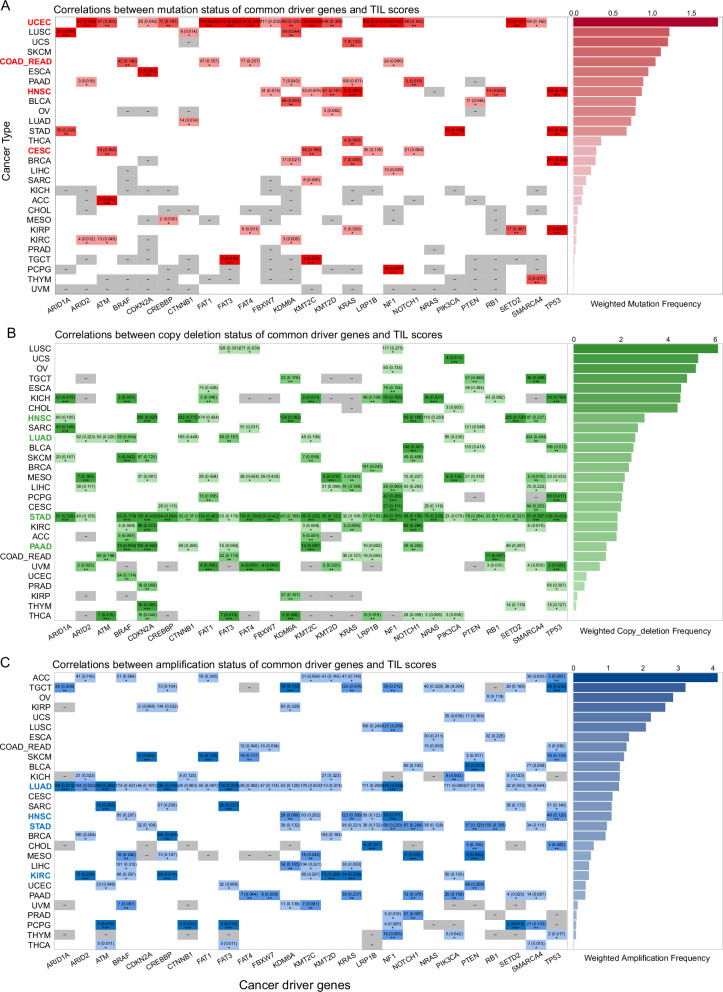
Effects of SNVs and CNVs of 25 cancer driver genes on TIL score distributions. **A** Effects of SNV on TIL score distributions. TIL scores were compared in SNV and non-SNV patient groups. **B** Effects of CNV (copy deletion) on TIL score distributions. TIL scores were compared in the deletion and non-deletion patient groups. **C** Effects of CNV (copy amplification) on TIL score distributions. TIL scores were compared in the amplification and non-amplification patient groups. Color-filled squares indicate significant differences in TIL score distributions for two groups (SNV and non-SNV, CNV and non-CNV) (*t*-test). Gray squares indicate the number of SNV and CNV cases for a gene in a cancer type is 0. Filled numbers represent the patient numbers and proportions of CNV or SNV for each gene in each cancer type. “*”, *P* < 0.05; “**”, *P* < 0.01; “***”, *P* < 0.001.

We systematically investigated the impact of SNV and CNV on TIL score distribution across all cancer types. Regarding the SNV status of 25 prominent cancer driver genes (Fig. 5A), UCEC exhibited the highest cumulative mutation frequency. SNVs in 16 out of 25 genes significantly influence TIL score distributions in UCEC. The four cancers most significantly affected by SNV were UCEC, COAD_READ, HNSC and CESC (Supplementary Fig. 1,0). A discernible trend emerged, indicating that patients harboring SNVs in common driver genes exhibited higher TIL scores than those without SNVs. However, patients with TP53 mutations displayed inconsistent TIL score distributions across different cancer types (Supplementary Fig. 11A). BRCA patients showed an increase in TIL scores, while HNSC, KIRP, and STAD patients exhibited decreased trends in TIL scores. The incidence of TP53-based co-mutations was generally low across all cancer types, with the highest cumulative mutation frequency observed in LUSC, reaching 0.3854 across 24 driver genes (Supplementary Fig. 11B). Among the top ten cancer types with the highest cumulative mutation frequency, 13 out of 24 TP53-based co-mutations resulted in a significant decrease in TIL scores in HNSC (Supplementary Fig. 11B, C). In the realm of CNVs, copy deletion (Fig. 5B) and amplification (Fig. 5C) of 25 driver genes significantly influenced TIL score distributions across different cancer types. LUSC and ACC exhibited the highest cumulative copy deletion frequency and cumulative amplification frequency, respectively. The top four cancers impacted by copy deletions were HNSC, LUAD, STAD and PAAD (Supplementary Fig. 12). Significant decrease of TIL score values was evident in 24 out of 25 genes in STAD, 8 out of 25 genes in LUAD, and 9 out of 25 genes in HNSC and PAAD. Similarly, the top four cancers impacted by amplifications were LUAD, HNSC, STAD, and KIRC (Supplementary Fig. 13). TIL scores were significantly lower in 20 out of 25 genes in LUAD, 10 out of 25 genes in STAD, as well as in 7 out of 25 genes in HNSC and KIRC. A discernible trend was observed that patients harboring CNVs in common driver genes exhibited lower TIL scores than those without SNVs. These findings reflect the potential influence of genome variation on shaping the tumor immune microenvironment.

### TIL scores improve patient risk stratification across different cancer types

As outlined in “Methods”, we employed clinical prognostic models to assess the influence of TIL scores on patient survival and their potential use in risk stratification. Cancer types were included in the analysis only if their TIL scores significantly impacted survival (Log-rank test *P*-value < 0.1) during the initial evaluation, as depicted in Fig. 3D. Cases with missing clinical feature values, especially stage and histological grade, were excluded. Furthermore, cancer types with fewer than 120 patients were also excluded. Subsequently, survival analyses were conducted for 10 cancer types to predict OS for BLCA, BRCA, CESC, HNSC, KIRC, LIHC, LUAD, PAAD, SARC, and SKCM, DSS for the same cancer types, and PFI for BLCA, BRCA, CESC, HNSC, KIRC, LIHC, SKCM, STAD, THCA, respectively. The random survival forest (RSF) technique was employed to train two distinct prognostic models: M1, which utilizes clinical data only, and M2, which combines clinical data with TIL scores.

**Fig. 6:**
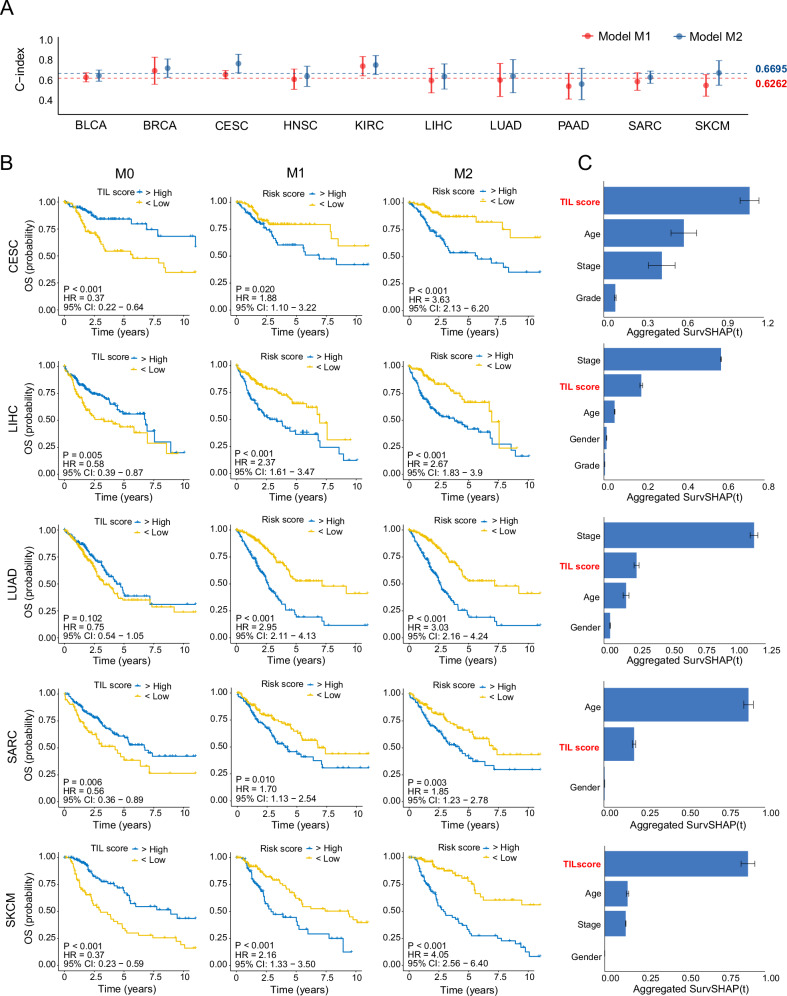
Performance of different prognostic models for predicting OS in CESC, LIHC, LUAD, SARC, and SKCM. M0, effects of TIL scores on OS; M1, prognostic models based on clinical data only; M2, prognostic models established by clinical data combined with TIL scores. **A** C-indices of M1 and M2 in each cancer type in a five-fold cross-validation. Horizontal lines indicate average C-indices across all cancer types for two types of models. **B** Kaplan–Meier curves of patient stratification for OS across different cancer types under different models. M0, Effects of TIL scores on OS. **C** The corresponding average aggregated SurvSHAP(t) values of each variable for M2 models. SurvSHAP(t) is a kind of time-dependent explanations of machine learning survival models. An aggregated SurvSHAP(t) value of one variable represents its importance measure in one case. Average aggregated SurvSHAP(t) value of one variable represents its global importance across all samples in the model.

Patients with high TIL scores demonstrated improved OS across all 10 cancer types, with statistical significance observed in 7 cancer types (BRCA, CESC, HNSC, KIRC, LIHC, SARC, and SKCM, log-rank test *P* < 0.05) (M0 in Fig. 6B and Supplementary Fig. 14A). M2 achieved an overall average C-Index of 0.6695, surpassing the average C-Index of 0.6262 for M1. This trend was consistent across all 10 cancer types, reflecting the superior performance of M2 in terms of C-Indices. Additionally, M2 exhibited substantial improvement in risk stratification for predicting OS in 9 out of the 10 cancer types (BLCA, CESC, HNSC, KIRC, LIHC, LUAD, PAAD, SARC, and SKCM) compared to M1 (M2 and M1 in Fig. 6B and Supplementary Fig. 14A). This improvement was reflected by increased HR values and decreased *P*-values. The average aggregated SurvSHAP(t) values highlighted the high importance of TIL scores (ranked in the top two among all variables) for M2 models in 6 out of the 10 cancer types (CESC, LIHC, LUAD, PAAD, SARC, and SKCM) (Fig. 6C and Supplementary Fig. 14B).

Similarly, patients with high TIL scores exhibited DSS benefits across all 10 cancer types, with statistical significance observed in all 10 cancer types (M0 in Supplementary Figs. 15B and 16A). M2 achieved an overall average C-Index of 0.6740, surpassing the average C-Index of 0.6401 for M1 (Supplementary Fig. 15A). The superiority of M2 models was evident in 9 out of the 10 cancer types (excluding STAD) in terms of C-Indices. Additionally, M2 models demonstrated significant improvement in risk stratification for predicting DSS in 9 out of the 10 cancer types (BLCA, BRCA, CESC, HNSC, KIRC, LUAD, PAAD, SARC and SKCM) compared to M1 models (M2 and M1 in Supplementary Figs. 15B and 16A). The average aggregated SurvSHAP(t) values revealed that TIL scores ranked high importance among all variables for M2 models in 6 out of the 10 cancer types (BRCA, CESC, LIHC, LUAD, SKCM, and STAD) (Supplementary Figs. 15C and 16B).

Patients with high TIL scores exhibited PFI benefits across all 9 cancer types, with statistical significance observed in 7 out of 9 cancer types (BLCA, BRCA, CESC, KIRC, SKCM, STAD and THCA, Log-rank test *P* < 0.05) (M0 in Supplementary Figs. 17B and 18A). M2 models achieved an overall average C-index of 0.6672, outperforming the average C-index of 0.6410 for the M1 models (Supplementary Fig. 17A). M2 models achieved higher C-Indices in 8 out of 9 cancer types (BLCA, BRCA, CESC, HNSC, KIRC, STAD, THCA, and UCEC). Moreover, M2 models significantly improved risk stratification in 8 out of 9 cancer types for predicting PFI (BLCA, BRCA, CESC, KIRC, PAAD, SKCM, STAD and THCA) (M2 and M1 in Supplementary Figs. 17B and 18A). The average aggregated SurvSHAP(t) values revealed that TIL scores ranked high importance among all variables for M2 models in 6 out of 9 cancer types (BLCA, BRCA, CESC, STAD, THCA and LIHC) (Supplementary Figs. 17C and 18B).

In summary, these findings imply that TIL scores significantly enhance patient risk stratification in most cancer types and can serve as a critical indicator for predicting survival (OS and DSS) and therapy response (PFI).

## Discussion

Tumor-infiltrating lymphocytes (TILs) are emerging as promising biomarkers for treatment responses and clinical outcomes in various cancer types. Manual assessment of TILs in hematoxylin-eosin slides according to the guidelines issued by The International Immuno-Oncology working group has become a standard practice in biomedical research, but this approach is laborious and subjective^32,45,46^. In this study, we address this limitation by developing TILScout, a fully automatic approach for analyzing TILs from WSIs. TIL scores generated by TILScout facilitate the evaluation of TIL infiltration extent in tumor tissues based on pathological images. We demonstrate the potential of TIL scores as a pan-cancer prognostic predictor and present the most comprehensive to date survey of TIL infiltration and its molecular as well as clinical correlates across 28 cancer types.

Compared to previously published tools (Supplementary Table 12), TILScout offers several advantages. It is the first integrated, fully automatic pan-cancer approach using original WSIs as the only input. This differs from previous studies that require either manual annotation or an additional machine learning model to initially identify cancer regions. TILScout has shown superior accuracy across various cancer types compared to other methods.

To validate the reliability and practicality of TIL scores for assessing the extent of TIL infiltration, we conducted comprehensive functional and correlational analyses. Gene set enrichment analysis revealed that all 52 top-upregulated gene sets/pathways associated with TIL scores were linked to the immune system across a broad spectrum of cancer types. These pathways covered different phases of the adaptive immune response. TIL scores demonstrated a significant positive correlation with lymphocyte fractions estimated using the CIBERSORT algorithm in over 95% of cancer types, primarily driven by the contribution of CD8+ T cells. Furthermore, TIL scores exhibited a positive correlation with immune scores in 67.8% of cancer types. These findings provide compelling evidence that TIL scores indeed reflect the extent of TIL infiltration in tumor tissues across diverse cancer types.

Immunotherapy has significantly transformed the therapeutic landscape in modern oncology, with immune checkpoint inhibitors (ICIs) such as anti-PD-1, anti-PD-L1, and anti-CTLA4 emerging as established standards of care for various tumor types, particularly at advanced stages. However, the clinical efficacy achieved with ICI treatments displays considerable variability. Presti et al^45^. conducted a systematic review of evidence about the role of TILs as a predictive biomarker of response to immunotherapy in solid tumors. The data provided primarily originates from translational sub-analyses conducted within the context of phase II and phase III randomized clinical trials, with a minimal proportion of clinical trials derived from observational studies. Patients with higher TIL density exhibited improved response to immunotherapy in most cancer types, especially in breast cancer and melanoma. However, TIL assessments in these studies relied on manual evaluation of either HE-stained slides or immunohistochemistry (IHC). Our study presents a convenient method for automatically calculating TIL scores from HE-stained slides, offering a user-friendly approach for clinical applications. Besides, according to our study, two crucial immune checkpoint genes, PDCD1 (PD-1) and CTLA-4, significantly correlate with TIL scores across most cancer types. Therefore, we expect TIL scores to be a potential predictor of immunotherapy response.

There has been a longstanding debate over whether driver mutations can potentially influence the cancer immune phenotype. So far substantial evidence establishing a connection between T cell immunity in cancer and the presence of driver genetic mutations has been lacking. Li et al.^47^ presented compelling evidence indicating that cancer-associated epigenetic driver mutations, exemplified by ARID1A mutations, can potentially shape tumor immune phenotype and contribute to immune evasion in various cancer types. Even though our study revealed no significant correlations between TIL scores and tumor mutation burden (TMB) in most cancer types, a noteworthy discovery emerged. For the first time, we observed that within most cancer types and common cancer driver genes, TIL infiltration is associated with the genomic variations of individual driver genes. Although the magnitude of these effects varies across different cancer types, a discernible trend emerged, indicating that patients harboring single nucleotide variations (SNVs) in individual driver genes tended to exhibit higher TIL scores than those without SNVs. Conversely, patients harboring copy number variations (CNVs) in individual driver genes exhibited lower TIL scores than those without CNVs. These findings might offer valuable insights for guiding clinical treatment decisions.

Elevated proportion of TILs has been associated with improved survival outcomes in various cancers^8,48–50^, but the prognostic significance of this effect remains a subject of controversy. In this context, we systematically investigated pan-cancer prognostic implications of TILs using TIL scores. Despite variations in the impact of TILs on prognosis among different cancer types, the high TIL scores group demonstrated a 32% reduction in the risk of death (OS), a 41% reduction in the risk of cancer-specific death (DSS), and a 26% reduction in the risk of cancer recurrence (PFI) across all cancer types. Furthermore, analyses based on clinical prognostic models revealed a noteworthy enhancement in risk stratification for predicting OS, DSS, and PFI through the incorporation of TIL scores in specific cancer types.

To conclude, we developed TILScout, which uses deep learning for the automatic classification of WSI patches and the computation of TIL scores. The reliability and practicality of TIL scores in evaluating the extent of TIL infiltration across various cancers were validated through comprehensive analyses encompassing both qualification and quantification. Additionally, our investigation revealed that TIL infiltration is associated with genomic variations in common cancer driver genes in tumor tissues, offering potential therapeutic insights for patients with the corresponding genomic variations. Our comprehensive pan-cancer survey indicated that TIL scores could serve as a potential prognostic indicator and predictor of anti-tumor response in different cancer types, although in some cancers their predictive value may be limited by sample size and the tumor microenvironment’s heterogeneity. Despite the promising results, the predictor requires validation in real-world clinical practice to ensure its effectiveness and reliability.

## Methods

### Whole-slide images (WSIs) and multi-omic data acquisition

We obtained 10,029 Hematoxylin and Eosin (H&E)-stained histopathology WSIs from the Cancer Genome Atlas (TCGA) database across 28 solid cancer types via the Genomic Data Commons (GDC; https://portal.gdc.cancer.gov/). Three additional WSIs datasets were obtained from Clinical Proteomic Tumor Analysis Consortium Lung Adenocarcinoma (CPTAC-LUAD, 47 WSIs), Lung Squamous Cell Carcinoma (CPTAC-LUSC, 50 WSIs) cohorts (https://www.cancerimagingarchive.net/), and Radboud University Medical Center Breast Cancer (RUMC-BRCA, 48 WSIs) cohort. TCGA provides images for two types of slides: flash-frozen and formalin-fixed paraffin-embedded (FFPE) tissue slides. Only FFPE tissue slide images were taken into consideration due to the extremely low quality of flash-frozen slide images. WSIs with bubbles, overlapping tissues, poor staining and artificial markings were manually excluded from consideration. Corresponding clinical, genomics, and transcriptomics for each patient were obtained via GDC. Clinical data included age, gender, histological grade, and cancer stage. We also obtained data on overall survival (OS), disease-specific survival (DSS), and progression-free interval (PFI)^51^ from UCSC Xena (https://xena.ucsc.edu/).

### WSIs processing

Figure 1 outlines the workflow of WSI processing. Each WSI (at ×20 magnification) was automatically segmented into thousands of patches of 150 × 150 pixels without overlap using the open-source library OpenSlide in python. Each patch represents an RGB image, and each pixel combines three color channels - red, green, blue. The values of each channel range from 0 to 255. Patches with average values of RGB > 230 ((Vred + Vgreen + Vblue)/3 > 230) and the corresponding standard deviation values < 15 were considered blank and ignored. This step is required to ensure that the included patches have enough tissues for classification and model training. Then selected patches were color-normalized with Macenko’s method^52^ to eliminate the influence of subtle differences caused by different hues in different HE images.

### Classification of patches and dataset preparation

Our multiclass classification strategy is similar in spirit to the approach described by Chen et al.^20^. For each patch with a size of 150 × 150 pixels, we defined a patch as TIL-positive if it contained at least 3 detected lymphocytes and at least 1 detected tumor cell, TIL-negative if it contained at least 1 detected tumor cell and less than 3 detected lymphocytes, non-tumor/necrotic if it did not contain any tumor cells, regardless of the number of lymphocytes. This labeling strategy and classification approach differ significantly from other studies that did not explicitly define the number of lymphocytes required to classify a patch as TIL-positive^29,38,40^ or just focused on marking only the central area of a patch (sub-patch) of a fixed size^39^. Our assessment of TILs does not distinguish between stromal TILs (sTILs) and intratumoral TILs (iTILs), as each patch may contain both types, and our classification approach inherently considers the overall presence of lymphocytes without separating them based on their specific location.

Out of millions of patches available in our dataset (Supplementary Table 1), representative patches were manually selected and labeled by three experienced pathologists in which both tumor cells and lymphocytes exhibited clear and distinct morphologies. Our initial goal was to construct a dataset of approximately 90,000 patches covering 28 cancer types from TCGA database. However, 12 cancer types with insufficient number of cases (such as CHOL, ACC, UCS, UVM, MESO, PAAD, PCPG, ESCA, TGCT, THYM, KICH, and OV) were merged into three groups—CAUM, PAPE, and TTKO—leaving us with 19 cancer types and approximately 90,000/19 = 4750 patches targeted for each cancer type on average. We aimed to maintain a consistent ratio of three patch labels in each tumor type at 1:1.5:1.5, inspired by a previous study^29^ in which the 1:3 ratio of positive patches to all patches was shown to be optimal. This adjustment might decrease the false-positive rate. In instances where the number of patches for a specific label was insufficient for a particular cancer type—often due to significant variance in TIL distribution across different cancers, resulting in a scarcity of TIL-positive patches—we compensated this lack of data by increasing the number of patches under the same label in other cancer types to preserve the overall dataset’s label ratio of approximately 1:1.5:1.5. Furthermore, efforts were made to include as many WSIs as possible to ensure comprehensive coverage of various cancer types. In total, 90,488 patches were ultimately selected from an initial pool of 102,538,441 patches generated from 2487 WSIs across 28 cancer types. The distribution of labeled patches was as follows: TIL-positive (22,148), TIL-negative (34,186), and non-tumor/necrotic (34,154). Detailed information on dataset generation is available in Supplementary Table 1 and Data 2. To select the best-performing model, patches for each label were randomly split into an initial training and validation dataset with an 8:2 ratio (Supplementary Fig. 1). To create an independent test set, 9000 patches (3000 for each cancer type: RUMC-BRCA, CPTAC-LUAD, CPTAC-LUSC) (TIL-positive: 677, TIL-negative: 4345, non-tumor/necrotic: 3978) were randomly selected from 145 WSIs and labeled. Detailed information on the independent test set generation is available in Supplementary Table 2.

### Machine learning models

In order to select an optimal model for the prediction of TIL scores, we tested a number of deep-learning approaches, including supervised learning by 8 pre-trained neural networks (VGG16^53^, VGG19^53^, ResNet34^54^, ResNet50^54^, Xception^55^, InceptionV3^56^, InceptionResNetV2^57^), UNI^58^ and unsupervised learning by K-means clustering and Convolutional Autoencoder^59^. For the pre-trained neural networks, we utilized models initialized with parameters trained on ImageNet, updating only the final layers to leverage their feature extraction capabilities. Custom fully connected layers were appended, including a dense layer with 512 units and an output layer with 3 units, adapted for our three-class classification task.

For supervised learning models, the parameters of each network were initialized using the pre-trained model. Patches were resized to the neural network input size (224 × 224 pixels). The output of the last layer of the networks corresponds to the probabilities of three classes: TIL-positive, TIL-negative, non-tumor/necrotic regions. We calculated the cross-entropy loss between the predicted and the actual labels using the function “sparse_categorical_crossentropy” from the Keras package and used the “adaptive moment estimation” as a fast optimizer, with the number of epochs set to 50 and the EarlyStopping callback to 4. The best model was automatically saved according to improved loss value during the training process. We compared each network’s accuracy and loss values (Supplementary Fig. 2) and selected InceptionResNetV2 as the classification network for subsequent analysis.

We tested a convolutional Autoencoder-based K-means strategy for deep clustering. Convolutional autoencoders (CAEs) are mainly applied for compressing the input images while keeping most of the essential information, and extracting robust features^60^. Patches were resized (152 × 152 pixels) to fit the convolutional Autoencoder model. The architecture of the convolutional Autoencoder model is shown in Supplementary Table 3. We calculated the loss using the function “mean_squared_error” and used the “adaptive moment estimation” as a faster optimizer. The number of epochs was set to 50. Then the compressed features were extracted from the model for K-means clustering. Manual labeling and analysis were then conducted based on unsupervised classification results.

### Iterative manual improvement of patch labels and final model training

After initial model training and validation, we selected InceptionResNetV2 as the classification network for subsequent analyses. According to the confusion matrix and the error plot, we selected patches for which the prediction results did not match the actual labels. These potentially erroneously labeled patches were reviewed and relabeled by pathologists. We implemented a double-blind re-labeling strategy involving multiple annotators to mitigate subjective bias. Specifically, the pathologists performing the re-labeling were unaware of the model’s predictions for these patches. After the iteration of manual improvement, the data within each label in each cancer type were randomly split into the training and validation set with an approximate ratio of 8:2, ultimately resulting in a dataset comprising 72,272 patches for training and 18,216 patches for validation (Supplementary Fig. 1). The training set was used for model training and 10-fold cross-validation, while the validation set and independent test set were used for performance evaluation of the final model. Special care was taken to ensure that information gained during the model selection stage (e.g., feature distributions or patterns) does not influence the final training and evaluation stages. First, the experimental design is structured as two relatively independent steps. The dataset was completely re-split after label correction, and the final training phase only used the new splits. This ensures that the model selection process is insulated from the subsequent training phase. Secondly, the final model is trained from scratch, ensuring that no learned parameters or information from the model selection step influence the training process. Additionally, the test set remains strictly independent and was not used during model selection, training, or validation phases.

### Calculation of TIL scores and construction of TIL maps for all patients

For a given WSI a TIL score is the ratio between the number of predicted TIL-positive patches to the sum of the number of predicted TIL-positive and TIL-negative patches obtained by the trained InceptionResNetV2 model. For each patient with more than one WSI, the TIL score is calculated as the ratio of the sum of TIL-positive patches to the sum of both TIL-positive and TIL-negative patches across all WSIs:

1 $${\rm{TIL}}\,{\rm{Score}}=\frac{\mathop{\sum }\nolimits_{i=1}^{{\rm{m}}}{{\rm{N}}}_{{\rm{TIL}}-{\rm{positive}}}^{i}}{\mathop{\sum }\nolimits_{i=1}^{{\rm{m}}}({{\rm{N}}}_{{\rm{TIL}}-{\rm{positive}}}^{i}+{{\rm{N}}}_{{\rm{TIL}}-{\rm{negative}}}^{i})}$$

where *m* is the number of WSIs for each patient, N_TIL-positive_ is the total number of predicted TIL-positive patches in each WSI, and N_TIL-negative_ is the total number of predicted TIL-negative patches in that WSI.

According to the prediction results we constructed TIL maps, which display the patch-level distribution of TILs across each WSI. Figure 2E presents two examples of TIL maps.

### TME risk scores for each type of cancer

We have previously developed a tumor microenvironment-related risk (TME risk) scoring system for predicting the overall survival (OS) of LUAD patients^61^ based on gene expression levels in the TME. In this work we constructed TME risk scores for the cancer types with more than 100 cases in a similar fashion.

TPM normalized RNA-seq data and clinical data for all patients were downloaded via the GDC (https://portal.gdc.cancer.gov/repository). Immune and stromal scores for each patient were calculated using the ESTIMATE algorithm^62^. Patients were subdivided into groups with high and low immune and stromal scores according to the optimal cut-off point associated with survival differences determined by maximally selected rank statistics using the Log-rank test, as implemented in the R package maxstat. To ensure close sample sizes of the two groups, the sample ratio was controlled such that each of the two groups contained at least 40% and no more than 60% of all samples (maxstat parameters minprop = 0.4 and maxprop = 0.6). Supplementary Fig. 3 illustrates optimal cut-off point selection and the outcome distributions for one cancer type (BLCA). Differentially expressed genes (DEGs) between the high and low stromal score group as well as between high and low immune score group were identified using the R package limma with the screening criteria of logFoldChange > 1 and adjusted *P*-value < 0.05. Weighted gene co-expression network analysis (WGCNA; Langfelder et al.^63^) was used to identify co-expressed gene modules strongly related to the immune and stromal scores using the R package WGCNA (Supplementary Fig. 4A). Gene lists obtained by differential expression analysis and WGCNA were merged to create the final list of TME-related genes (Supplementary Fig. 4A).

Least absolute shrinkage and selection operator (LASSO) regression analysis as implemented by the glmnet R package^64^ was conducted to identify TME-related genes whose expression levels were significantly associated with patient survival. The individual impact of each gene on survival was assessed by univariate Cox regression analysis and for genes with a *P*-value less than 0.1 (to identify as many variables that could impact survival as possible), risk coefficients were obtained by multivariate Cox regression analysis.

For each cancer type a TME risk score (Supplementary Fig. 4B) was calculated as:

2 $${\rm{TME}}\,{\rm{risk}}\,{\rm{score}}=\mathop{\sum }\limits_{i=1}^{{\rm{k}}}{\beta }_{i}\ast {\mathrm{Exp}}(i)$$

where *k* is the number of genes with a *P*-value less than 0.1 (ensuring that more potentially important variables are considered) selected by univariate Cox regression analysis, *β*_*i*_ is the risk coefficient of gene *i* determined by multivariate Cox regression, and Exp(*i*) is the expression value of gene *i*. We developed three separate TME risk scoring systems for predicting the overall survival (OS) (Supplementary Tables 4 and 5), disease-specific survival (DSS) (Supplementary Tables 6 and 7), and progression-free interval (PFI) (Supplementary Tables 8 and 9) for each type of cancer.

### Genomic data

Single nucleotide variation (SNV) data were downloaded from the TCGA database using the R package TCGAbiolinks^65^. The tumor mutational burden (TMB) value for each patient was computed by the R package maftools^66^. Copy number variation (CNV) data, encompassing information about deletions and amplifications, were obtained for all TCGA cohorts from the UCSC Xena (https://xena.ucsc.edu). The SNV data (containing two groups: wild and mutation for each gene) and CNV data (containing two groups: normal and copy deletion/amplification for each gene) for 25 prominent cancer driver genes, as annotated in the Cancer Gene Census^67,68^ were collated and extracted. Additionally we analyzed mutations occurring in the TP53 gene along with mutations in other genes within the same cells or tissues (TP53-based co-mutations), which play a crucial role in the pathogenesis, biology, microenvironmental interactions, and prognosis of cancers^69^.

### Gene set enrichment analysis

The top 2000 genes with the highest Spearman correlation between expression values and TIL scores across all samples were selected and ranked according to the correlation coefficients for subsequent gene set enrichment analysis by GSEA^70^. Although the authors of GSEA recommend using gene lists containing up to 500 entries as a general guideline (docs.gsea-msigdb.org/), we increased this number to 2000 genes to account for the heterogeneity of expression profiles in different cancers. Gene set enrichment analysis was conducted on this ranked list for three types of gene annotation—GO (gene ontology) terms^71,72^, hallmark gene sets^70,73^,-and Reactome^74^ pathways—using the R packages clusterProfiler^75,76^ and ReactomePA^77^. Significantly enriched annotations were identified based on the following thresholds: normalized enrichment score (NES) > 2 and nominal *P*-value < 0.05.

### Comprehensive analysis of TIL scores

Statistical association between TIL scores and clinical features obtained from TCGA (age (<65 and >65), gender (male and female), histological grade (high grade and low grade, or G1, G2, G3, and G4), and cancer stage (stage I, stage II, stage III, and stage IV)) was analyzed utilizing the Kruskal–Wallis test. We also computed Pearson correlation between TIL scores and multiple parameters, including TMB values, TME risk scores, and expression values of common immune checkpoint genes (ICGs)^78^. Putative proportions of different immune cell types for all samples were estimated based on gene expression levels using the CIBERSORT algorithm^79^. We then conducted a Pearson correlation analysis to confirm the consistency between TIL scores and established lymphocyte fractions. Furthermore, we computed the Pearson correlation coefficients between TIL scores and tumor immune dysfunction and exclusion (TIDE) scores^80^ as well as immunophenoscores (IPS)^81^. These scores serve as reliable surrogate biomarkers for predicting the potential response to immunotherapy. To investigate the impact of CNV and SNV on the extent of lymphocyte infiltration within the TME, a systematic assessment of TIL score distribution differences under different states of SNV (wild and mutation) and CNV (normal and copy deletion, normal and copy amplification) for 25 prominent cancer driver genes was conducted. A *P*-value of less than 0.05 is considered statistically significant.

### Pan-cancer survival analysis of TIL scores

Forest plots were used to conduct survival analysis (OS, DSS and PFI) of high and low TIL score groups across 28 cancer types. To investigate the predictive performance of TIL scores for patient risk stratification, prognostic models were constructed by integrating TIL scores with clinical parameters such as age, gender, stage, and histological grade in selected cancer types (cancer types with fewer than 100 cases and for which the TIL score had no significant effect on survival (*P* > 0.1, log-rank test) were excluded). Firstly, the effects of TIL scores on survival were assessed separately (model M0). Secondly, random survival forest (RSF) was employed to establish prognostic models using the Python module scikit-survival. The average Harrel’s concordance index (C-index)^82^ over the five-fold cross-validation was used to compare the performance of different models. Two types of models were established for each selected cancer type: M1, using clinical data only, and M2, using clinical data combined with TIL scores. Cox proportional hazard regression analysis was performed to obtain the risk coefficients of each variable in the models, which were used to calculate risk scores for patient risk stratification. Patients were stratified into groups with high- and low risk scores according to the optimal cut-off point associated with survival differences determined by maximally selected rank statistics using the Log-rank test as mentioned above. Kaplan–Meier curves were generated to compare the survival rates and visualize patient stratification between low- and high-risk groups for each cancer type, using *P*-values less than 0.05 as the significance level. We also calculated and visualized average aggregated SurvSHAP(t)^83^ values for variables in the model, which provide explanations of the whole distribution of each variable in the context of the survival function.

All steps of WSI processing, analysis, and deep learning were implemented using Python (version 3.9.3) and run on the Intel 64 architecture server with 64-core CPUs and the NVIDIA A40 GPU. Python packages included openslide (version 1.2.0), tensorflow (version 2.10.0), scikit-learn (version 1.2.1), pandas (version 1.4.4), matplotlib (version 3.7.0), and numpy (version 1.23.5). OMICs data processing and TIL score-related analysis were implemented using the R packages limma (version 3.50.3), estimate (version 1.0.13), survival (version 3.5–5), survminer (version 0.4.9), maxstat (version 0.7–25), pheatmap (version 1.0.12), WGCNA (version 1.72-1), glmnet (version 4.1–7), and clusterProfiler (version 4.1.1).

## Supplementary information

Supplementary Material

Supplementary Data 2

Supplementary Data 3

## Data Availability

The TCGA WSIs data and corresponding clinical, genomics, and transcriptomics can be downloaded via the GDC(https://portal.gdc.cancer.gov/). The CPTAC-LUAD and CPTAC-LUSC WSI data (https://cancerimagingarchive.net), and the RUMC-BRCA WSI data (https://breastpleomorphism.grand-challenge.org/) are publicly available. All datasets related to model training and evaluation can be accessed via the link: https://zenodo.org/records/14628242.

## Code availability

All code and the trained model related to TILScout are available via the GitHub repository.
