# Supplementary material for: Prediction and analysis of tumor infiltrating lymphocytes across 28 cancers by TILScout using deep learning
Source: NPJ Precis Oncol. 2025 Mar 19;9:76. doi: 10.1038/s41698-025-00866-0 (PMC11923303; doi:10.1038/s41698-025-00866-0)
Supplement: Supplementary file 1 — Supplementary Material [file 41698_2025_866_MOESM1_ESM.pdf]

Supplementary Material

Supplementary data 1. Abbreviations of TCGA cancer types in this study

ACC Adrenocortical carcinoma
BLCA Bladder urothelial carcinoma
BRCA Breast invasive carcinoma
CESC Cervical squamous cell carcinoma and endocervical adenocarcinoma
CHOL Cholangiocarcinoma
COAD_READ Colon adenocarcinoma and rectum adenocarcinoma
ESCA Esophageal carcinoma
HNSC Head and Neck squamous cell carcinoma
KICH Kidney Chromophobe
KIRC Kidney renal clear cell carcinoma
KIRP Kidney renal papillary cell carcinoma
LIHC Liver hepatocellular carcinoma
LUAD Lung adenocarcinoma
LUSC Lung squamous cell carcinoma
MESO Mesothelioma
OV Ovarian serous cystadenocarcinoma
PAAD Pancreatic adenocarcinoma
PCPG Pheochromocytoma and Paraganglioma
PRAD Prostate adenocarcinoma
SARC Sarcoma
SKCM Skin Cutaneous Melanoma
STAD Stomach adenocarcinoma
TGCT Testicular Germ Cell Tumors
THCA Thyroid carcinoma
THYM Thymoma
UCEC Uterine Corpus Endometrial Carcinoma
UCS Uterine Carcinosarcoma
UVM Uveal Melanoma

Supplementary Data 2. Patch Counts Based on WSIs across different cancer types

Supplementary Data 3. GSEA results

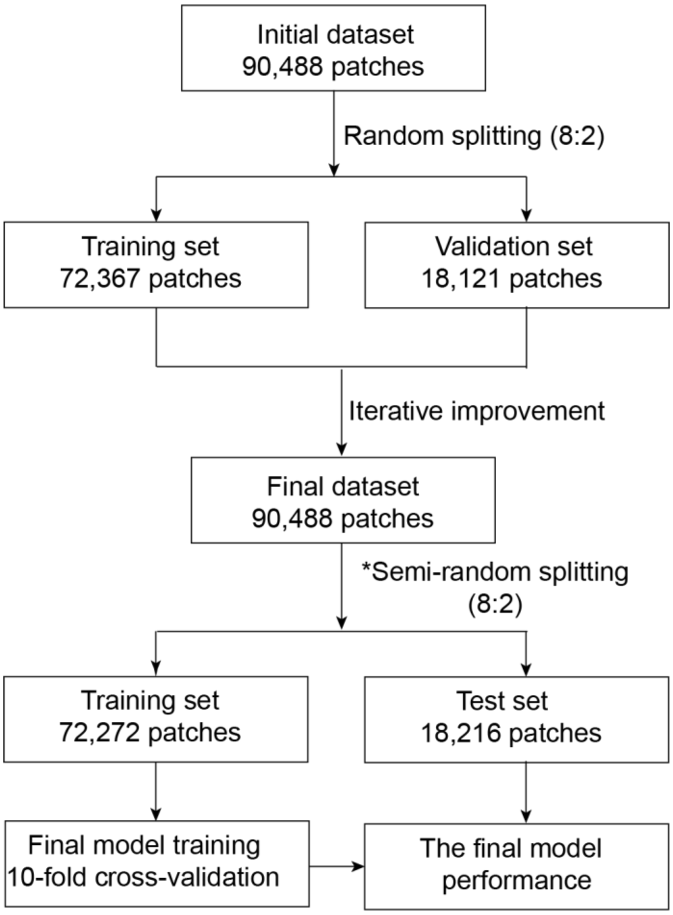

Supplementary Figure 1. Dataset generation process for model training

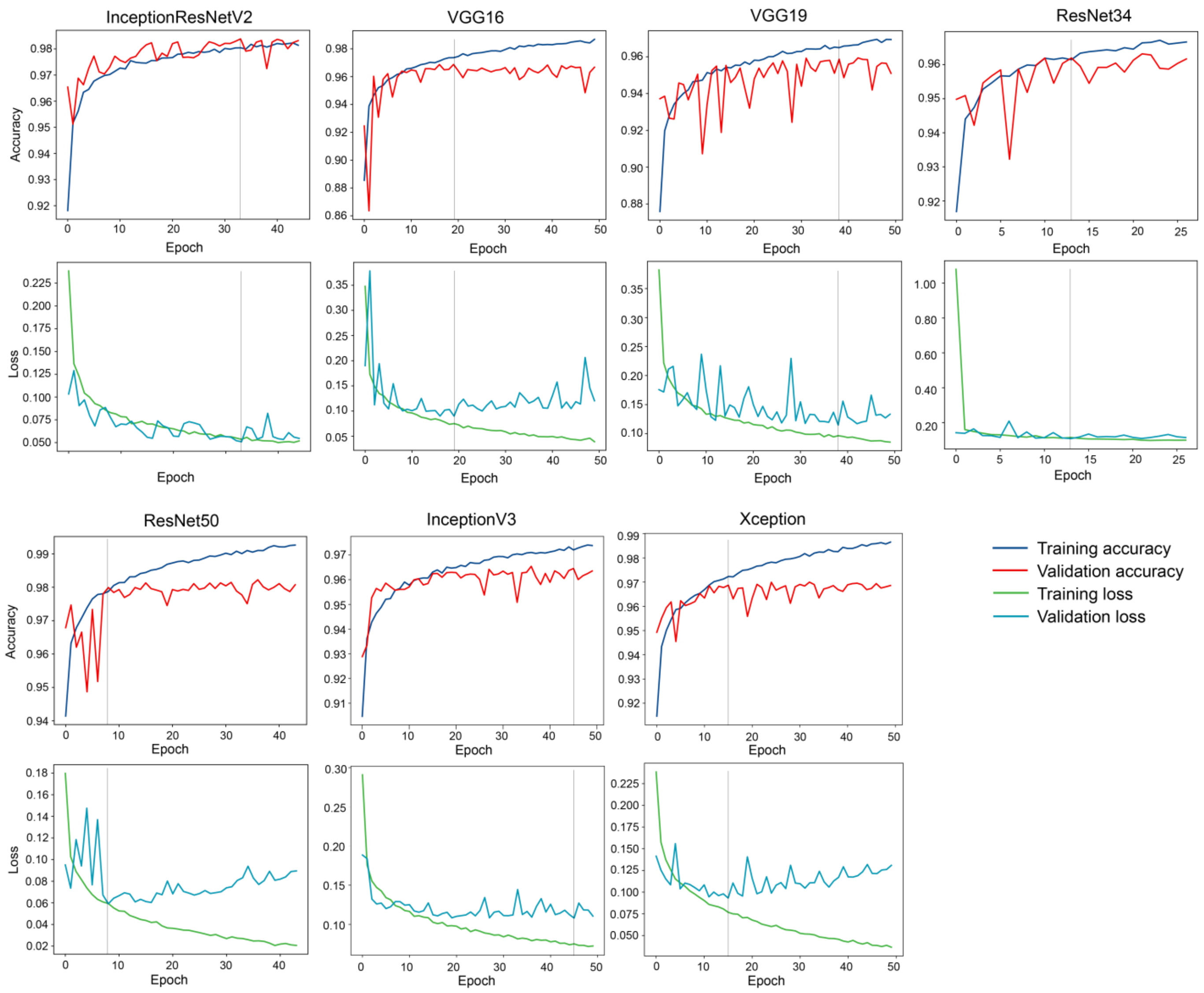

Supplementary Figure 2. Model performances of 7 convolutional neural networks during model training. The vertical solid line represents the position corresponding to the highest accuracy and lowest loss value of a model on the validation set and also represents the final performance of this model on the validation set.

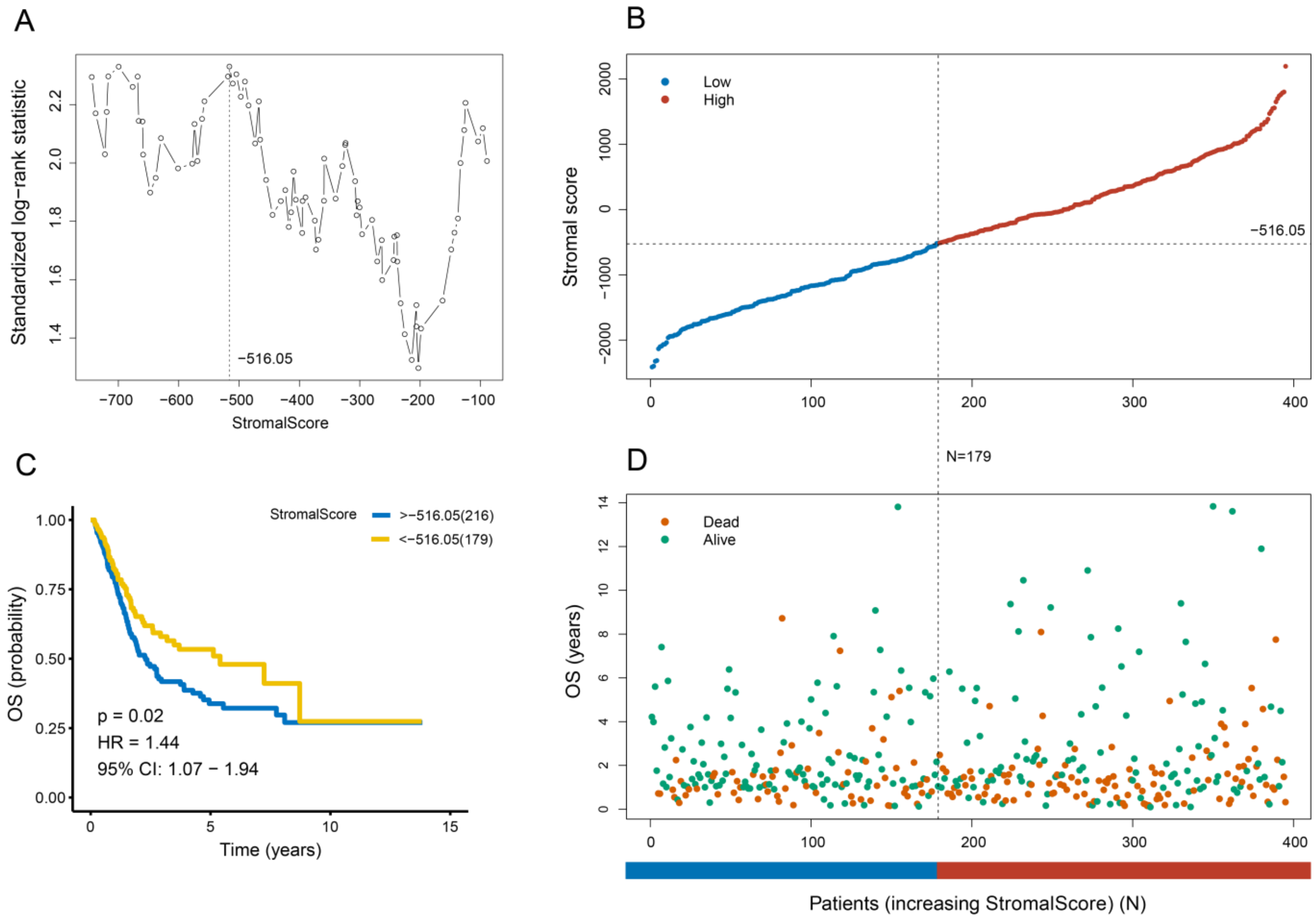

Supplementary Figure 3. An example of sample grouping for differential expression analysis in BLCA based on OS. A, the optimal cut-off point selection of stromal scores according to maximally selected rank statistics. B, corresponding sample sizes of the low- and high-score groups based on the optimal cut-off point. C, survival curves of two groups based on the optimal cut-off point. D, outcome distributions of two groups based on the optimal cut-off point.

BLCA

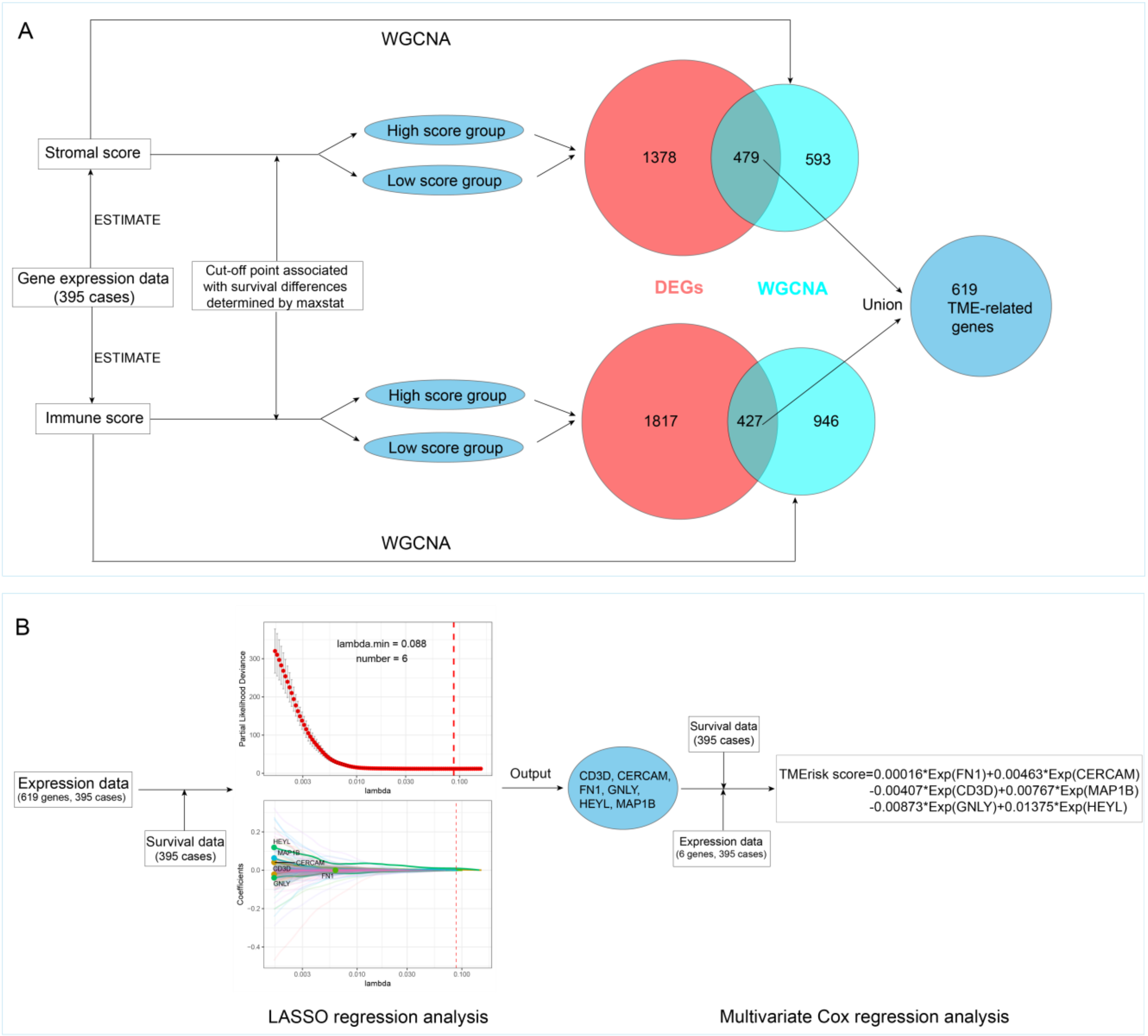

Supplementary Figure 4. An example of establishing TME risk scores in BLCA for predicting OS. A, procedures for screening TME-related genes. Firstly, patients were subdivided into groups with high and low immune and stromal scores according to the optimal cut-off point associated with survival differences. Secondly, DEGs between the high and low stromal/immune score groups and co-expressed gene modules strongly related to the immune and stromal scores were identified. Overlapped Gene lists of DEGs and WGCNA of stromal and immune groups were merged to generate the final list of TME-related genes. B, Genes selection for establishing TME risk score. LASSO regression analysis was conducted to identify TME-related genes whose expression levels were significantly associated with patient survival. Genes with a P value less than 0.1 assessed by univariate Cox regression analysis were finally used for establishing the TME risk score. Lambda(λ), the regularization parameter controlling the overall strength of the penalty; Partial likelihood deviance is used for evaluating the fit performance of the Cox regression model to the observed survival data, with a lower deviance indicating a superior fit of the model. In the background of LASSO Cox regression analysis, the value of partial likelihood deviance is identical to the mean cross-validated error(cvm); lambda.min (corresponding to the red dashed lines), the value of λ that gives minimum cvm, which determines the number of selected genes; Coefficients, LASSO coefficient profiles of genes associated with survival. The Exp(*i*) represents the expression value of gene *i*. The number before the Exp(*i*) represents the risk coefficient (β_i) of gene *i*.

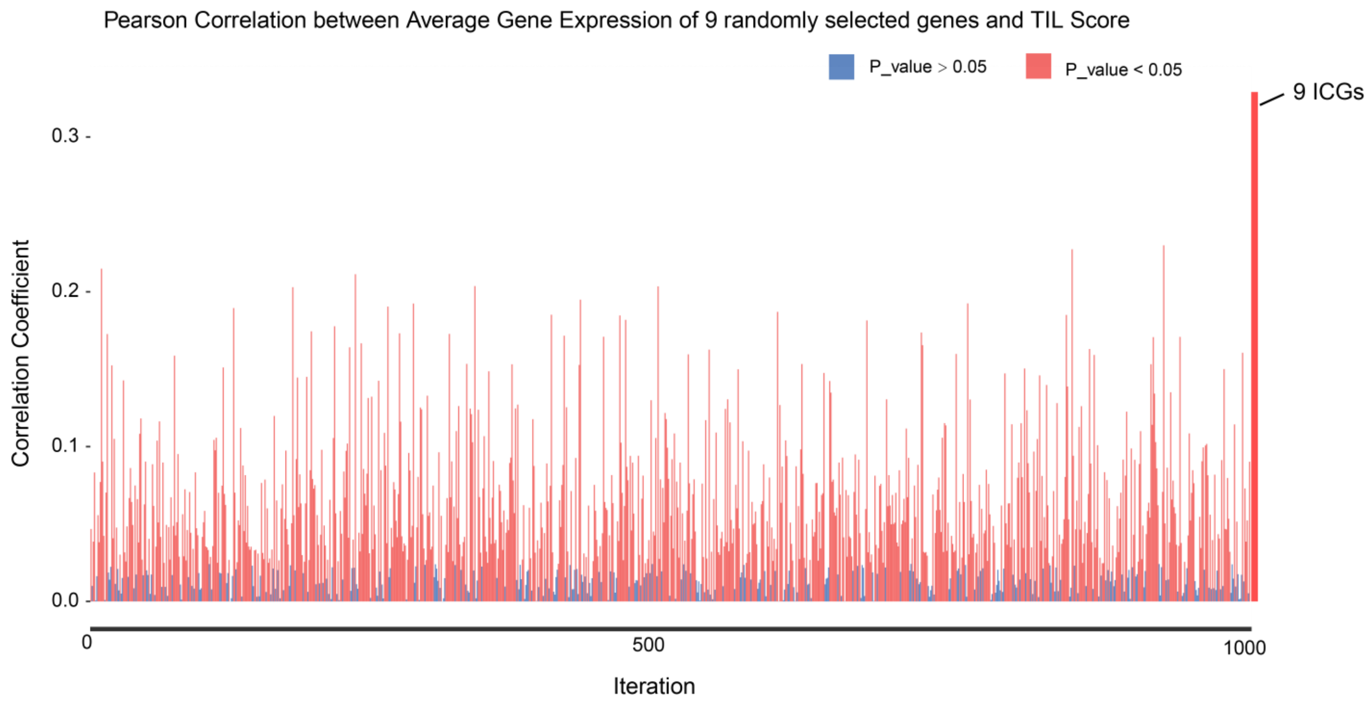

Supplementary Figure 5. Pearson Correlation between Average Gene Expression of 9 randomly selected genes and TIL Score. The horizontal coordinate indicates that 9 genes are randomly selected for 1000 iterations. The ordinate represents the Pearson correlation coefficient between TIL scores and 9 randomly selected genes. Red vertical lines indicate significant relationships(P<0.05).

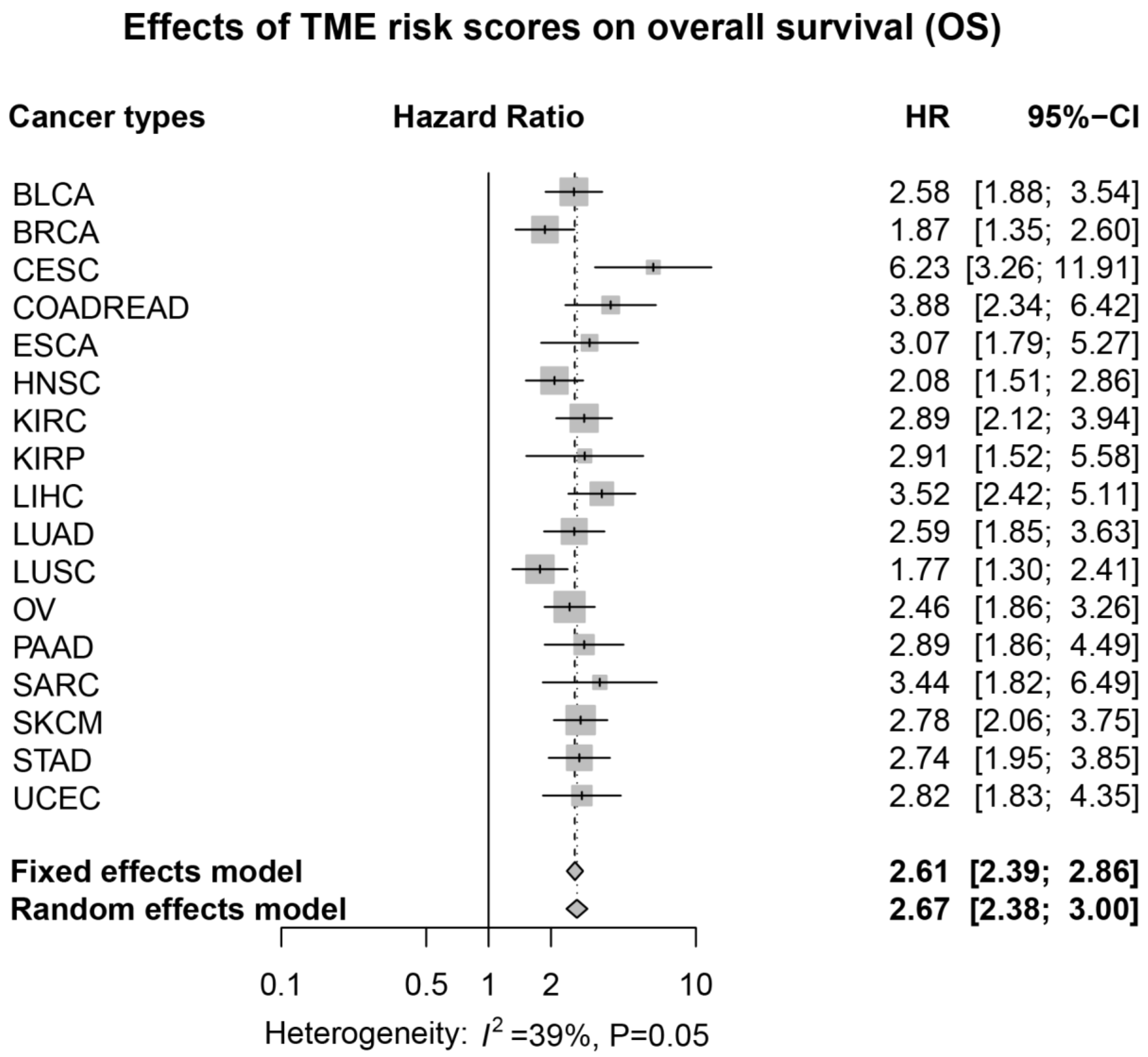

Supplementary Figure 6. Forest plots for the effects of OS-based TME risk scores on OS. The values of Hazard Ratio (HR) are delimited by 1. If HR value is less than 1, it means that TIL scores decrease the risk of death; if HR is greater than 1, it means that high TIL scores increase the risk of death. *I^2:* Inter-group heterogeneity test index. A P>0.05 means that there is no significant heterogeneity among the different cancer types; thus, the fixed effects model would be considered. Otherwise, the random effects model would be considered. The size of the square typically represents the weight or contribution of each cancer type to the overall effect estimate.

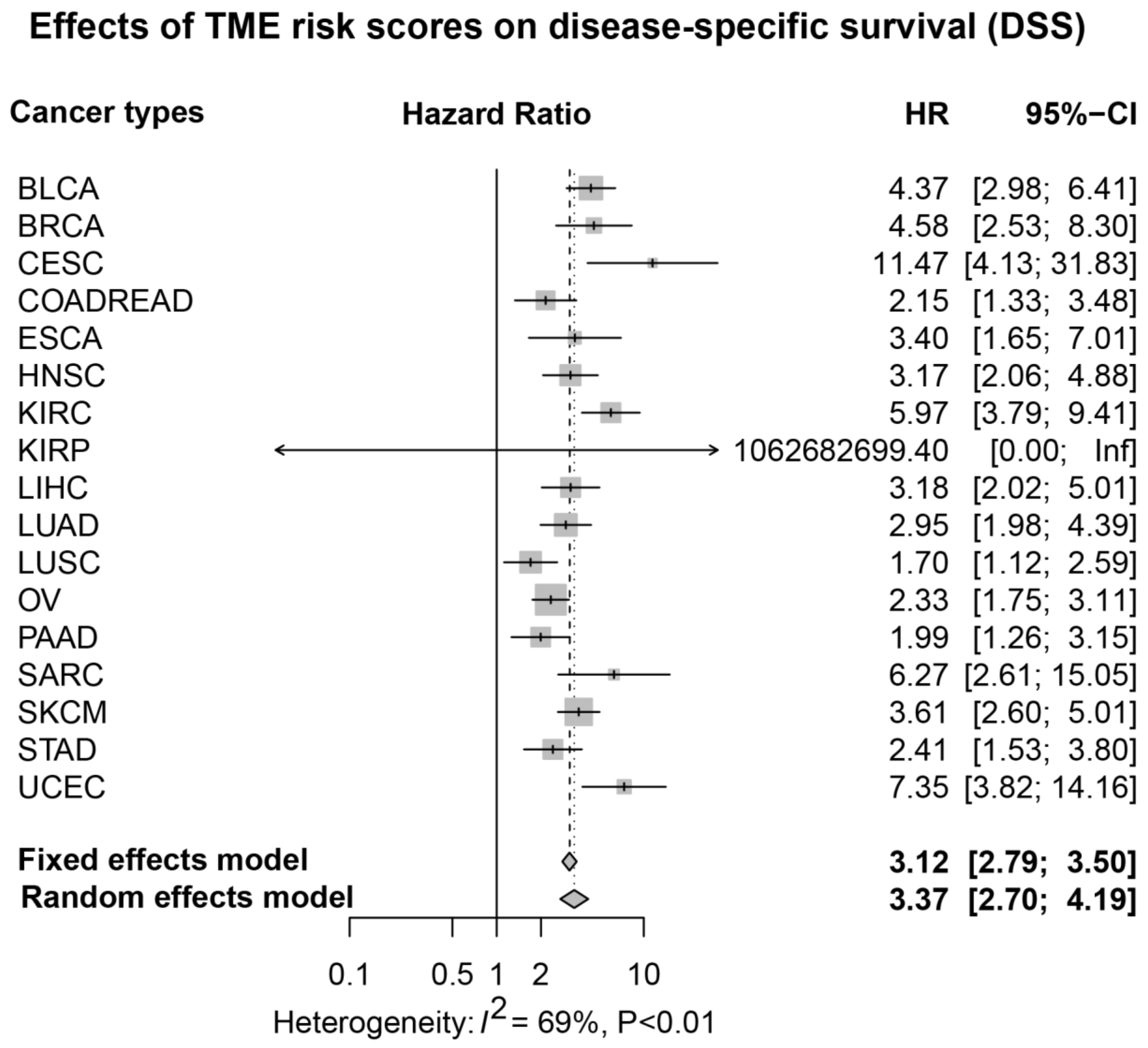

Supplementary Figure 7. Forest plots for the effects of DSS-based TME risk scores on DSS. The values of Hazard Ratio (HR) are delimited by 1. If HR value is less than 1, it means that TIL scores decrease the risk of cancer-specific death; if HR is greater than 1, it means that high TIL scores increase the risk of cancer-specific death. *I^2:* Inter-group heterogeneity test index. A P>0.05 means that there is no significant heterogeneity among the different cancer types; thus, the fixed effects model would be considered. Otherwise, the random effects model would be considered. The size of the square typically represents the weight or contribution of each cancer type to the overall effect estimate.

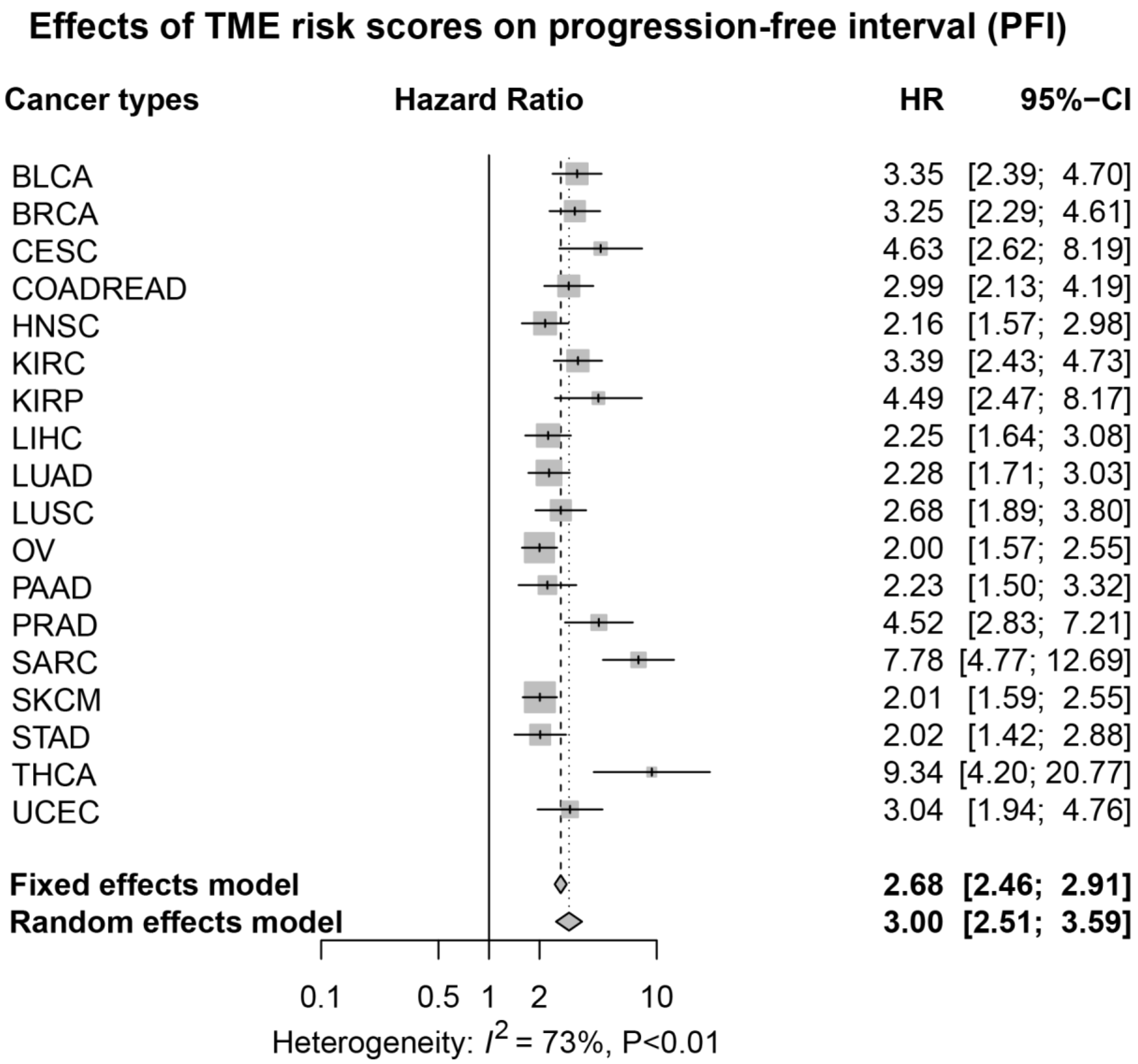

Supplementary Figure 8. Forest plots for the effects of PFI-based TME risk scores on PFI. The values of Hazard Ratio (HR) are delimited by 1. If HR value is less than 1, it means that TIL scores decrease the risk of recurrence; if HR is greater than 1, it means that high TIL scores increase the risk of recurrence. *I^2:* Inter-group heterogeneity test index. A P>0.05 means that there is no significant heterogeneity among the different cancer types; thus, the fixed effects model would be considered. Otherwise, the random effects model would be considered. The size of the square typically represents the weight or contribution of each cancer type to the overall effect estimate.

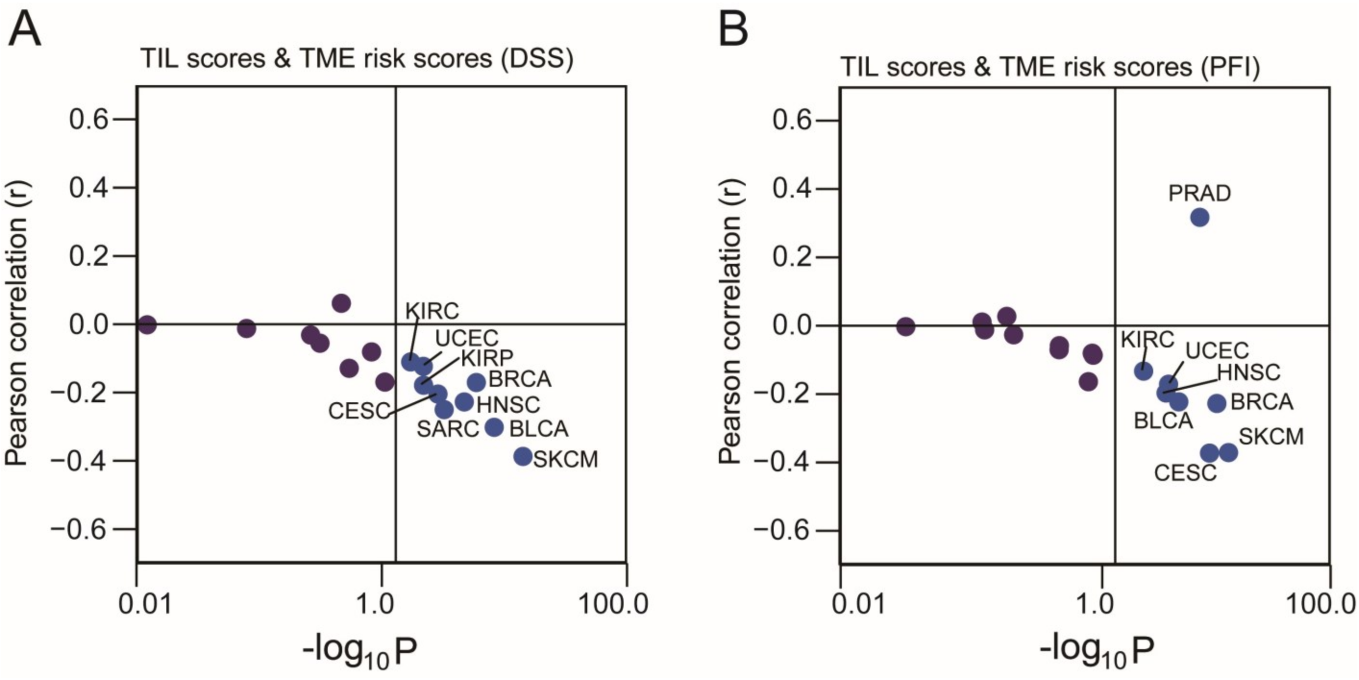

Supplementary Figure 9. Pearson correlations between TIL scores and DSS- and PFI-based TME risk scores. A, Pearson correlations between TIL scores and DSS-based TME risk scores. B, Pearson correlations between TIL scores and PFI-based TME risk scores. The vertical solid line is the dividing line where the P value equals 0.05. Cancer types with $p < 0.05$ were labeled.

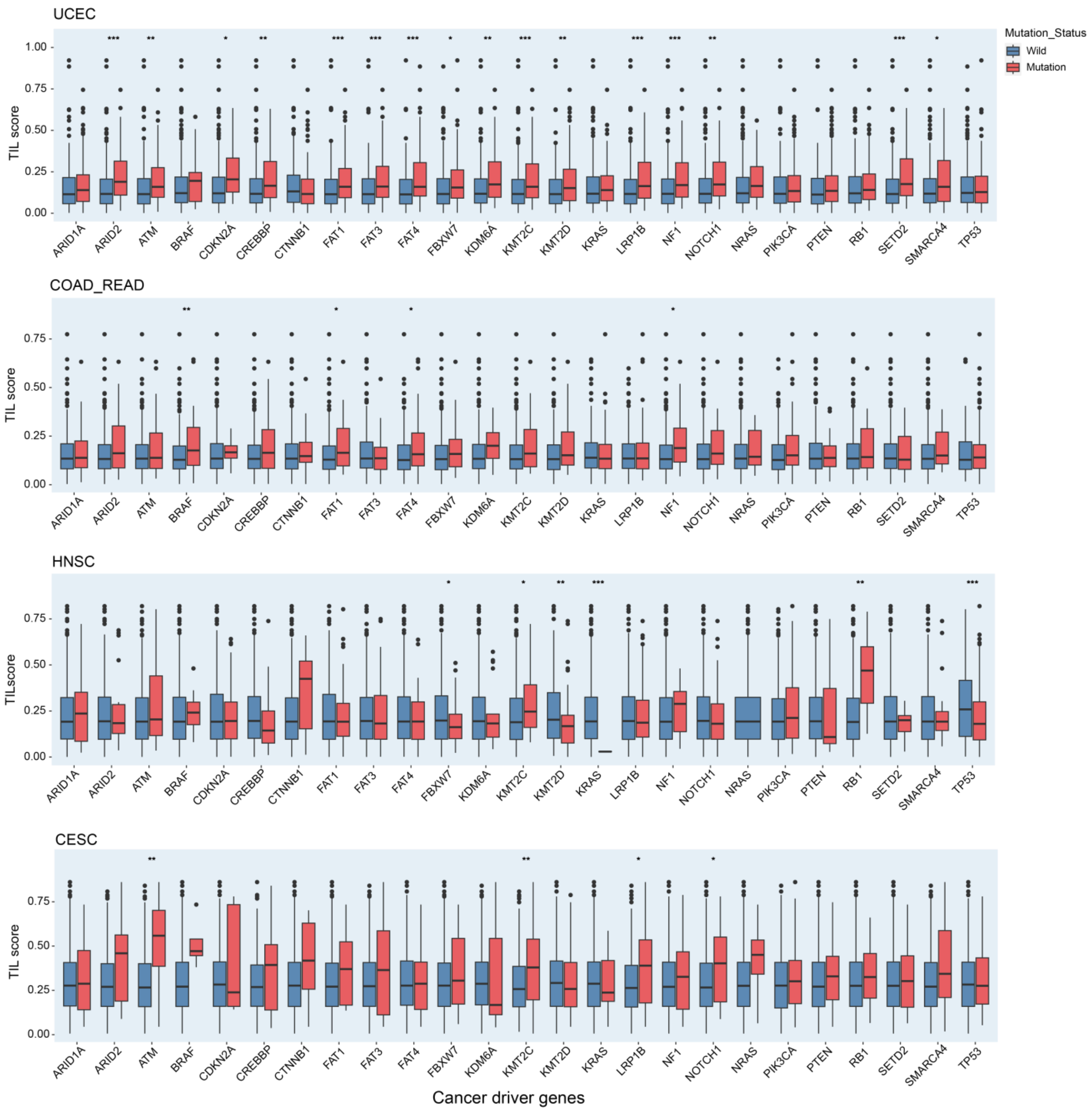

Supplementary Figure 10. Effects of SNVs for 25 genes on TIL scores in UCEC, COAD_READ, HNSC, and CESC. TIL scores between the two groups were compared by the t-test. “*”, P<0.05; “*”, P<0.01; “***”, P<0.001.

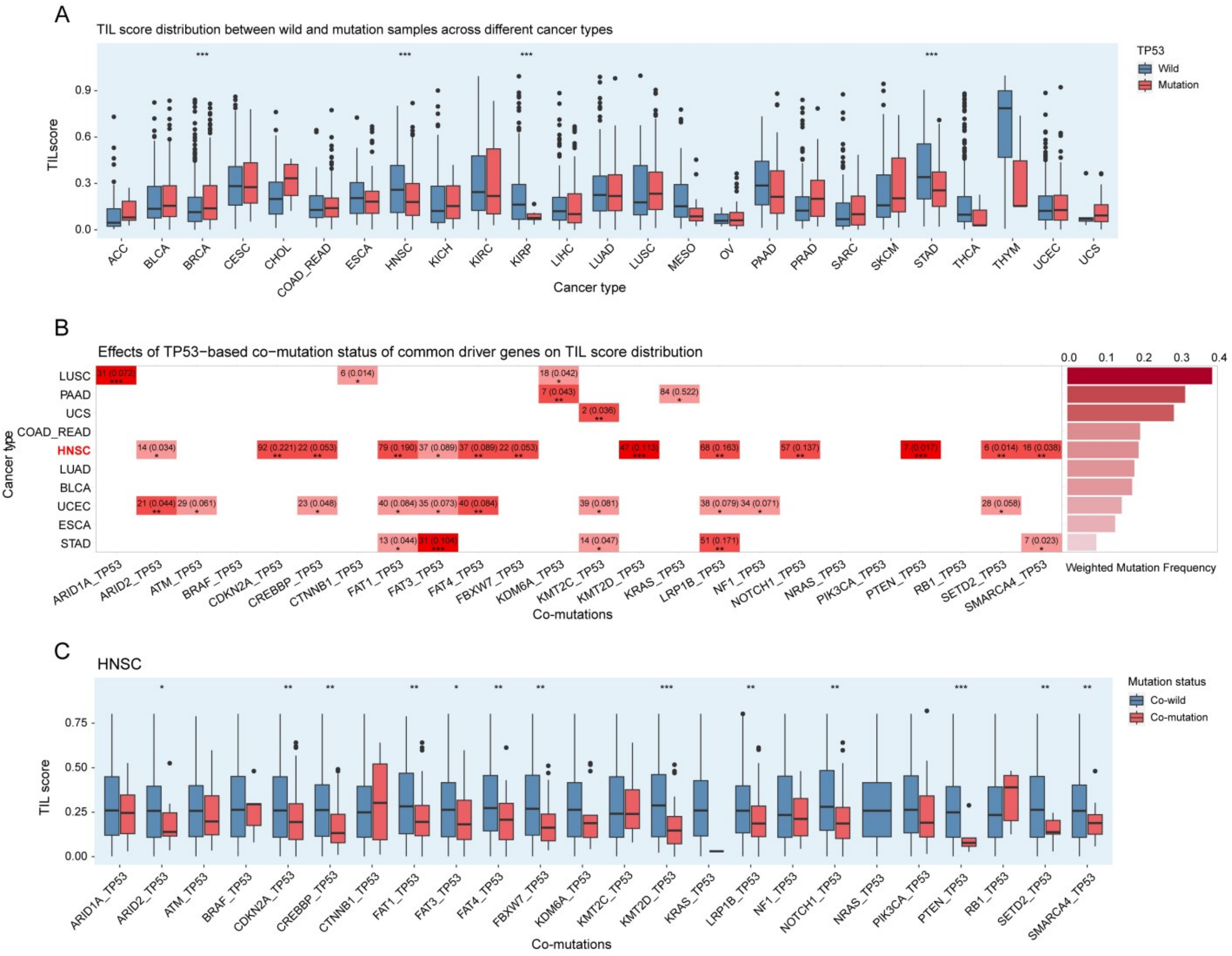

Supplementary Figure 11. Effects of TP53-based co-mutation on TIL score distributions across different cancer types. A, TIL score distribution differences between TP 53 wild and mutation samples across different cancer types. B, Effects of TP53-based co-mutation on TIL score distributions across different cancer types. C, Effects of TP-53 based co-mutation on TIL score distributions in HNSC. Color-filled squares indicate significant differences in TIL score distributions for two groups (t-test). Filled numbers represent the patient numbers and proportions of co-mutation for each gene in each cancer type. “*”, P<0.05; “**”, P<0.01; “***”, P<0.001.

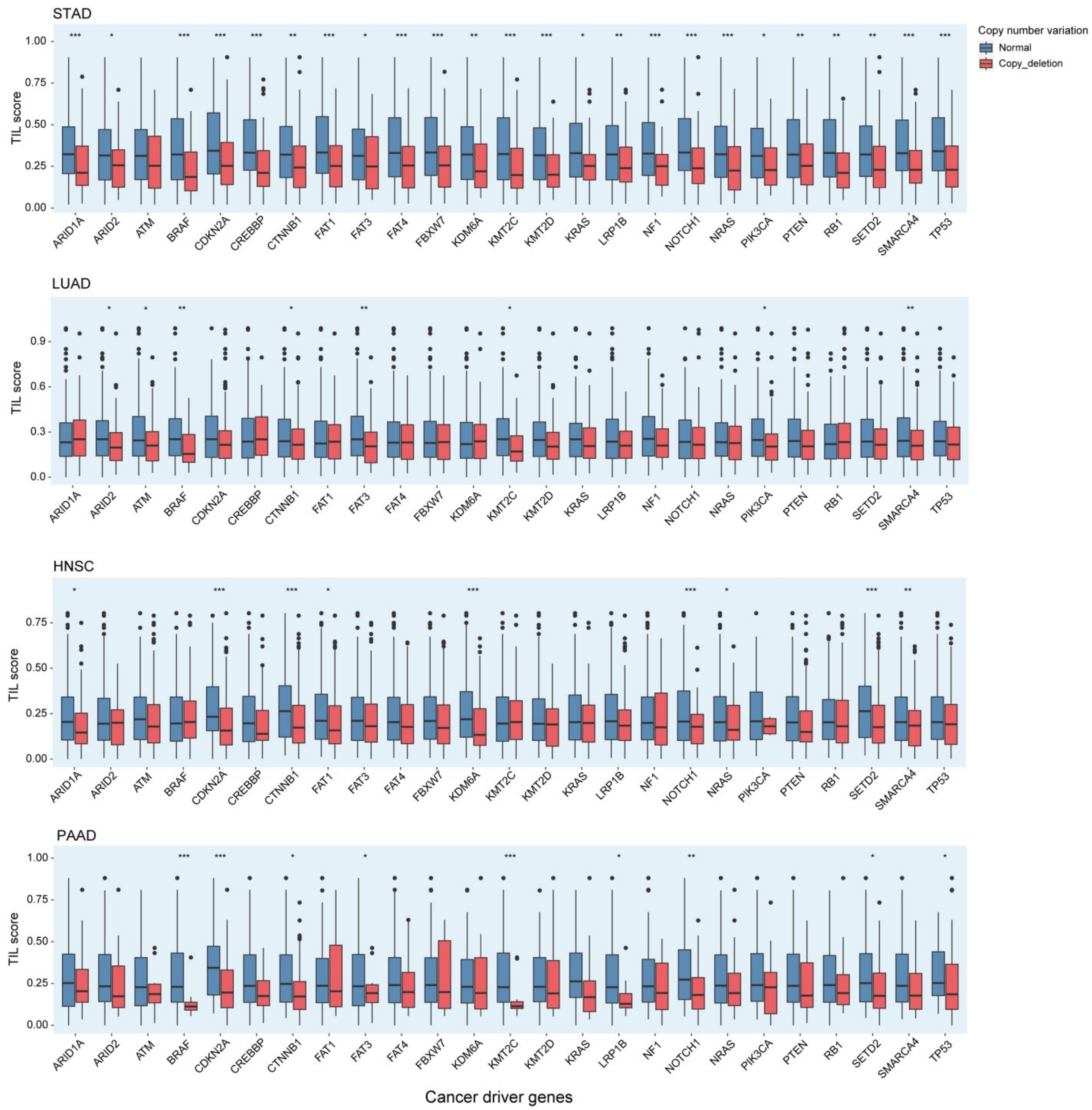

Supplementary Figure 12. Effects of copy deletion for 25 genes on TIL score distributions in STAD, LUAD, HNSC, and PAAD. TIL scores between the two groups were compared by the t-test. “*”, P<0.05; “*”, P<0.01; “***”, P<0.001.

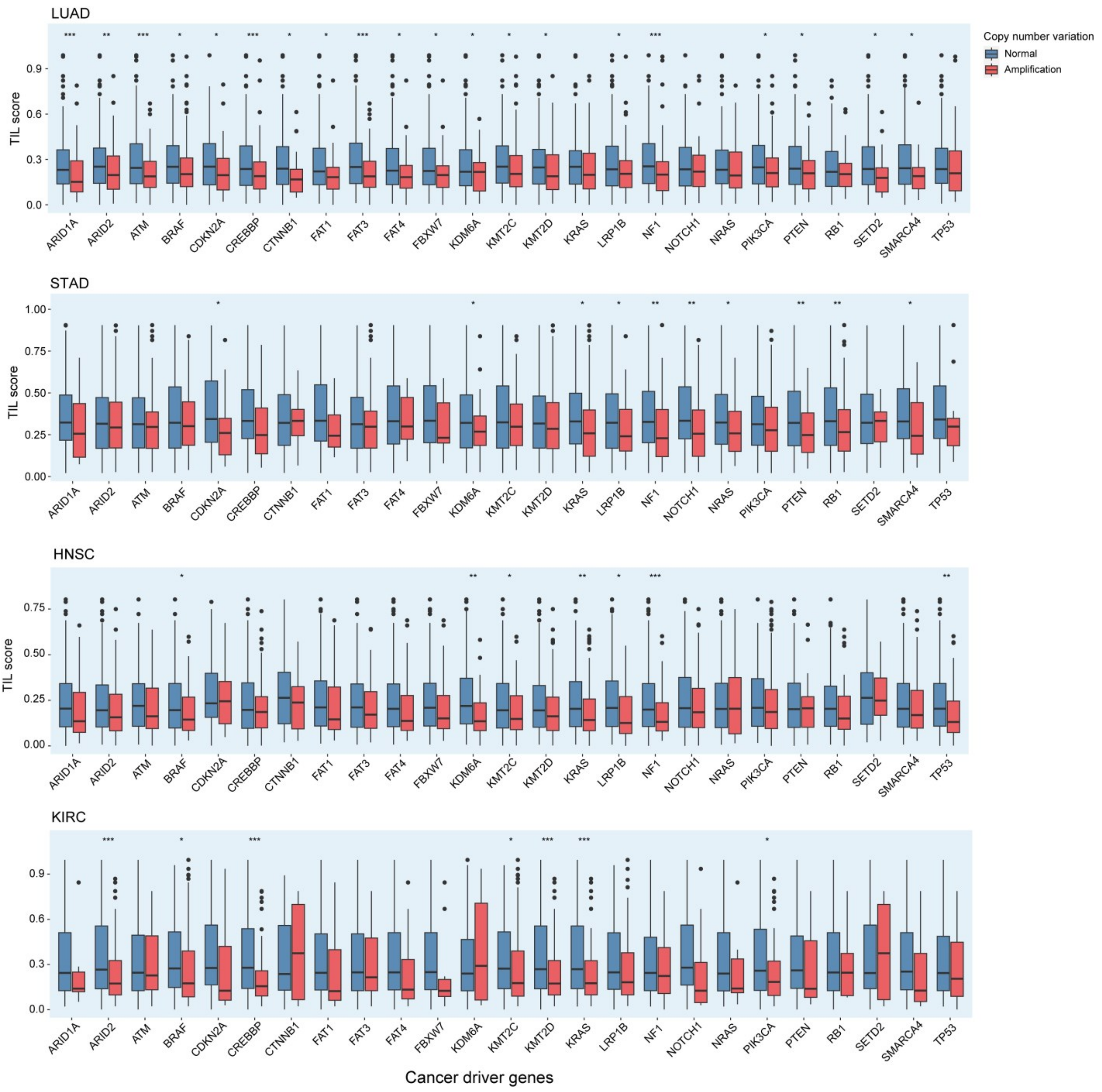

Supplementary Figure 13. Effects of copy amplification for 25 genes on TIL score distributions in LUAD, STAD, HNSC and KIRC. TIL scores between the two groups were compared by the t-test. “*”, P<0.05; “*”, P<0.01; “***”, P<0.001.

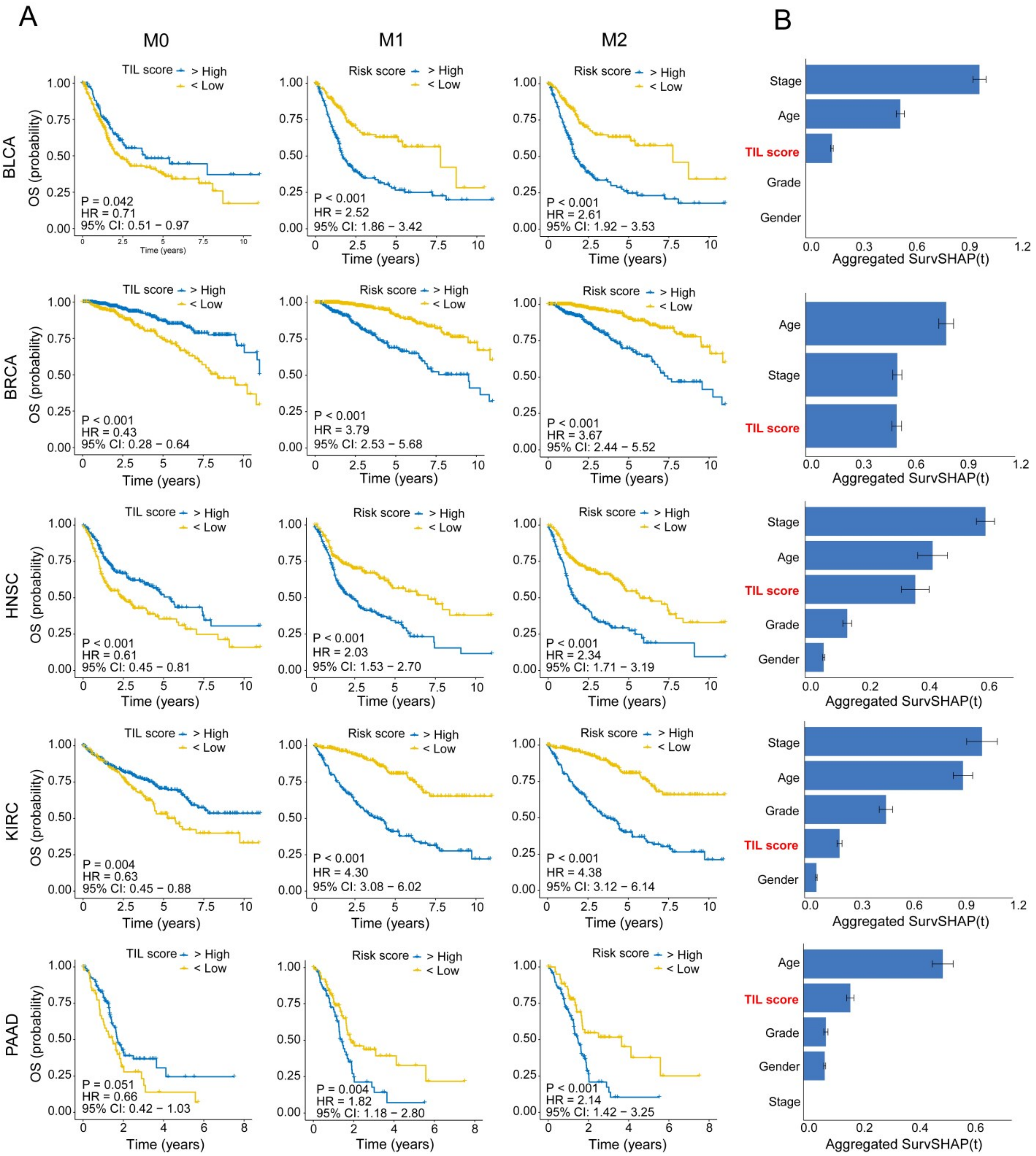

Supplementary Figure 14. Performance of different prognostic models for predicting OS in BLCA, BRCA, HNSC, KIRC and PAAD. M1, prognostic models based on clinical data only; M2, prognostic models established by clinical data combined with TIL scores. A, Kaplan-Meier curves of patient stratification for OS across different cancer types under different models. M0, Effects of TIL scores on OS. B, the corresponding average aggregated SurvSHAP(t) values of each variable for M2 models. SurvSHAP(t) is a kind of time-dependent explanations of machine learning survival models. An aggregated SurvSHAP(t) value of one variable represents its importance measure in one case. Average aggregated SurvSHAP(t) value of one variable represents its global importance across all samples in the model.

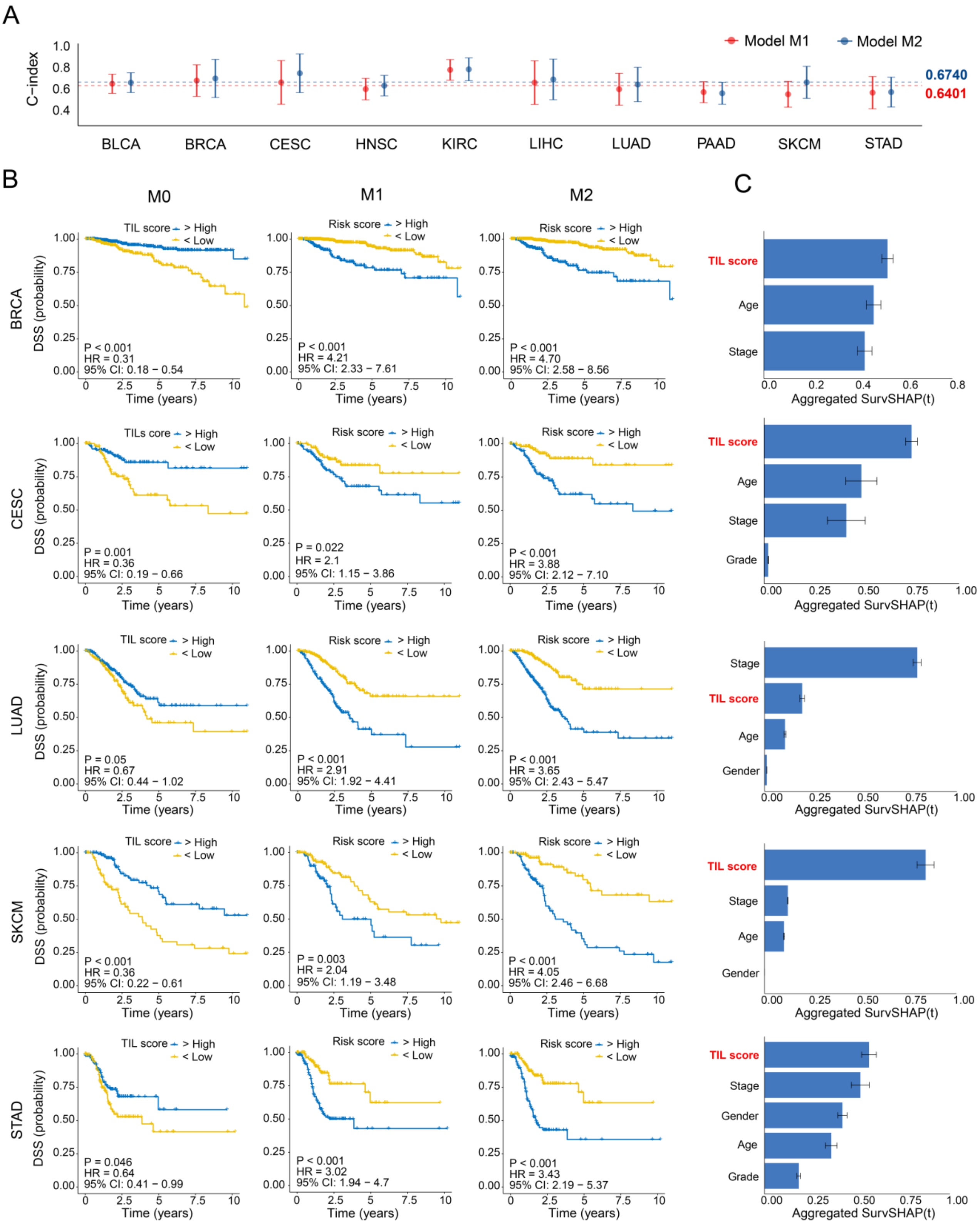

Supplementary Figure 15. Performance of different prognostic models for predicting DSS in BRCA, CESC, LUAD, SKCM and STAD. M1, prognostic models based on clinical data only; M2, prognostic models established by clinical data combined with TIL scores. A, C-indices of M1 and M2 in each cancer type in a 5-fold cross-validation. Horizontal lines indicate average C-indices across all cancer types for two types of models. B, Kaplan-Meier curves of patient stratification for DSS across different cancer types under different models. M0, Effects of TIL scores on DSS. C, the corresponding average aggregated SurvSHAP(t) values of each variable for M2 models. SurvSHAP(t) is a kind of time-dependent explanations of machine learning survival models. An aggregated SurvSHAP(t) value of one variable represents its importance measure in one case. Average aggregated SurvSHAP(t) value of one variable represents its global importance across all samples in the model.

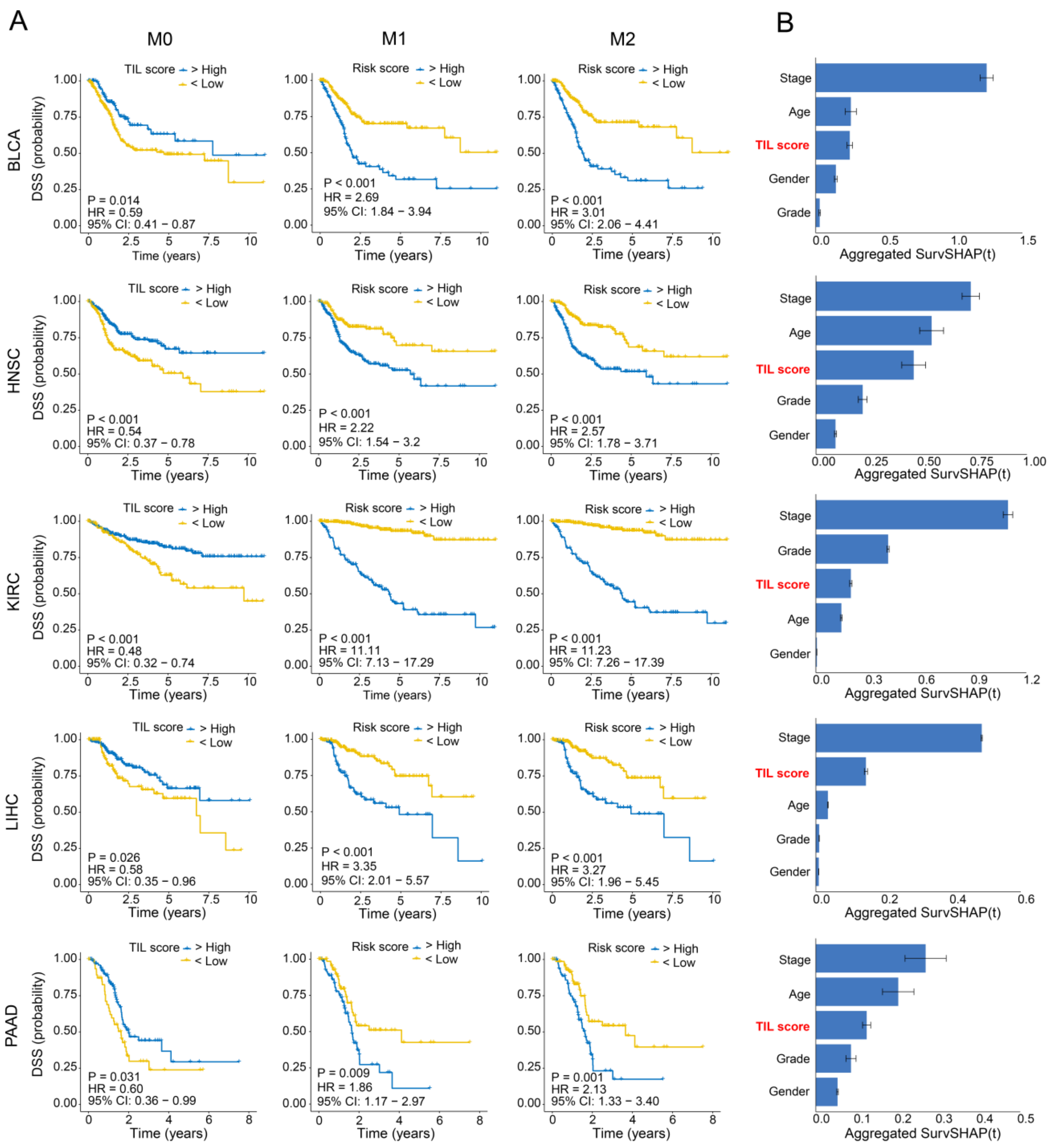

Supplementary Figure 16. Performance of different prognostic models for predicting DSS in BLCA, HNSC, KIRC, LIHC and PAAD. M1, prognostic models based on clinical data only; M2, prognostic models established by clinical data combined with TIL scores. A, Kaplan-Meier curves of patient stratification for DSS across different cancer types under different models. M0, Effects of TIL scores on DSS. B, the corresponding average aggregated SurvSHAP(t) values of each variable for M2 models. SurvSHAP(t) is a kind of time-dependent explanations of machine learning survival models. An aggregated SurvSHAP(t) value of one variable represents its importance measure in one case. Average aggregated SurvSHAP(t) value of one variable represents its global importance across all samples in the model.

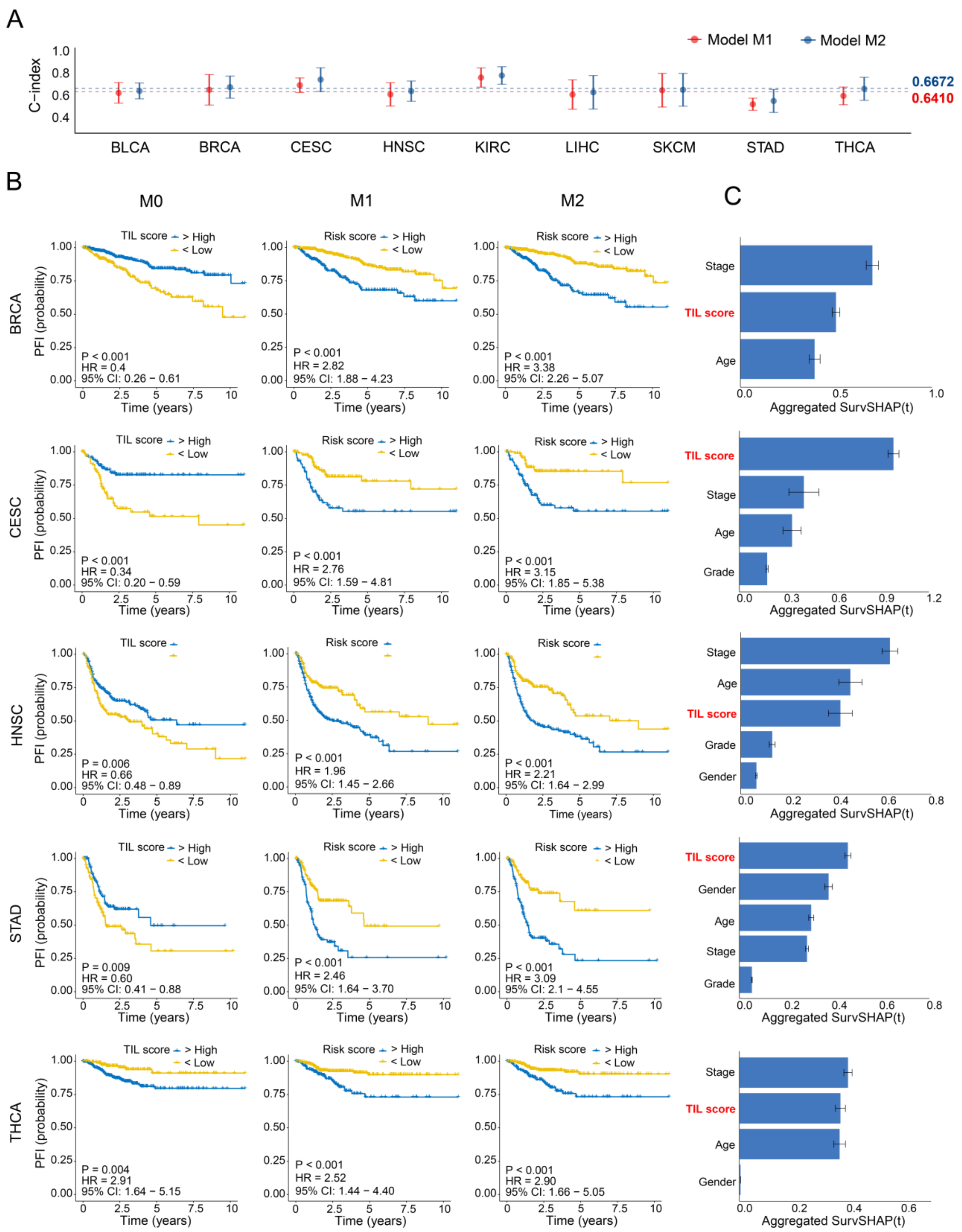

Supplementary Figure 17. Performance of different prognostic models for predicting PFI in BRCA, CESC, HNSC, STAD, and THCA. M1, prognostic models based on clinical data only; M2, prognostic models established by clinical data combined with TIL scores. A, C-indices of M1 and M2 in each cancer type in a 5-fold cross-validation. Horizontal lines indicate average C-indices across all cancer types for two types of models. B, Kaplan-Meier curves of patient stratification for PFI across different cancer types under different models. M0, Effects of TIL scores on PFI. C, the corresponding average aggregated SurvSHAP(t) values of each variable for M2 models. SurvSHAP(t) is a kind of time-dependent explanations of machine learning survival models. An aggregated SurvSHAP(t) value of one variable represents its importance measure in one case. Average aggregated SurvSHAP(t) value of one variable represents its global importance across all samples in the model.

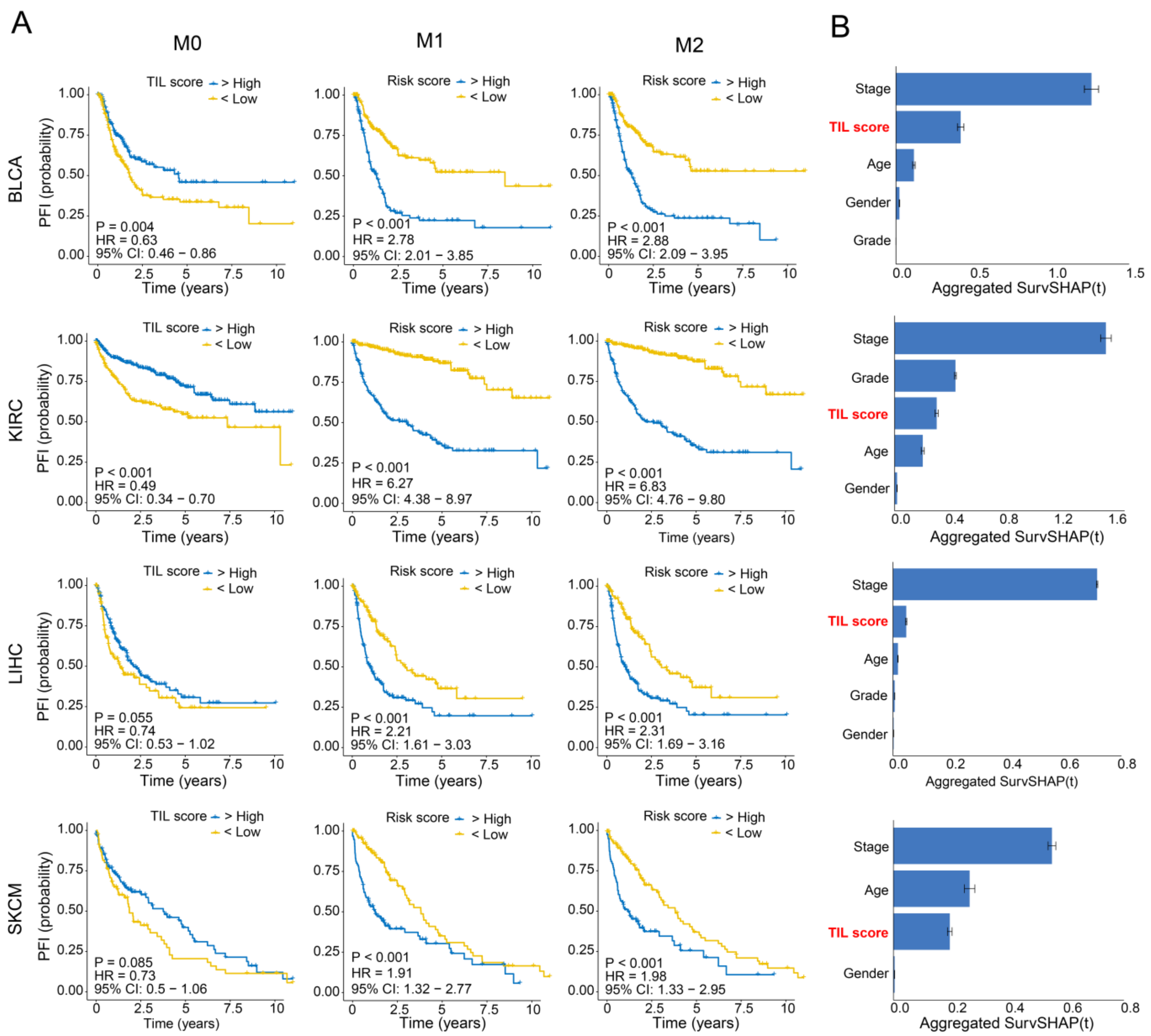

Supplementary Figure 18. Performance of different prognostic models for predicting PFI in BLCA, KIRC, LIHC and SKCM. M1, prognostic models based on clinical data only; M2, prognostic models established by clinical data combined with TIL scores. A, Kaplan-Meier curves of patient stratification for PFI across different cancer types under different models. M0, Effects of TIL scores on PFI. B, the corresponding average aggregated SurvSHAP(t) values of each variable for M2 models. SurvSHAP(t) is a kind of time-dependent explanations of machine learning survival models. An aggregated SurvSHAP(t) value of one variable represents its importance measure in one case. Average aggregated SurvSHAP(t) value of one variable represents its global importance across all samples in the model.

Supplementary Table 1. Distribution of patch counts across 28 cancer types in the initial dataset

Cancer types	Total number of WSIs in TCGA	Total number of WSIs after initial selection*	Total number of patches in selected WSIs	Patch labeling and selection for the dataset (N)				Number of WSIs from which the final patches were derived	Total patches of WSIs from which the final patches were derived
				TIL-positive	TIL-negative	Non-tumor/necrotic	Total		
BLCA	457	444	21,333,370	1,259	1,649	1,859	4,767	372	18,751,223
BRCA	1,133	930	31,113,409	1,188	1,784	1,748	4,720	129	5,035,683
CESC	279	278	8,421,554	1,334	1,748	1,782	4,864	98	2,740,200
COADREAD	624	324	10,428,565	986	1,815	1,802	4,603	96	3,306,599
HNSC	472	450	15,312,927	1,258	1,938	1,596	4,792	105	11,508,275
KIRC	519	478	19,935,295	1,155	1,785	1,908	4,848	306	4,930,175
KIRP	300	279	11,148,896	314	1,692	1,794	3,800	111	2,928,364
LIHC	379	359	14,705,603	1,380	1,938	1,825	5,143	73	2,900,678
LUAD	541	480	17,490,605	1,359	1,780	1,804	4,943	72	12,434,325
LUSC	512	477	17,593,127	1,214	1,823	1,767	4,804	302	1,805,488
PRAD	449	315	10,490,867	1,023	1,913	1,911	4,847	49	2,217,460
SARC	600	592	29,534,428	1,071	1,794	1,742	4,607	88	4,114,815
SKCM	475	439	17,422,828	1,539	1,802	1,800	5,141	87	4,115,791
STAD	442	321	11,454,111	1,153	1,814	1,795	4,762	89	3,942,150
THCA	519	508	20,550,236	1,260	1,798	1,797	4,855	100	3,845,582
UCEC	566	551	27,686,036	1,186	1,778	1,805	4,769	99	5,776,828
CAUM	538	496	22,089,650	1,107	1,805	1,816	4,728	112	4,484,539
PAPE	563	528	20,782,085	990	1,777	1,806	4,573	89	3,646,250
TTKO	661	639	28,990,855	1,372	1,753	1,797	4,922	110	4,054,016
**Total**	**10,029**	**8,888**	**356,484,447**	**22,148**	**34,186**	**34,154**	**90,488**	**2,487**	**102,538,441**

*, WSIs with bubbles, overlapping tissues, poor staining, and artificial markings were excluded from consideration.

CAUM, consisting of CHOL, ACC, UCS, and MESO. PAPE, consisting of PAAD, PCPG, and ESCA. TTKO, consisting of TGCT, THYM, KICH, and OV.

Supplementary Table 2. Distribution of patch counts across 3 cancer types in the independent test set

Cancer types	Total number of WSIs selected	Total number of patches in selected WSIs	Patch labeling and selection for the dataset (N)			
			TIL-positive	TIL-negative	Non-tumor/necrotic	Total
RUMC-BRCA	48	1,227,542	162	1,219	1,619	3,000
CPTAC-LUAD	47	1,118,817	224	1,357	1,419	3,000
CPTAC-LUSC	50	973,954	291	1,769	940	3,000
**Total**	**145**	**2,220,313**	**677**	**4,345**	**3,978**	**9,000**

Supplementary Table 3. Convolutional Autoencoder Model Architecture

Layer(type)	Output Shape	Param #
conv2d_616(Conv2D)	(None, 152, 152, 32)	896
max_pooling2d_15(MaxPooling2D)	(None, 76, 76, 32)	0
conv2d_617(Conv2D)	(None, 76, 76, 8)	2312
max_pooling2d_16(MaxPooling2D)	(None, 38, 38, 8)	0
conv2d_618(Conv2D)	(None, 38, 38, 8)	584
max_pooling2d_17(MaxPooling2D)	(None, 19, 19, 8)	0
conv2d_619 (Conv2D)	(None, 19, 19, 8)	584
up_sampling2d_3(UpSamplingg2D)	(None, 38, 38, 8)	0
conv2d_620(Conv2D)	(None, 38, 38, 8)	584
up_sampling2d_4(UpSampling2D)	(None, 76, 76, 8)	0
conv2d_621(Conv2D)	(None, 76, 76, 32)	2336
up_sampling2d_5(UpSampling2D)	(None, 152, 152, 32)	0
conv2d_622(Conv2D)	(None, 152, 152, 3)	8867

Total params: 8,163, Trainable params: 8,163

Supplementary Table 4. The number of selected genes at each step of the analysis (OS)

Cancer types	Immune-related DEGs	Stromal-related DEGs	Immune-related genes determined by WGCNA	Stromal-related genes determined by WGCNA	TME-related genes	Genes associated with survival by LASSO regression	Genes with significant (P<0.1) impact on survival (Cox univariate regression) used for establishing TME risk score
BLCA	2244	1857	1373	1072	619	6	6
BRCA	829	755	6844	4749	454	5	5
CESC	1515	885	8399	304	425	11	10
COADREAD	1185	1808	1649	1534	520	12	8
ESCA	1178	1372	302	801	374	10	7
HNSC	1049	1189	621	560	424	6	6
KIRC	617	374	276	2452	141	15	9
KIRP	893	1254	3241	426	161	8	5
LIHC	1172	1459	517	517	321	3	3
LUAD	699	773	1393	1452	183	15	13
LUSC	1508	1118	3846	8517	521	12	12
OV	1283	970	713	823	378	8	8
PAAD	1038	1502	534	1319	359	10	10
PRAD	1021	1381	3065	4135	526	-	-
SARC	2553	2628	7857	8588	1080	5	5
SKCM	1256	1423	5894	593	406	13	13
STAD	1289	1834	387	1425	672	24	13
THCA	1575	1077	9155	5415	484	-	-
UCEC	862	675	8793	4806	258	6	6

-, the number of cases where the outcome event (death) occurred by the recorded follow-up date was too small to select survival-related genes.

DEGs, Differentially expressed genes; WGCNA, Weighted gene co-expression network analysis.

Supplementary Table 5. TME risk score for predicting the OS

Cancer types	TMErisk score
BLCA	TMErisk=0.00016*Exp(FN1)+0.00463*Exp(CERCAM)-0.00407*Exp(CD3D)+0.00767*Exp(MAP1B)-0.00873* Exp(GNLY)+0.01375* Exp(HEYL)
BRCA	TMErisk=-0.00017* Exp(CD74)+0.00029* Exp(CD14)+0.00281* Exp(MMP13)-0.00592* Exp(IRF1)-0.00914* Exp(IL2RG)
CESC	TMErisk=0.00012*Exp(KRT81)-0.01511*Exp(TPPP3)-0.00473*Exp(LSP1)+0.00526*Exp(OLR1)-0.00957*Exp(FAM3B-0.03242*Exp(LAG3)+ 0.02072*Exp(SPRY4)+ 0.00294*Exp(JAK3)-0.03609*Exp(BIN2)-0.02158*Exp(S1PR4)
COADREAD	TMErisk=0.00107*Exp(GPX3)+0.00087*Exp(IDO1)-0.03295*Exp(CPA3)-0.02026*Exp(CCL11)+ 0.05012*Exp(HEYL)+ 0.01659*Exp(CAV2)+ 0.00530*Exp(UCHL1)+ 0.02939*Exp(CD37)
ESCA	TMErisk=-0.00684*Exp(MXRA8)+0.00693*Exp(GAS1)+0.02441*Exp(RGS16)+0.00659*Exp(WIPF1)-0.00281*Exp(MFAP5)-0.02881*Exp(TWIST1)-0.03811* Exp(GPC6)
HNSC	TMErisk=-0.00907*Exp(BATF)-0.01346*Exp(ATP2A3)+0.01892*Exp(FCGR2A)-0.00278*Exp(JAK3)-0.01038*Exp(CD5)-0.00494*Exp(CD27)
KIRC	TMErisk=-0.00557*Exp(APLNR)+0.00347*Exp(VSIG4)-0.02028*Exp(PPARGC1A)+0.00324*Exp(TNFSF13B)+0.01799*Exp(LIMD2)-0.00444*Exp(FMNL1)-0.00300*Exp(LAG3)-0.00840*Exp(FCGR1A)+0.00341*Exp(LILRB3)
KIRP	TMErisk=-0.01174*Exp(TREM2)+0.03478* Exp(TBXAS1)+0.03092* Exp(LIMD2)-0.01637* Exp(PDE6G)+0.07458* Exp(GNB4)
LIHC	TMErisk=0.00120* Exp(FCER1G)+0.00635* Exp(CSF1)-0.03463* Exp(CLEC3B)
LUAD	TMErisk=0.00002*Exp(HLA_DRA)-0.00005*Exp(CD74)-0.00012*Exp(HLA_DRB5)+0.00045*Exp(COL6A2)-0.00154*Exp(LCP1)+ 0.00021*Exp(SERPINE1)+ 0.00233*Exp(PSMB9)+0.00249*Exp(GBP1)-0.00440*Exp(HLA_DMB)+0.00468*Exp(AXL)-0.00506*Exp(MNDA)-0.00720*Exp(MS4A7)+0.00322* Exp(LOX)
LUSC	TMErisk=0.000082*Exp(HLA_B)+0.004049*Exp(CCL13)+0.002849*Exp(IL4I1)+0.002198*Exp(ACSL5)+0.003987*Exp(ALDH3B1)+0.006587*Exp(RND1)+0.008 855*Exp(IL36RN)+0.02400*Exp(DERL3)+0.000843*Exp(TGM2)+0.001222*Exp(C11orf96)-0.002347*Exp(CCDC69)+0.009483*Exp(PTGIS)
OV	TMErisk=0.00136*Exp(TGFBI)-0.00171*Exp(PSMB9)+0.00416*Exp(VSIG4)-0.00318*Exp(CXCL9)-0.00370*Exp(CXCL11)+0.00770*Exp(CCDC80)+0.00006*Exp(CILP2)-0.02623*Exp(SELL)
PAAD	TMErisk=-0.00016*Exp(FN1)+0.00275*Exp(CLIC4)-0.00527*Exp(ANXA6)+0.00340*Exp(LTBP1)+0.00121*Exp(INHBA)+0.01324*Exp(LOX)-0.00452*Exp(PIM2)-0.00582*Exp(FOXA2)-0.00069*Exp(NGFR)-0.03481*Exp(ATP8B2)
PRAD	-
SARC	TMErisk=0.00087*Exp(MARCKS)+0.00224*Exp(MYH10)-0.01294*Exp(SECTM1)+0.01238*Exp(TNFSF4)+0.00341*Exp(PRSS35)
SKCM	TMErisk=-0.00033*Exp(FCER1G)+0.00033*Exp(BCAN)-0.00009*Exp(HLA_DQB1)+0.00104*Exp(KIT)-0.00019*Exp(PIM2)-0.00201*Exp(GBP4)-0.00544*Exp(UBA7)-0.00168*Exp(MZB1)-0.00008*Exp(PARP12)-0.00235*Exp(CCL8)-0.00427*Exp(APOBEC3G)-0.00296*Exp(CLIC2)-0.01018*Exp(TLR2)
STAD	TMErisk=0.00560*Exp(AXL)+0.00409*Exp(MS4A4A)+0.00625*Exp(LY96)-0.00017*Exp(DUSP1)+0.00154*Exp(GPNMB)+0.00316*Exp(VWF)+0.00200*Exp(CXCR4)-0.00430*Exp(SDC2)+0.00235*Exp(RGS1)-0.00013*Exp(KIT)+0.00486*Exp(BASP1)-0.06861*Exp(PDCD1)-0.01298*Exp(FERMT3)
THCA	-
UCEC	TMErisk=0.00153*Exp(ELN)+0.00216*Exp(LRRN2)+0.00474*Exp(TMSB15A)-0.01609*Exp(BATF)+0.00107*Exp(FOXO6)+0.01243*Exp(SIX1)

TMErisk score= $\sum \beta_i$ *Exp(*i*); β_i, risk coefficient of gene *i*; Exp(*i*), Expression value of gene *i*.

-, the number of cases where the outcome event (death) occurred by the recorded follow-up date was too small to establish the TMErisk score.

Supplementary Table 6. The number of selected genes at each step of the analysis (DSS)

Cancer types	Immune-related DEGs	Stromal-related DEGs	Immune-related genes determined by WGCNA	Stromal-related genes determined by WGCNA	TME-related genes	Genes associated with survival by LASSO regression	Genes with significant (P<0.1) impact on survival (Cox univariate regression) used for establishing TME risk score
BLCA	1664	1857	1373	1072	573	27	16
BRCA	829	755	6844	4749	454	10	10
CESC	1498	885	8399	304	432	12	12
COADREAD	1155	1808	1649	1534	519	9	8
ESCA	1009	1219	302	801	375	5	5
HNSC	1061	1251	621	560	416	10	10
KIRC	617	368	276	2452	140	18	11
KIRP	819	1177	3241	426	159	9	9
LIHC	1164	2453	517	517	439	1	1
LUAD	853	773	1393	1452	197	14	10
LUSC	1214	1118	3846	8517	455	3	3
OV	1309	1159	713	823	351	11	11
PAAD	1059	1509	534	1319	373	12	7
PRAD	897	1388	3065	4135	503	-	-
SARC	2608	2628	7857	8588	1086	5	5
SKCM	1336	1485	5894	593	460	18	14
STAD	1255	1881	387	1425	663	6	6

-, the number of cases where the outcome event (death) occurred by the recorded follow-up date was too small to select survival-related genes.
DEGs, differentially expressed genes; WGCNA, weighted gene co-expression network analysis.

Supplementary Table 7. TME risk score for predicting DSS

Cancer types	TMErisk score
BLCA	TMErisk=0.00028*Exp(FN1)-0.00084*Exp(HLA_DMA)-0.00164*Exp(IL32)+0.00212*Exp(CERCAM)-0.00517*Exp(RGS1)-0.00091*Exp(GBP4)+ 0.00236*Exp(STC1)-0.03849*Exp(IL3RA)+0.00189*Exp(MAP1B)-0.01227*Exp(GNLY)+0.02770*Exp(HEYL)+0.00400*Exp(CSF2RB)- 0.00891*Exp(TMC8)-0.02753*Exp(TMC7)+0.01574*Exp(SLC9A9)-0.04968*Exp(PLEKHH1)
BRCA	TMErisk=-0.00014*Exp(CD74)-0.00069*Exp(STC2)-0.00171*Exp(C3)+0.00105*Exp(EEF1A2)-0.00763*Exp(ELOVL2)-0.00611*Exp(APOBEC3C)+ 0.00247*Exp(MMP13)-0.00774*Exp(APOL3)+0.00227*Exp(NR2F1)+0.02463*Exp(PCDH18)
CESC	TMErisk=0.00077*Exp(CXCL8)-0.02580*Exp(TPPP3)-0.00799*Exp(LSP1)+0.00522*Exp(OLR1)-0.03629*Exp(LAG3)+0.00056*Exp(SCX)+ 0.02509*Exp(SPRY4)-0.01349*Exp(FGL2)+0.03586*Exp(JAK3)-0.03717*Exp(PILRA)-0.01890*Exp(BIN2)-0.05171*Exp(S1PR4)
COADREAD	TMErisk=0.00198*Exp(IGFBP6)-0.04116*Exp(CCL11)+0.00154*Exp(CX3CL1)+0.01281*Exp(CAV2)+0.03236*Exp(ENPP2)-0.00049*Exp(CALB2)+ 0.01611* Exp(UCHL1)+0.02268* Exp(CD37)
ESCA	TMErisk=0.00211* Exp(RGS2)+0.01153* Exp(GAS1)+0.00428* Exp(RGS16)+0.01302* Exp(PRSS23)+0.01229* Exp(GPR34)
HNSC	TMErisk=0.00089*Exp(FHL1)+0.000004*Exp(PIM2)+0.00337*Exp(BATF)-0.01006*Exp(CMPK2)+ 0.01554*Exp(NRP1)+ 0.01498*Exp(FCGR2A)+ 0.01389*Exp(VSIG4)-0.04944* Exp(GALM)-0.03507* Exp(CYTIP)-0.03603* Exp(CCR7)
KIRC	TMERisk=0.00188* Exp(FCER1G)-0.00524* Exp(COL15A1)-0.03422* Exp(PPARGC1A)+0.00836* Exp(TNFSF13B)+0.00475* Exp(LIMD2)+0.01787* Exp(FMNL1)-0.00116* Exp(BATF)+0.01930* Exp(MILR1)-0.08322* Exp(SIGLEC8)+0.01498* Exp(TRPM2)-0.00554* Exp(LILRB3)
KIRP	TMErisk=0.01948*Exp(TBXAS1)-0.07482*Exp(TLR2)+0.03275*Exp(LIMD2)+0.10984*Exp(ADORA3)+0.08566*Exp(TRPV2)-0.34634*Exp(SIGLEC8)- 0.09125* Exp(CD300LF)-0.10120* Exp(PRAM1)+0.16481* Exp(GNB4)
LIHC	TMErisk=-0.07184* Exp(CLEC3B)
LUAD	TMErisk=-0.00022*Exp(HLA_DRB5)-0.00125*Exp(LCP1)+0.00030*Exp(SERPINE1)-0.00259*Exp(DCN)+0.00422*Exp(GBP1)-0.00444*Exp(PRELP)+ 0.00521*Exp(AXL)+0.01338* Exp(LOX)-0.00697* Exp(CLEC7A)-0.01724* Exp(PILRA)
LUSC	TMErisk=0.00160* Exp(TGM2)+0.00053* Exp(MMP9)+0.02040* Exp(VEGFC)
OV	TMErisk=0.00049*Exp(TGFBI)-0.00149*Exp(TAP1)+0.00150*Exp(VSIG4)-0.00929*Exp(CXCL11)+0.00693*Exp(CCDC80)-0.00163*Exp(C5AR1)+ 0.00756*Exp(PODNL1)+0.02058* Exp(MS4A7)+0.00782* Exp(MILR1)-0.00971* Exp(IL2RG)-0.03008* Exp(SELL)
PAAD	TMErisk=0.00025*Exp(FN1)+0.00031*Exp(SERPINE1)+0.00200*Exp(RGS16)-0.00060*Exp(INHBA)+0.00063*Exp(LOX)-0.00373*Exp(FOXA2)+ 0.00278*Exp(MDFIC)
PRAD	-
SARC	TMErisk=0.00109* Exp(ALPL)+0.00100* Exp(LOX)+0.00075* Exp(DRAM1)-0.02212* Exp(SECTM1)+0.01243* Exp(TNFSF4)
SKCM	TMErisk=-0.00024*Exp(FCER1G)+0.00041*Exp(BCAN)+0.00015*Exp(PSMB8)+0.00019*Exp(APOL1)-0.00174*Exp(GBP4)-0.0052*Exp(UBA7)- 0.0019*Exp(MZB1)-0.00364*Exp(CD40)-0.00505*Exp(BATF2)-0.00464*Exp(KIAA0040)-0.00234*Exp(C2)-0.00343*Exp(CCL8)-0.00177*Exp(CLIC2)- 0.00881*Exp(TLR2)
STAD	TMErisk=0.00120* Exp(CXCR4)+0.01129* Exp(GLIS2)+0.00792* Exp(SSPN)+0.00700* Exp(SELP)+0.00698* Exp(RASSF8)+0.00530* Exp(C4B)
THCA	-

UCEC	TMErisk=0.00076*Exp(APOL1)+0.00008*Exp(SST)+0.00455*Exp(COL6A3)+0.00129*Exp(FAM107A)+0.00580*Exp(CD52)+0.00197*Exp(ELN)+0.00089*Exp(LRRN2)-0.02382*Exp(LRP4)+0.00966*Exp(SERPINE1)-0.00420*Exp(BATF)-0.02356*Exp(CD79A)-0.01184*Exp(LSP1)+0.00549* Exp(FOXO6)+0.00804* Exp(SIX1)-0.06629* Exp(APOBR)

TMErisk score= $\sum \beta_i$ *Exp(i); β_i, risk coefficient of gene i; Exp(i), Expression value of gene i.

-, the number of cases where the outcome event (death) occurred by the recorded follow-up date was too small to establish the TMErisk score.

Supplementary Table 8. The number of selected genes at each step of the analysis (PFI)

Cancer types	Immune-related DEGs	Stromal-related DEGs	Immune-related genes determined by WGCNA	Stromal-related genes determined by WGCNA	TME-related genes	Genes associated with survival by LASSO regression	Genes with significant (P<0.1) impact on survival (Cox univariate regression) used for establishing TME risk score
BLCA	1668	1860	1373	1072	573	20	16
BRCA	838	943	6844	4749	506	19	17
CESC	1546	885	8399	304	439	10	10
COADREAD	1188	1837	1649	1534	522	9	9
ESCA	1007	1234	302	801	389	-	-
HNSC	1067	1170	621	560	420	12	9
KIRC	623	374	276	2452	129	26	16
KIRP	842	1126	3241	426	157	13	9
LIHC	1177	1313	517	517	305	1	1
LUAD	865	775	1393	1452	198	11	10
LUSC	1214	1118	3846	8517	455	14	10
OV	1309	1080	713	823	371	11	10
PAAD	955	1511	534	1319	377	12	6
PRAD	884	1230	3065	4135	445	7	7
SARC	2539	2751	7857	8588	1134	20	18
SKCM	1192	1440	5894	593	402	8	8
STAD	1346	1902	387	1425	665	20	10
THCA	1477	1058	9155	5415	440	16	13
UCEC	969	671	8793	4806	270	10	10

-, the number of cases where the outcome event (disease progression) occurred by the recorded follow-up date was too small to select survival-related genes.
DEGs, differentially expressed genes; WGCNA, weighted gene co-expression network analysis.

Supplementary Table 9. TMErisk score for predicting PFI

Cancer types	TMErisk score
BLCA	TMErisk=0.00027*Exp(FN1)+0.00120*Exp(CERCAM)-0.00646*Exp(RGS1)-0.00365*Exp(GBP4)+ 0.00414*Exp(LTBP2)+ 0.00226*Exp(STC1)+ 0.00235*Exp(CD3D)-0.00385*Exp(LOXL2)-0.01082*Exp(ODF3B)-0.00377*Exp(HLA_DMB)-0.03204*Exp(IL3RA)-0.00227*Exp(CD7)+ 0.01876*Exp(HEYL)-0.02776* Exp(TMC7)+0.02300* Exp(GNB4)+0.03216* Exp(ARHGAP31)
BRCA	TMErisk=-0.00007*Exp(CD74)-0.00050*Exp(STC2)+0.00355*Exp(GPX3)-0.00069*Exp(ESR1)-0.00067*Exp(HLA_DPB1)+ 0.00097*Exp(EEF1A2)+ 0.00109*Exp(RNASE6)-0.0035*Exp(ELOVL2)-0.00724*Exp(PSMB9)+0.00162*Exp(MMP13)-0.02003*Exp(ENPP2)-0.00222*Exp(CST7)+0.00601* Exp(RF1)+0.00597* Exp(TFCP2L1)+0.00049* Exp(VMO1)+0.00882* Exp(GPC6)-0.01702* Exp(LGALS2)
CESC	TMErisk=0.00156*Exp(TSPAN13)+0.00414*Exp(PCOLCE)-0.00881*Exp(LAG3)+ 0.01023*Exp(SORL1)+ 0.02163*Exp(TNFRSF10D)+ 0.00423*Exp(MLLT11)+0.00747* Exp(JAK3)-0.03623* Exp(IKZF3)-0.01411* Exp(BIN2)-0.06712* Exp(S1PR4)
COADREAD	TMErisk=0.00090*Exp(CXCL5)+0.00122*Exp(CAV1)+0.00512*Exp(NKD1)-0.06277*Exp(FGL2)+ 0.02730*Exp(HEYL)+ 0.01386*Exp(VIP)+ 0.00087*Exp(CAV2)+0.00985* Exp(CALB2)+0.01587* Exp(UCHL1)
ESCA	-
HNSC	TMErisk=0.00010*Exp(FN1)+0.00209*Exp(COX7A1)-0.01219*Exp(COL8A2)-0.01359*Exp(RGS5)-0.01361*Exp(CMPK2)-0.00644*Exp(ATP2A3)+ 0.03697* Exp(NRP1)-0.01262* Exp(CYTIP)-0.00165* Exp(JAK3)
KIRC	TMErisk=0.00056*Exp(FCER1G)-0.00213*Exp(COL15A1)+0.00040*Exp(VSIG4)+0.00413*Exp(RAC2)-0.00361*Exp(KIT)-0.01039*Exp(PPARGC1A)-0.00285*Exp(LAIR1)+ 0.01106*Exp(TNFSF13B)+ 0.00238*Exp(LIMD2)+ 0.04900*Exp(FMNL1)+ 0.00925*Exp(BATF)-0.10535*Exp(SPN)-0.00359* Exp(MILR1)+0.00110* Exp(CD72)+0.00265* Exp(FCGR1A)-0.00496* Exp(LILRB3)
KIRP	TMErisk=0.00733*Exp(RGS10)+0.03928*Exp(TBXAS1)+0.04831*Exp(LHFPL2)+0.01960*Exp(LIMD2)-0.10125*Exp(OSCAR)-0.04344*Exp(KCNJ5)-0.06031* Exp(SIGLEC8)+0.02796* Exp(CSF2RA)+0.02828* Exp(GNB4)
LIHC	TMErisk=-0.04613* Exp(CLEC3B)
LUAD	TMErisk=-0.00001*Exp(CD74)-0.00012*Exp(HLA_DRB5)-0.00023*Exp(HLA_DQB1)+0.00008*Exp(HLA_DQA1)-0.00157*Exp(LCP1)+ 0.00047*Exp(SERPINE1)+0.00436* Exp(GBP1)-0.00510* Exp(PRELP)+0.009479* Exp(LOX)+0.00913* Exp(CLEC7A)-0.01067* Exp(PILRA)
LUSC	TMErisk=-0.00008*Exp(NTS)-0.00034*Exp(GSTA1)+0.00196*Exp(TGM2)+0.00039*Exp(MMP9)-0.00118*Exp(MMP13)+ 0.00060*Exp(MARCO)-0.00277* Exp(CD79A)+0.00398* Exp(BARX1)+0.00162* Exp(SBK1)+0.01770*Exp(VEGFC)
OV	TMErisk=0.00132*Exp(NBL1)+0.000448*Exp(TGFBI)-0.00321*Exp(GPNMB)-0.00212*Exp(TAP1)+0.00471*Exp(VSIG4)-0.00083*Exp(CXCL9)+ 0.00186* Exp(RGS2)+0.01134* Exp(STAB1)+0.00881* Exp(LRRN4)-0.04908* Exp(IRF8)
PAAD	TMErisk=9.72E-05* Exp(FN1)+0.00052* Exp(SERPINE1)-0.00011* Exp(LOX)-0.00658* Exp(FOXA2)-0.01339* Exp(FHL1)+0.00919* Exp(FRMD6)
PRAD	TMErisk=0.00134*Exp(SPOCK3)+0.01845*Exp(FAM83D)*0.00874*Exp(TREM2)-0.05839*Exp(PCDH18)+0.04124*Exp(ARHGAP4)-0.06193*Exp(FAIM2)+0.06243* Exp(FOXS1)
SARC	TMErisk=-0.00017*Exp(DES)+0.00114*Exp(ENO2)+0.00242*Exp(LOX)+0.00142*Exp(PDPN)+0.00139*Exp(LTBP2)-0.01127* Exp(SIX2)-0.02322*Exp(CD40)-0.01150*Exp(OCIAD2)+ 0.00324*Exp(HEY2)+0.02726*Exp(ARHGAP33)+0.00433*Exp(TNFSF4)-0.00398*Exp(SERTAD4)+ 0.00447*Exp(ADAMTS12)+0.02554* Exp(TNFSF13B)+0.01721* Exp(ZNF700)-0.01348* Exp(PTGER3)-0.03261* Exp(HOXD4)-0.00845* Exp(CDON)

SKCM	TMErisk=0.00026*Exp(BCAN)-0.00080*Exp(GBP4)-0.00399*Exp(UBA7)-0.00103*Exp(MARCO)-0.00263*Exp(PARP12)-0.00632*Exp(LMO2)-0.00668* Exp(ABCG1)-0.00049* Exp(TNFSF13B)
STAD	TMErisk=0.00036*Exp(CCL21)+0.00027*Exp(NNMT)-0.00021*Exp(CCL19)+ 0.00120*Exp(AXL)+ 0.00216*Exp(SELL)+ 0.01215*Exp(GLIS2)+ 0.00251*Exp(PCDH7)+0.00435* Exp(FOXS1)+0.00842* Exp(SSPN)+0.00199* Exp(RASSF8)
THCA	TMErisk=0.00003*Exp(FN1)+0.00007*Exp(S100A4)-0.01458*Exp(APOD)+ 0.00454*Exp(MMP9)+ 0.00359*Exp(PASK)+ 0.00370*Exp(MUC21)-0.04602*Exp(SRPX)+0.01539*Exp(SCGB2A1)+0.02050*Exp(TGFB3)-0.04294*Exp(CPXM2)-0.07382*Exp(ABCG2)+ 0.02140*Exp(C2CD4A)+ 0.01114*Exp(TREM1)
UCEC	TMErisk=0.00004*Exp(SST)+0.00097*Exp(HMOX1)-0.00366*Exp(CST7)+0.00578*Exp(EFEMP1)+0.00276*Exp(KIF1A)-0.00213*Exp(LSP1)-0.02138*Exp(GYPC)+0.00588* Exp(FOXO6)+0.01521* Exp(SEMA6A)-0.02420* Exp(APOBR)

TMErisk score= $\sum \beta_i$ *Exp(i); β_i, risk coefficient of gene i; Exp(i), Expression value of gene i.

-, the number of cases where the outcome event (disease progression) occurred by the recorded follow-up date was too small to establish the TMErisk score.

Supplementary Table 10. Performance comparison for model selection

Model		Accuracy	Kappa	AUC				Precision				Recall				Specificity				F1 score			
				**A**	P	N	O	**A**	P	N	O	**A**	P	N	O	**A**	P	N	O	**A**	P	N	O
InceptionResNetV2	T	0.9878	0.9814	0.9996	0.9996	0.9995	0.9997	0.9879	0.9896	0.9844	0.9902	0.9878	0.9817	0.9898	0.9899	0.9933	0.9967	0.9905	0.9941	0.9878	0.9856	0.9871	0.9901
	V	**0.9838**	**0.9753**	0.9989	0.9994	0.9986	0.9990	0.9838	0.9891	0.9794	0.9849	0.9838	0.9797	0.9850	0.9854	0.9909	0.9965	0.9874	0.9909	0.9838	0.9844	0.9822	0.9852
VGG16	T	0.9799	0.9693	0.9988	0.9995	0.9985	0.9986	0.9799	0.9870	0.9745	0.9807	0.9799	0.9839	0.9797	0.9775	0.9887	0.9958	0.9844	0.9883	0.9799	0.9854	0.9771	0.9791
	V	0.9688	0.9523	0.9971	0.9987	0.9963	0.9968	0.9688	0.9793	0.9615	0.9693	0.9688	0.9714	0.9661	0.9697	0.9825	0.9934	0.9765	0.9814	0.9688	0.9753	0.9638	0.9695
VGG19	T	0.9730	0.9587	0.9979	0.9991	0.9974	0.9977	0.9730	0.9747	0.9703	0.9745	0.9730	0.9813	0.9695	0.9710	0.9854	0.9917	0.9820	0.9846	0.9730	0.9779	0.9699	0.9728
	V	0.9587	0.9369	0.9954	0.9980	0.9941	0.9951	0.9587	0.9643	0.9529	0.9608	0.9587	0.9691	0.9538	0.9567	0.9774	0.9884	0.9714	0.9763	0.9587	0.9667	0.9533	0.9588
ResNet34	T	0.9725	0.9579	0.9976	0.9979	0.9973	0.9978	0.9729	0.9829	0.9526	0.9867	0.9725	0.9590	0.9852	0.9685	0.9845	0.9946	0.9703	0.9921	0.9725	0.9708	0.9686	09775
	V	0.9617	0.9414	0.9959	0.9965	0.9952	0.9962	0.9621	0.9722	0.9416	0.9762	0.9617	0.9452	0.9753	0.9588	0.9786	0.9912	0.9633	0.9858	0.9617	0.9585	0.9582	0.9674
ResNet50	T	0.9842	0.9759	0.9993	0.9995	0.9991	0.9994	0.9843	0.9846	0.9810	0.9873	0.9842	0.9833	0.9840	0.9851	0.9915	0.9950	0.9884	0.9923	0.9842	0.9839	0.9825	0.9862
	V	0.9799	0.9693	0.9987	0.9992	0.9982	0.9988	0.9799	0.9810	0.9764	0.9827	0.9799	0.9790	0.9795	0.9808	0.9891	0.9939	0.9856	0.9895	0.9799	0.9800	0.9780	0.9818
InceptionV3	T	0.9783	0.9670	0.9987	0.9989	0.9982	0.9991	0.9784	0.9722	0.9745	0.9863	0.9784	0.9763	0.9770	0.9811	0.9888	0.9910	0.9845	0.9917	0.9784	0.9743	0.9758	0.9837
	V	0.9645	0.9458	0.9963	0.9969	0.9950	0.9972	0.9645	0.9570	0.9601	0.9739	0.9645	0.9633	0.9603	0.9696	0.9815	0.9860	0.9758	0.9842	0.9645	0.9601	0.9602	0.9717
Xception	T	0.9765	0.9640	0.9986	0.9987	0.9980	0.9990	0.9766	0.9729	0.9656	0.9901	0.9765	0.9725	0.9813	0.9741	0.9876	0.9912	0.9787	0.9941	0.9765	0.9727	0.9734	0.9820
	V	0.9687	0.9522	0.9971	0.9979	0.9961	0.9975	0.9688	0.9656	0.9578	0.9820	0.9687	0.9682	0.9712	0.9665	0.9834	0.9888	0.9740	0.9893	0.9687	0.9670	0.9645	0.9742
UNI	T	0.9843	0.9761	0.9994	0.9996	0.9993	0.9994	0.9845	0.9889	0.9712	0.9950	0.9843	0.9868	0.9945	0.9726	0.9913	0.9964	0.9821	0.9970	0.9843	0.9878	0.9827	0.9837
	V	0.9836	0.9750	0.9994	0.9997	0.9993	0.9994	0.9838	0.9874	0.9717	0.9936	0.9836	0.9858	0.9934	0.9724	0.9909	0.9959	0.9824	0.9962	0.9836	0.9866	0.9824	0.9829
CAE-based clustering	T	0.5021	0.2643	—	—	—	—	0.6251	0.2348	0.5525	0.9509	0.4893	0.5021	0.5706	0.5044	0.7864	0.5850	0.7194	0.9842	0.5328	0.2939	0.5614	0.6591
	V	0.5096	0.2753	—	—	—	—	0.6286	0.2425	0.5576	0.9501	0.4973	0.5096	0.5689	0.5183	0.7899	0.5900	0.7261	0.9835	0.5402	0.3033	0.5632	0.6707
InceptionResNetV2 (fold 9)	T_{imi}	**0.9853**	**0.9776**	**0.9995**	0.9996	0.9994	0.9996	**0.9854**	0.9854	0.9752	0.9950	**0.9853**	0.9851	0.9920	0.9791	**0.9822**	0.9951	0.9856	0.9969	**0.9853**	0.9853	0.9836	0.9870
	V_{imi}	**0.9787**	**0.9669**	**0.9988**	0.9992	0.9983	0.9991	**0.9788**	0.9671	0.9784	0.9868	**0.9787**	0.9836	0.9788	0.9754	**0.9705**	0.9905	0.9832	0.9933	**0.9787**	0.9753	0.9786	0.9811
	V_{isub}	**0.9776**	**0.9616**	**0.9991**	0.9990	0.9983	0.9995	**0.9787**	0.9787	0.9163	0.9971	**0.9777**	0.9766	0.9829	0.9766	**0.9928**	0.9927	0.9816	0.9962	**0.9779**	0.9777	0.9484	0.9868
	T_{ex1}	**0.9643**	**0.9348**	**0.9925**	0.9960	0.9921	0.9924	**0.9686**	0.7442	0.9871	0.9772	**0.9643**	0.9877	0.9426	0.9784	**0.9811**	0.9806	0.9916	0.9732	**0.9653**	0.8489	0.9643	0.9778
	T_{ex2}	**0.9613**	**0.9325**	**0.9917**	0.9929	0.9925	0.9907	**0.9651**	0.7849	0.9705	0.9883	**0.9613**	0.9777	0.9713	0.9493	**0.9826**	0.9784	0.9757	0.9899	**0.9622**	0.8708	0.9709	0.9684
	T_{ex3}	**0.9627**	**0.9319**	**0.9960**	0.9965	0.9954	0.9970	**0.9642**	0.8502	0.9778	0.9739	**0.9627**	0.9553	0.9700	0.9502	**0.9759**	0.9819	0.9683	0.9883	**0.9631**	0.8997	0.9739	0.9623

T, training dataset. V, validation dataset. T_{imi}, training dataset after iterative manual improvement. V_{imi}, validation set after iterative manual improvement. V_{isub}, the subset of 5,672 patches in the V_{imi} that originated from WSIs different from those in the training set. T_{ex1}, RUMC-BRCA set. T_{ex2}, CPTAC-LUAD set. T_{ex3}, CPTAC-LUSC set. A, weighted average values for every index. P, TIL-positive patches label. N, TIL-negative patches label. O, other(non-tumor/necrotic) patches label. Kappa, Cohen's Kappa Statistic. Accuracy, Kappa, precision, recall, specificity and F1 score values of CAE-based clustering were calculated by comparing prediction classes with true labels.

Supplementary Table 11. Performance (accuracy) comparison of different models for TIL patch classification

Model source	BLCA	BRCA	CESC	COADREAD	HNSC	KIRC	KIRP	LUAD	LUSC	PRAD	SARC	SKCM	STAD	THCA	UCEC	LIHC	CAUM	PAPE	TTKO	Average
Le H et al.		0.8900	-	-	-	-	-	-	-	-	-	-	-	-	-	-	-	-	-	0.8900
Saltz et al.		0.7490	-	-	-	-	-	0.7360	-	-	-	-	-	-	-	-	-	-	-	0.7956*****
Abousamra S et al.	0.8471	0.8716	0.7895	0.7949/0.6957	0.5385	0.8254	-	0.8629	0.8553	0.8358	0.9693	0.9024	0.7500	-	0.8000	**1.0000**	-	-	-	0.8743******
Xu H et al.	-	-	-	0,8006	-	-	-	-	-	-	-	-	-	-	-	-	-	-	-	0,8006
**TILscout**	**0.9762**	**0.9688**	**0.9710**	**0.9823**	**0.9735**	**0.9853**	**0.9831**	**0.9868**	**0.9867**	**0.9674**	**0.9944**	**0.9920**	**0.9819**	**0.9889**	**0.9859**	0.9566	**0.9720**	**0.9923**	**0.9710**	**0.9787*****

CAUM, consisting of CHOL, ACC, UCS, and MESO. PAPE, consisting of PAAD, PCPG, and ESCA. TTKO, consisting of TGCT, THYM, KICH, and OV.

*, average accuracy value in 13 cancer types (BLCA, BRCA, CESC, COAD, LUAD, LUSC, PAAD, PRAD, READ, SKCM, STAD, UCEC, UVM).

**, average accuracy value in 23 cancer types (ACC, BLCA, BRCA, CESC, COAD, ESCA, HNSC, KIRC, LIHC, LUAD, LUSC, MESO, OV, PAAD, PRAD, READ, SKCM, SARC, STAD, TGCT, THYM, UCEC, UVM).

***, average accuracy value in 28 cancer types.

Supplementary Table 12. Comparison of different methods

Model resource	Pan-cancer applicable	Manual annotation/ analysis during prediction	Multiple separate methods	Accuracy
Le H et al.	-	+	+	0.8900
Saltz et al.	±*	+	+	0.7956
Abousamra S et al.	±*	+	+	0.8743
Xu H et al.	-	+	+	0,8006
**TILScout**	**+**	**-**	**-**	**0.9787**

*, fewer cancer types were involved.
