# Supplementary material for: Prediction and analysis of tumor infiltrating lymphocytes across 28 cancers by TILScout using deep learning
Source: NPJ Precis Oncol. 2025 Mar 19;9:76. doi: 10.1038/s41698-025-00866-0 (PMC11923303; doi:10.1038/s41698-025-00866-0)
Supplement: Supplementary file 3 — Supplementary Data 3 [file 41698_2025_866_MOESM3_ESM.pdf]

N	Description	setSize	enrichmentScore	NES	pvalue	p.adjust	qvalue	rank
1	GO(BP): immune response	460	0.601399003345435	3.16679095695844	0.0009990	0.0265670	0.0238309	616
2	GO(BP): adaptive immune response	196	0.617490741224126	3.15165522761317	0.0009990	0.0265670	0.0238309	597
3	GO(BP): T cell activation	204	0.613084386998104	3.13716095354642	0.0009990	0.0265670	0.0238309	419
4	GO(BP): antigen receptor-mediated signaling pathway	82	0.660725561388195	3.12087334931358	0.0010070	0.0265670	0.0238309	596
5	GO(BP): lymphocyte mediated immunity	122	0.615172216414324	3.04258596840579	0.0009990	0.0265670	0.0238309	527
6	GO(BP): regulation of T cell activation	147	0.604350276097278	3.03734146749573	0.0009990	0.0265670	0.0238309	419
7	GO(BP): lymphocyte activation	274	0.58080502489054	3.01084759655203	0.0009990	0.0265670	0.0238309	429
8	GO(BP): leukocyte mediated immunity	152	0.597024699332743	3.00651283516784	0.0009990	0.0265670	0.0238309	527
9	GO(BP): regulation of immune system process	389	0.574250847824994	3.00477263725739	0.0009990	0.0265670	0.0238309	657
10	GO(BP): regulation of immune response	264	0.579917858793748	3.0011810629988	0.0009990	0.0265670	0.0238309	616
11	GO(BP): regulation of leukocyte cell-cell adhesion	138	0.599115876847611	2.99810110141967	0.0009990	0.0265670	0.0238309	419
12	GO(BP): T cell receptor signaling pathway	57	0.663031077921209	2.99177265205095	0.0010162	0.0265670	0.0238309	454
13	GO(BP): leukocyte cell-cell adhesion	151	0.592716073128298	2.98387562868408	0.0009990	0.0265670	0.0238309	419
14	GO(BP): regulation of lymphocyte activation	189	0.58560139210833	2.98032302891117	0.0009990	0.0265670	0.0238309	423
15	GO(BP): positive regulation of immune system process	304	0.569195582431659	2.95458072527932	0.0009990	0.0265670	0.0238309	605
16	GO(BP): positive regulation of immune response	222	0.574610177685273	2.95247697482164	0.0009990	0.0265670	0.0238309	598
17	GO(BP): immune effector process	200	0.576437985164438	2.94575010599302	0.0009990	0.0265670	0.0238309	527
18	GO(BP): positive regulation of leukocyte mediated cytotoxicity	37	0.699854168668476	2.93293416925359	0.0010570	0.0265670	0.0238309	492
19	GO(BP): regulation of leukocyte activation	205	0.572564045201313	2.93129191905624	0.0009990	0.0265670	0.0238309	423
20	GO(BP): positive regulation of cell killing	42	0.682581557752735	2.92826410613538	0.0010449	0.0265670	0.0238309	492
21	GO(BP): leukocyte mediated cytotoxicity	66	0.632022910541014	2.90718609043997	0.0010060	0.0265670	0.0238309	499
22	GO(BP): leukocyte activation	307	0.559023753206442	2.90200675416898	0.0009990	0.0265670	0.0238309	615
23	GO(BP): regulation of cell-cell adhesion	153	0.57377992089251	2.89182537643099	0.0009990	0.0265670	0.0238309	419
24	GO(BP): regulation of leukocyte mediated cytotoxicity	51	0.650595539350162	2.88763957231421	0.0010277	0.0265670	0.0238309	499
25	GO(BP): T cell differentiation	113	0.587028698197381	2.8782392527501	0.0010010	0.0265670	0.0238309	403
26	GO(BP): T cell selection	28	0.733826241254768	2.87321832648074	0.0010905	0.0265670	0.0238309	399
27	GO(BP): cell activation	327	0.552130209646687	2.86827053964997	0.0009990	0.0265670	0.0238309	615
28	GO(BP): immune response-regulating cell surface receptor signaling pathway	126	0.576701043710831	2.86733501286155	0.0009990	0.0265670	0.0238309	605
29	GO(BP): T cell mediated immunity	49	0.64641725906223	2.85554368919373	0.0010288	0.0265670	0.0238309	487
30	GO(BP): regulation of cell activation	214	0.555586382710963	2.85126490633594	0.0009990	0.0265670	0.0238309	423
31	GO(BP): adaptive immune response based on somatic recombination of immune receptors built from immunoglobulin superfamily domains	119	0.578182265023598	2.84866358238265	0.0009990	0.0265670	0.0238309	521
32	GO(BP): positive regulation of leukocyte cell-cell adhesion	116	0.578534950013805	2.84685820528248	0.0009990	0.0265670	0.0238309	573
33	GO(BP): T cell mediated cytotoxicity	25	0.746529138337817	2.84642456939333	0.0011111	0.0265670	0.0238309	458
34	GO(BP): positive regulation of lymphocyte mediated immunity	54	0.634592719650414	2.83899388890963	0.0010256	0.0265670	0.0238309	458
35	GO(BP): positive regulation of leukocyte mediated immunity	60	0.623320241189456	2.83642836769022	0.0010152	0.0265670	0.0238309	492
36	GO(BP): positive regulation of T cell activation	109	0.580835944001646	2.83608464401897	0.001	0.0265670	0.0238309	419
37	GO(BP): immune response-activating cell surface receptor signaling pathway	115	0.576655776355954	2.83420807865669	0.001	0.0265670	0.0238309	605

38	GO(BP): regulation of leukocyte mediated immunity	97	0.587316731701653	2.83112575555302	0.0009990	0.0265670	0.0238309	499
39	GO(BP): regulation of cell killing	58	0.623884057821557	2.82484287384936	0.0010162	0.0265670	0.0238309	499
40	GO(BP): positive regulation of leukocyte activation	139	0.563091031458387	2.82247736523318	0.0009990	0.0265670	0.0238309	419
41	GO(BP): regulation of lymphocyte mediated immunity	78	0.600076129323339	2.82188986962686	0.0010080	0.0265670	0.0238309	499
42	GO(BP): lymphocyte differentiation	148	0.559759454229382	2.81610897061983	0.0009990	0.0265670	0.0238309	597
43	GO(BP): positive T cell selection	19	0.788036503668883	2.81609397813777	0.0011402	0.0265670	0.0238309	399
44	GO(BP): alpha-beta T cell activation	83	0.594227434628097	2.81329141286258	0.0010050	0.0265670	0.0238309	573
45	GO(BP): positive regulation of cell-cell adhesion	118	0.570849943078051	2.80934475365564	0.0009990	0.0265670	0.0238309	573
46	GO(BP): regulation of immune effector process	123	0.564380150905333	2.79392557070925	0.0009990	0.0265670	0.0238309	499
47	GO(BP): positive regulation of cell activation	143	0.555704465587198	2.7917063629052	0.0009990	0.0265670	0.0238309	419
48	GO(BP): positive regulation of lymphocyte activation	133	0.559009730330044	2.78542399297504	0.001	0.0265670	0.0238309	419
49	GO(BP): regulation of T cell mediated cytotoxicity	23	0.742196610706985	2.78530467765476	0.0011210	0.0265670	0.0238309	458
50	GO(BP): regulation of lymphocyte differentiation	82	0.588932392575403	2.7817652531475	0.0010070	0.0265670	0.0238309	419
51	GO(BP): regulation of T cell mediated immunity	40	0.656370545314624	2.77543141712022	0.0010537	0.0265670	0.0238309	487
52	GO(BP): cell killing	75	0.591752987214727	2.76932623156122	0.0010080	0.0265670	0.0238309	499
53	GO(BP): mononuclear cell differentiation	158	0.546306137126668	2.76528874702014	0.0009990	0.0265670	0.0238309	598
54	GO(BP): mononuclear cell proliferation	123	0.556165246562293	2.7532582452051	0.0009990	0.0265670	0.0238309	573
55	GO(BP): lymphocyte proliferation	123	0.556165246562293	2.7532582452051	0.0009990	0.0265670	0.0238309	573
56	GO(BP): T cell differentiation in thymus	32	0.676409769163931	2.73420698285682	0.0010706	0.0265670	0.0238309	403
57	GO(BP): cell-cell adhesion	193	0.534742722811605	2.72596347525559	0.0009990	0.0265670	0.0238309	419
58	GO(BP): negative regulation of immune system process	137	0.545514769034899	2.72590844009256	0.0009990	0.0265670	0.0238309	624
59	GO(BP): positive regulation of immune effector process	93	0.568580268387725	2.72444212030567	0.001	0.0265670	0.0238309	615
60	GO(BP): regulation of mononuclear cell proliferation	101	0.561454188141635	2.71419374042617	0.0009990	0.0265670	0.0238309	403
61	GO(BP): regulation of lymphocyte proliferation	101	0.561454188141635	2.71419374042617	0.0009990	0.0265670	0.0238309	403
62	GO(BP): leukocyte proliferation	130	0.542657561712696	2.70742196142399	0.0009990	0.0265670	0.0238309	601
63	GO(BP): activation of immune response	159	0.534562000342641	2.70589061200418	0.0009990	0.0265670	0.0238309	605
64	GO(BP): antigen processing and presentation of exogenous antigen	30	0.679023074534483	2.70184725534794	0.0010799	0.0265670	0.0238309	498
65	GO(BP): regulation of leukocyte proliferation	106	0.554202518967253	2.69290627327903	0.001	0.0265670	0.0238309	640
66	GO(BP): immune response-activating signaling pathway	150	0.534572422855955	2.69013077099376	0.0009990	0.0265670	0.0238309	624
67	GO(BP): regulation of cell adhesion	187	0.52876249472349	2.68949367506265	0.0009990	0.0265670	0.0238309	419
68	GO(BP): innate immune response	223	0.522761167365598	2.68504829487575	0.0009990	0.0265670	0.0238309	636
69	GO(BP): T cell proliferation	88	0.563443349095282	2.68440821584658	0.0010020	0.0265670	0.0238309	456
70	GO(BP): regulation of antigen receptor-mediated signaling pathway	31	0.668883200153793	2.68322524399288	0.0010729	0.0265670	0.0238309	557
71	GO(BP): immune response-regulating signaling pathway	159	0.529325032616835	2.67938169106415	0.0009990	0.0265670	0.0238309	605
72	GO(BP): B cell receptor signaling pathway	34	0.654464839420787	2.67612699846306	0.0010649	0.0265670	0.0238309	566
73	GO(BP): regulation of T cell proliferation	76	0.571299343627841	2.6725111417666	0.0010101	0.0265670	0.0238309	573
74	GO(BP): positive regulation of T cell mediated cytotoxicity	18	0.754226436910468	2.66589428965698	0.0011454	0.0265670	0.0238309	458
75	GO(BP): natural killer cell mediated immunity	43	0.617772276147314	2.65185178179612	0.0010471	0.0265670	0.0238309	635
76	GO(BP): antigen processing and presentation	52	0.593961961628682	2.64891018998854	0.0010245	0.0265670	0.0238309	596

77	GO(BP): positive regulation of cell adhesion	136	0.529658724493681	2.64653157352968	0.0009990	0.0265670	0.0238309	573
78	GO(BP): natural killer cell mediated cytotoxicity	42	0.614370059986467	2.63563785764383	0.0010449	0.0265670	0.0238309	635
79	GO(BP): production of molecular mediator of immune response	68	0.571746172401401	2.63441552048951	0.0010080	0.0265670	0.0238309	487
80	GO(BP): response to external biotic stimulus	325	0.505336371655818	2.62500905345089	0.0009990	0.0265670	0.0238309	616
81	GO(BP): response to other organism	325	0.505336371655818	2.62500905345089	0.0009990	0.0265670	0.0238309	616
82	GO(BP): positive regulation of alpha-beta T cell activation	38	0.621817815782804	2.61922340416587	0.0010559	0.0265670	0.0238309	561
83	GO(BP): regulation of T cell differentiation	71	0.563892137501638	2.61841661830465	0.0010060	0.0265670	0.0238309	419
84	GO(BP): regulation of cytokine production	201	0.510724544435275	2.61074245814739	0.0009990	0.0265670	0.0238309	600
85	GO(BP): defense response to other organism	268	0.503550917336918	2.61002374226508	0.0009990	0.0265670	0.0238309	616
86	GO(BP): cytokine production	205	0.507294748970294	2.59714002424558	0.0009990	0.0265670	0.0238309	626
87	GO(BP): positive regulation of cytokine production	146	0.516340635293182	2.59486047052104	0.0009990	0.0265670	0.0238309	598
88	GO(BP): cell activation involved in immune response	99	0.536140475803929	2.59045393645587	0.0009990	0.0265670	0.0238309	635
89	GO(BP): leukocyte activation involved in immune response	99	0.536140475803929	2.59045393645587	0.0009990	0.0265670	0.0238309	635
90	GO(BP): leukocyte differentiation	184	0.509405246253015	2.5904537835036	0.0009990	0.0265670	0.0238309	598
91	GO(BP): alpha-beta T cell differentiation	50	0.583963296272317	2.58779615037035	0.0010288	0.0265670	0.0238309	419
92	GO(BP): response to biotic stimulus	334	0.497787429525677	2.5868521759924	0.0009990	0.0265670	0.0238309	616
93	GO(BP): defense response	386	0.494368474048317	2.58557485271815	0.0009990	0.0265670	0.0238309	823
94	GO(BP): regulation of leukocyte differentiation	101	0.534398952616352	2.58340274721702	0.0009990	0.0265670	0.0238309	419
95	GO(BP): antigen processing and presentation of endogenous antigen	20	0.705257568215174	2.57174827925253	0.0011235	0.0265670	0.0238309	558
96	GO(BP): positive regulation of T cell mediated immunity	28	0.654444878656058	2.56240907358508	0.0010905	0.0265670	0.0238309	487
97	GO(BP): negative regulation of leukocyte activation	74	0.547433816012203	2.56017758008023	0.0010070	0.0265670	0.0238309	800
98	GO(BP): negative regulation of leukocyte mediated immunity	31	0.636865343828478	2.55478559962617	0.0010729	0.0265670	0.0238309	402
99	GO(BP): positive regulation of adaptive immune response based on somatic recombination of immune receptors built from immunoglobulin superfamily domains	49	0.577929585493801	2.55299987356355	0.0010288	0.0265670	0.0238309	487
100	GO(BP): regulation of adaptive immune response based on somatic recombination of immune receptors built from immunoglobulin superfamily domains	71	0.548974249871339	2.54914584419129	0.0010060	0.0265670	0.0238309	458
101	GO(BP): positive regulation of T cell proliferation	54	0.569186351834799	2.54638372687279	0.0010256	0.0265670	0.0238309	456
102	GO(BP): negative regulation of lymphocyte activation	62	0.555113140120505	2.53629937843032	0.0010111	0.0265670	0.0238309	729
103	GO(BP): immunoglobulin production	42	0.591146175721802	2.536007760808	0.0010449	0.0265670	0.0238309	487
104	GO(BP): regulation of hemopoiesis	115	0.515809072331127	2.53515233833866	0.001	0.0265670	0.0238309	571
105	GO(BP): positive regulation of natural killer cell mediated immunity	20	0.693854912142218	2.53016806451092	0.0011235	0.0265670	0.0238309	349
106	GO(BP): antigen processing and presentation of peptide antigen	33	0.625347228734394	2.5298098459691	0.0010706	0.0265670	0.0238309	588
107	GO(BP): regulation of adaptive immune response	78	0.536640139209658	2.52357875687954	0.0010080	0.0265670	0.0238309	458
108	GO(BP): interleukin-12 production	29	0.638262337133558	2.51788724173886	0.0010869	0.0265670	0.0238309	395
109	GO(BP): regulation of interleukin-12 production	29	0.638262337133558	2.51788724173886	0.0010869	0.0265670	0.0238309	395
110	GO(BP): positive regulation of adaptive immune response	50	0.567552711625523	2.51507368982293	0.0010288	0.0265670	0.0238309	487
111	GO(BP): positive regulation of mononuclear cell proliferation	69	0.542687673523641	2.50783350115689	0.0010080	0.0265670	0.0238309	399

112	GO(BP): positive regulation of lymphocyte proliferation	69	0.542687673523641	2.50783350115689	0.0010080	0.0265670	0.0238309	399
113	GO(BP): regulation of natural killer cell mediated immunity	32	0.620132594277068	2.50672143849321	0.0010706	0.0265670	0.0238309	499
114	GO(BP): negative regulation of immune response	63	0.54659561754745	2.50256489098287	0.0010111	0.0265670	0.0238309	624
115	GO(BP): gamma-delta T cell activation	15	0.750134874269957	2.49971536980764	0.0011820	0.0265670	0.0238309	351
116	GO(BP): biological process involved in interspecies interaction between organisms	349	0.479479661302008	2.49624436407482	0.0009990	0.0265670	0.0238309	616
117	GO(BP): negative regulation of cell activation	77	0.532617361383948	2.49605587861272	0.0010101	0.0265670	0.0238309	800
118	GO(BP): regulation of alpha-beta T cell activation	57	0.552038270784706	2.49094357175131	0.0010162	0.0265670	0.0238309	573
119	GO(BP): positive regulation of lymphocyte differentiation	52	0.557604694856503	2.48676658373996	0.0010245	0.0265670	0.0238309	561
120	GO(BP): positive regulation of leukocyte proliferation	73	0.531409426269503	2.47938040937	0.0010070	0.0265670	0.0238309	657
121	GO(BP): regulation of production of molecular mediator of immune response	51	0.557253249341419	2.47334393993268	0.0010277	0.0265670	0.0238309	402
122	GO(BP): B cell mediated immunity	52	0.553779237435904	2.46970607515957	0.0010245	0.0265670	0.0238309	820
123	GO(BP): hemopoiesis	227	0.480353300724422	2.46969660115887	0.0009990	0.0265670	0.0238309	598
124	GO(BP): regulation of natural killer cell mediated cytotoxicity	31	0.615179437443166	2.46779257687039	0.0010729	0.0265670	0.0238309	624
125	GO(BP): antigen processing and presentation of exogenous peptide antigen	24	0.650798683714741	2.4650105938106	0.0011111	0.0265670	0.0238309	498
126	GO(BP): regulation of T cell receptor signaling pathway	22	0.667699607781003	2.46079553219113	0.0011337	0.0265670	0.0238309	596
127	GO(BP): leukocyte migration	95	0.512673113582929	2.45671840721017	0.001	0.0265670	0.0238309	612
128	GO(BP): positive regulation of natural killer cell mediated cytotoxicity	19	0.686944716759242	2.45483663659329	0.0011402	0.0265670	0.0238309	349
129	GO(BP): negative regulation of immune effector process	38	0.582693498943309	2.45442380573525	0.0010559	0.0265670	0.0238309	402
130	GO(BP): positive regulation of leukocyte differentiation	61	0.536301825775932	2.44859270962601	0.0010131	0.0265670	0.0238309	561
131	GO(BP): positive regulation of hemopoiesis	61	0.536301825775932	2.44859270962601	0.0010131	0.0265670	0.0238309	561
132	GO(BP): immunoglobulin mediated immune response	51	0.551155137260028	2.44627773874074	0.0010277	0.0265670	0.0238309	820
133	GO(BP): positive regulation of response to stimulus	397	0.465356730455316	2.4333957299156	0.0009990	0.0265670	0.0238309	616
134	GO(BP): cell adhesion mediated by integrin	18	0.687898487666749	2.43145103431604	0.0011454	0.0265670	0.0238309	408
135	GO(BP): cytokine production involved in immune response	34	0.591571745273698	2.418955188572	0.0010649	0.0265670	0.0238309	487
136	GO(BP): regulation of cytokine production involved in immune response	34	0.591571745273698	2.418955188572	0.0010649	0.0265670	0.0238309	487
137	GO(BP): regulation of leukocyte apoptotic process	32	0.597983071133445	2.41718786933572	0.0010706	0.0265670	0.0238309	596
138	GO(BP): regulation of T cell apoptotic process	19	0.674382655194876	2.40994538376464	0.0011402	0.0265670	0.0238309	571
139	GO(BP): regulation of innate immune response	114	0.488829002061919	2.40321480120615	0.001	0.0265670	0.0238309	624
140	GO(BP): positive regulation of multicellular organismal process	291	0.46220324678978	2.39736084102356	0.0009990	0.0265670	0.0238309	625
141	GO(BP): type II interferon production	53	0.536523470610505	2.39701416108034	0.0010235	0.0265670	0.0238309	624
142	GO(BP): regulation of type II interferon production	53	0.536523470610505	2.39701416108034	0.0010235	0.0265670	0.0238309	624
143	GO(BP): negative regulation of T cell activation	49	0.541442444717092	2.3918181861612	0.0010288	0.0265670	0.0238309	792
144	GO(BP): cell adhesion	270	0.459246826159779	2.3826325570776	0.0009990	0.0265670	0.0238309	419
145	GO(BP): thymic T cell selection	15	0.714762322142223	2.38184148438261	0.0011820	0.0265670	0.0238309	341
146	GO(BP): antigen processing and presentation via MHC class Ib	13	0.741565411867153	2.37863448638053	0.0012210	0.0265670	0.0238309	458
147	GO(BP): positive regulation of CD4-positive, alpha-beta T cell activation	24	0.627393349279793	2.37635891276461	0.0011111	0.0265670	0.0238309	561
148	GO(BP): lymphocyte migration	43	0.553418694390695	2.37560733536266	0.0010471	0.0265670	0.0238309	345
149	GO(BP): lymphocyte activation involved in immune response	72	0.510323364012366	2.37456134829645	0.0010060	0.0265670	0.0238309	635

150	GO(BP): B cell activation	95	0.494548484566235	2.36986557925908	0.001	0.0265670	0.0238309	612
151	GO(BP): negative regulation of leukocyte cell-cell adhesion	51	0.533327012845109	2.36714839578201	0.0010277	0.0265670	0.0238309	792
152	GO(BP): negative regulation of antigen receptor-mediated signaling pathway	16	0.699134119310034	2.365865261481	0.0011792	0.0265670	0.0238309	548
153	GO(BP): regulation of leukocyte migration	57	0.523859915497136	2.36379533460768	0.0010162	0.0265670	0.0238309	612
154	GO(BP): regulation of B cell activation	55	0.525139971619522	2.3609198560383	0.0010214	0.0265670	0.0238309	423
155	GO(BP): negative regulation of T cell mediated immunity	13	0.735819555300455	2.36020415998636	0.0012210	0.0265670	0.0238309	402
156	GO(BP): regulation of lymphocyte apoptotic process	25	0.618346404202836	2.35767943531027	0.0011111	0.0265670	0.0238309	571
157	GO(BP): CD4-positive, alpha-beta T cell activation	49	0.531872353154541	2.34954237408574	0.0010288	0.0265670	0.0238309	597
158	GO(BP): positive regulation of type II interferon production	40	0.554943708729884	2.34655289597715	0.0010537	0.0265670	0.0238309	803
159	GO(BP): mononuclear cell migration	57	0.520008224518439	2.34641548000064	0.0010162	0.0265670	0.0238309	345
160	GO(BP): positive regulation of T cell differentiation	45	0.539914893316274	2.34470127889702	0.0010373	0.0265670	0.0238309	561
161	GO(BP): response to external stimulus	462	0.444373215535989	2.34048986870693	0.0009990	0.0265670	0.0238309	619
162	GO(BP): negative regulation of leukocyte apoptotic process	25	0.613429623627395	2.33893235055024	0.0011111	0.0265670	0.0238309	319
163	GO(BP): regulation of defense response	191	0.4580946066582	2.33312296192324	0.0009990	0.0265670	0.0238309	800
164	GO(BP): positive regulation of response to external stimulus	146	0.463279736055199	2.32820388656893	0.0009990	0.0265670	0.0238309	900
165	GO(BP): negative regulation of leukocyte proliferation	38	0.552473636868053	2.32713158603818	0.0010559	0.0265670	0.0238309	729
166	GO(BP): negative regulation of lymphocyte mediated immunity	26	0.604960894475035	2.32581694620484	0.0011025	0.0265670	0.0238309	454
167	GO(BP): regulation of response to biotic stimulus	138	0.463885512640531	2.32138008710117	0.0009990	0.0265670	0.0238309	799
168	GO(BP): response to bacterium	129	0.465163654338481	2.31530671679072	0.001	0.0265670	0.0238309	717
169	GO(BP): myeloid cell activation involved in immune response	34	0.566029979046325	2.31451411538922	0.0010649	0.0265670	0.0238309	624
170	GO(BP): positive regulation of alpha-beta T cell differentiation	23	0.616251154032775	2.312658394253	0.0011210	0.0265670	0.0238309	561
171	GO(BP): response to type II interferon	46	0.531351072458799	2.30758505268742	0.0010351	0.0265670	0.0238309	692
172	GO(BP): immunoglobulin production involved in immunoglobulin-mediated immune response	29	0.584844318825766	2.3071579867742	0.0010869	0.0265670	0.0238309	417
173	GO(BP): cellular response to type II interferon	39	0.542273874712494	2.29927446692462	0.0010471	0.0265670	0.0238309	805
174	GO(BP): positive regulation of production of molecular mediator of immune response	40	0.543626125180225	2.2986970359392	0.0010537	0.0265670	0.0238309	363
175	GO(BP): B cell differentiation	50	0.516752840764856	2.28995729793374	0.0010288	0.0265670	0.0238309	612
176	GO(BP): antigen processing and presentation of peptide antigen via MHC class II	18	0.647761254138794	2.2895816746273	0.0011454	0.0265670	0.0238309	498
177	GO(BP): antigen processing and presentation of peptide or polysaccharide antigen via MHC class II	18	0.647761254138794	2.2895816746273	0.0011454	0.0265670	0.0238309	498
178	GO(BP): antigen processing and presentation of exogenous peptide antigen via MHC class II	18	0.647761254138794	2.2895816746273	0.0011454	0.0265670	0.0238309	498
179	GO(BP): negative regulation of lymphocyte apoptotic process	19	0.640127024036927	2.28753090655186	0.0011402	0.0265670	0.0238309	395
180	GO(BP): negative regulation of T cell apoptotic process	14	0.700163216344227	2.2849065941373	0.0012180	0.0265670	0.0238309	395
181	GO(BP): negative regulation of innate immune response	28	0.583512286561377	2.28468007986208	0.0010905	0.0265670	0.0238309	653
182	GO(BP): negative regulation of mononuclear cell proliferation	36	0.547864466186404	2.28113584814228	0.0010626	0.0265670	0.0238309	729
183	GO(BP): negative regulation of lymphocyte proliferation	36	0.547864466186404	2.28113584814228	0.0010626	0.0265670	0.0238309	729

184	GO(BP): T cell migration	28	0.582350625900407	2.2801317215279	0.0010905	0.0265670	0.0238309	345
185	GO(BP): cell chemotaxis	72	0.489838510633296	2.27924425233397	0.0010060	0.0265670	0.0238309	351
186	GO(BP): T cell costimulation	26	0.590942540379757	2.27192234605719	0.0011025	0.0265670	0.0238309	640
187	GO(BP): cellular defense response	26	0.590475959706618	2.27012854211669	0.0011025	0.0265670	0.0238309	612
188	GO(BP): negative regulation of production of molecular mediator of immune response	12	0.719461315386509	2.26660390891589	0.0012300	0.0265670	0.0238309	402
189	GO(BP): myeloid leukocyte activation	71	0.486858644132685	2.26071384894702	0.0010060	0.0265670	0.0238309	710
190	GO(BP): regulation of multicellular organismal process	457	0.429285969123836	2.25720048368196	0.0009990	0.0265670	0.0238309	625
191	GO(BP): CD4-positive, alpha-beta T cell differentiation	38	0.535494490947425	2.25561196204338	0.0010559	0.0265670	0.0238309	597
192	GO(BP): immune system development	54	0.504146262119191	2.25541219266032	0.0010256	0.0265670	0.0238309	412
193	GO(BP): MHC class II protein complex assembly	13	0.702813189607503	2.25433341891489	0.0012210	0.0265670	0.0238309	417
194	GO(BP): peptide antigen assembly with MHC class II protein complex	13	0.702813189607503	2.25433341891489	0.0012210	0.0265670	0.0238309	417
195	GO(BP): leukocyte degranulation	30	0.566236468374202	2.25306695063874	0.0010799	0.0265670	0.0238309	448
196	GO(BP): antigen processing and presentation of endogenous peptide antigen	14	0.689665458605869	2.25064830218465	0.0012180	0.0265670	0.0238309	558
197	GO(BP): regulation of cell development	151	0.445351036880723	2.24200450334325	0.0009990	0.0265670	0.0238309	571
198	GO(BP): B cell proliferation	43	0.520838291527467	2.23575256570012	0.0010471	0.0265670	0.0238309	543
199	GO(BP): positive regulation of B cell activation	33	0.552506981273622	2.23513839506649	0.0010706	0.0265670	0.0238309	351
200	GO(BP): positive regulation of defense response	119	0.453140019787923	2.23258918541427	0.0009990	0.0265670	0.0238309	665
201	GO(BP): negative regulation of multicellular organismal process	183	0.43888960140894	2.23253666741902	0.0009990	0.0265670	0.0238309	805
202	GO(BP): CD8-positive, alpha-beta T cell activation	20	0.612208140016989	2.23244003551441	0.0011235	0.0265670	0.0238309	559
203	GO(BP): regulation of B cell differentiation	18	0.631530905280401	2.23221376464264	0.0011454	0.0265670	0.0238309	396
204	GO(BP): positive regulation of immunoglobulin production	17	0.643489207082908	2.23001272031003	0.0011655	0.0265670	0.0238309	363
205	GO(BP): interleukin-2 production	27	0.572710860037526	2.22397961326075	0.0010917	0.0265670	0.0238309	624
206	GO(BP): regulation of interleukin-2 production	27	0.572710860037526	2.22397961326075	0.0010917	0.0265670	0.0238309	624
207	GO(BP): cell surface receptor signaling pathway	454	0.422668627133145	2.2213547832405	0.0009990	0.0265670	0.0238309	616
208	GO(BP): regulation of response to external stimulus	242	0.430664033025095	2.21842798289641	0.0009990	0.0265670	0.0238309	822
209	GO(BP): leukocyte apoptotic process	39	0.522956818508714	2.21736896460064	0.0010471	0.0265670	0.0238309	596
210	GO(BP): mast cell activation	25	0.581496048596701	2.21717352308061	0.0011111	0.0265670	0.0238309	355
211	GO(BP): negative regulation of T cell receptor signaling pathway	13	0.690859738902553	2.21599170337229	0.0012210	0.0265670	0.0238309	548
212	GO(BP): regulation of alpha-beta T cell differentiation	31	0.552296219513227	2.21553652120279	0.0010729	0.0265670	0.0238309	561
213	GO(BP): cellular response to cytokine stimulus	181	0.435360198517462	2.21488867421031	0.0009990	0.0265670	0.0238309	658
214	GO(BP): alpha-beta T cell proliferation	29	0.560147210064151	2.20973012452875	0.0010869	0.0265670	0.0238309	729
215	GO(BP): negative regulation of cell-cell adhesion	63	0.482582619356076	2.2094840892028	0.0010111	0.0265670	0.0238309	793
216	GO(BP): positive regulation of alpha-beta T cell proliferation	18	0.624929730517236	2.20888120396201	0.0011454	0.0265670	0.0238309	456
217	GO(BP): leukocyte chemotaxis	60	0.484569403604621	2.20504054204317	0.0010152	0.0265670	0.0238309	382
218	GO(BP): T cell activation involved in immune response	43	0.513614236487116	2.20474255769196	0.0010471	0.0265670	0.0238309	624
219	GO(BP): defense response to bacterium	42	0.513092184891825	2.2011573724668	0.0010449	0.0265670	0.0238309	624
220	GO(BP): negative regulation of cell adhesion	73	0.471216297517775	2.19853920327135	0.0010070	0.0265670	0.0238309	793
221	GO(BP): positive regulation of response to biotic stimulus	95	0.458041254264921	2.19492372586112	0.001	0.0265670	0.0238309	892

222	GO(BP): natural killer cell activation	40	0.517899894851646	2.18991490303014	0.0010537	0.0265670	0.0238309	720
223	GO(BP): neutrophil chemotaxis	27	0.563813306336277	2.18942818527112	0.0010917	0.0265670	0.0238309	382
224	GO(BP): regulation of calcium-mediated signaling	19	0.611249690683373	2.18433608729792	0.0011402	0.0265670	0.0238309	351
225	GO(BP): positive regulation of gene expression	197	0.427874470489468	2.18348520187769	0.0009990	0.0265670	0.0238309	598
226	GO(BP): regulatory T cell differentiation	22	0.591877967392718	2.18135616793724	0.0011337	0.0265670	0.0238309	553
227	GO(BP): lymphocyte costimulation	28	0.556178802662731	2.1776587409551	0.0010905	0.0265670	0.0238309	640
228	GO(BP): positive regulation of CD4-positive, alpha-beta T cell differentiation	18	0.615758704049331	2.17646522661808	0.0011454	0.0265670	0.0238309	561
229	GO(BP): lymphocyte homeostasis	31	0.542368801199842	2.17571267838535	0.0010729	0.0265670	0.0238309	583
230	GO(BP): positive regulation of leukocyte migration	38	0.51484941977732	2.16865071356044	0.0010559	0.0265670	0.0238309	345
231	GO(BP): heterotypic cell-cell adhesion	12	0.687939301425188	2.16729638739439	0.0012300	0.0265670	0.0238309	446
232	GO(BP): MHC protein complex assembly	16	0.640415444710082	2.16716165283245	0.0011792	0.0265670	0.0238309	417
233	GO(BP): peptide antigen assembly with MHC protein complex	16	0.640415444710082	2.16716165283245	0.0011792	0.0265670	0.0238309	417
234	GO(BP): cytokine-mediated signaling pathway	128	0.435265125436907	2.16713230733146	0.0009990	0.0265670	0.0238309	815
235	GO(BP): positive regulation of lymphocyte migration	14	0.66361644098211	2.1656401630109	0.0012180	0.0265670	0.0238309	345
236	GO(BP): lymphocyte apoptotic process	31	0.539745165036959	2.16518796079387	0.0010729	0.0265670	0.0238309	571
237	GO(BP): antigen processing and presentation of peptide antigen via MHC class I	18	0.612484222720036	2.16489122092145	0.0011454	0.0265670	0.0238309	588
238	GO(BP): T cell apoptotic process	25	0.567561975890554	2.16404460303496	0.0011111	0.0265670	0.0238309	571
239	GO(BP): positive regulation of innate immune response	91	0.452644372550196	2.16266091605088	0.0010010	0.0265670	0.0238309	624
240	GO(BP): chemotaxis	104	0.445727863827166	2.16113858547963	0.001	0.0265670	0.0238309	351
241	GO(BP): taxis	104	0.445727863827166	2.16113858547963	0.001	0.0265670	0.0238309	351
242	GO(BP): neutrophil migration	33	0.533616526723293	2.15871803895014	0.0010706	0.0265670	0.0238309	596
243	GO(BP): calcium-mediated signaling	36	0.517711565649717	2.15558862508821	0.0010626	0.0265670	0.0238309	448
244	GO(BP): phagocytosis	62	0.470988200407966	2.1519344321113	0.0010111	0.0265670	0.0238309	425
245	GO(BP): response to cytokine	198	0.420819224436597	2.14914465263555	0.0009990	0.0265670	0.0238309	693
246	GO(BP): antigen processing and presentation of endogenous peptide antigen via MHC class I	12	0.681619702295186	2.1473870082735	0.0012300	0.0265670	0.0238309	558
247	GO(BP): positive regulation of cytokine production involved in immune response	27	0.552614847441883	2.1459417665232	0.0010917	0.0265670	0.0238309	615
248	GO(BP): inflammatory response	198	0.420094352283334	2.14544269459379	0.0009990	0.0265670	0.0238309	800
249	GO(BP): leukocyte homeostasis	38	0.508247096355171	2.14084038135342	0.0010559	0.0265670	0.0238309	597
250	GO(BP): regulation of alpha-beta T cell proliferation	27	0.550933271303069	2.13941178549468	0.0010917	0.0265670	0.0238309	729
251	GO(BP): regulation of immunoglobulin production	24	0.564831919585569	2.13939686778944	0.0011111	0.0265670	0.0238309	399
252	GO(BP): small GTPase mediated signal transduction	87	0.44889886011211	2.13584602378098	0.0010040	0.0265670	0.0238309	391
253	GO(BP): regulation of phosphatidylinositol 3-kinase signaling	19	0.597160026990012	2.13398585999737	0.0011402	0.0265670	0.0238309	405
254	GO(BP): negative regulation of leukocyte mediated cytotoxicity	15	0.639347867796088	2.13053378345995	0.0011820	0.0265670	0.0238309	318
255	GO(BP): positive regulation of cell population proliferation	147	0.42350461118287	2.1284479681686	0.0009990	0.0265670	0.0238309	408
256	GO(BP): positive regulation of cell development	87	0.446717746626005	2.12546835749431	0.0010040	0.0265670	0.0238309	561
257	GO(BP): positive regulation of interleukin-12 production	19	0.593168163319163	2.1197207045221	0.0011402	0.0265670	0.0238309	615

258	GO(BP): negative regulation of adaptive immune response	26	0.550975308879218	2.11826536563781	0.0011025	0.0265670	0.0238309	402
259	GO(BP): tolerance induction	16	0.625854366166091	2.11788705880902	0.0011792	0.0265670	0.0238309	624
260	GO(BP): myeloid leukocyte migration	51	0.477163849268367	2.1178706743077	0.0010277	0.0265670	0.0238309	612
261	GO(BP): positive regulation of myeloid cell differentiation	20	0.58030501896703	2.11610410328094	0.0011235	0.0265670	0.0238309	294
262	GO(BP): tumor necrosis factor superfamily cytokine production	52	0.474336819454721	2.11541431221459	0.0010245	0.0265670	0.0238309	892
263	GO(BP): regulation of tumor necrosis factor superfamily cytokine production	52	0.474336819454721	2.11541431221459	0.0010245	0.0265670	0.0238309	892
264	GO(BP): phosphatidylinositol 3-kinase signaling	29	0.535769108722509	2.11356071772715	0.0010869	0.0265670	0.0238309	405
265	GO(BP): regulation of multicellular organismal development	195	0.414061714123064	2.11268040745976	0.0009990	0.0265670	0.0238309	587
266	GO(BP): positive regulation of mononuclear cell migration	23	0.560919770379568	2.10501158007045	0.0011210	0.0265670	0.0238309	612
267	GO(BP): regulation of inflammatory response	96	0.435988253816259	2.09964739046506	0.0009990	0.0265670	0.0238309	800
268	GO(BP): regulation of B cell proliferation	32	0.519266881801277	2.09899856405422	0.0010706	0.0265670	0.0238309	366
269	GO(BP): regulation of leukocyte degranulation	17	0.605162531281535	2.09719157331409	0.0011655	0.0265670	0.0238309	553
270	GO(BP): Fc receptor signaling pathway	16	0.618230162032162	2.09208680216484	0.0011792	0.0265670	0.0238309	635
271	GO(BP): regulation of CD4-positive, alpha-beta T cell activation	36	0.502449270704764	2.09204121460145	0.0010626	0.0265670	0.0238309	561
272	GO(BP): negative regulation of leukocyte differentiation	28	0.533963461788499	2.09067694480167	0.0010905	0.0265670	0.0238309	792
273	GO(BP): negative regulation of hemopoiesis	28	0.533963461788499	2.09067694480167	0.0010905	0.0265670	0.0238309	792
274	GO(BP): positive regulation of phosphatidylinositol 3-kinase signaling	13	0.650987813306702	2.08809909168495	0.0012210	0.0265670	0.0238309	309
275	GO(BP): regulated exocytosis	41	0.491991579666682	2.08769695592446	0.0010537	0.0265670	0.0238309	461
276	GO(BP): tumor necrosis factor production	50	0.470410755504822	2.08459529898351	0.0010288	0.0265670	0.0238309	892
277	GO(BP): regulation of tumor necrosis factor production	50	0.470410755504822	2.08459529898351	0.0010288	0.0265670	0.0238309	892
278	GO(BP): granulocyte migration	41	0.490545177222293	2.08155935092269	0.0010537	0.0265670	0.0238309	596
279	GO(BP): regulation of chemotaxis	49	0.471181748019827	2.08144205334678	0.0010288	0.0265670	0.0238309	382
280	GO(BP): negative regulation of defense response	67	0.451823762871228	2.08108601040414	0.0010070	0.0265670	0.0238309	800
281	GO(BP): response to chemokine	32	0.514498330719382	2.07972296180746	0.0010706	0.0265670	0.0238309	345
282	GO(BP): cellular response to chemokine	32	0.514498330719382	2.07972296180746	0.0010706	0.0265670	0.0238309	345
283	GO(BP): regulation of T cell migration	15	0.62352016831168	2.07779027688938	0.0011820	0.0265670	0.0238309	573
284	GO(BP): regulation of small GTPase mediated signal transduction	56	0.460667239397815	2.07710748017988	0.0010172	0.0265670	0.0238309	349
285	GO(BP): positive regulation of antigen receptor-mediated signaling pathway	13	0.646407598998018	2.07340766253344	0.0012210	0.0265670	0.0238309	396
286	GO(BP): phosphatidylinositol-mediated signaling	35	0.502712817528374	2.06951410629383	0.0010695	0.0265670	0.0238309	405
287	GO(BP): positive regulation of chemotaxis	34	0.504490529459479	2.06287740003005	0.0010649	0.0265670	0.0238309	345
288	GO(BP): negative regulation of adaptive immune response based on somatic recombination of immune receptors built from immunoglobulin superfamily domains	23	0.549167206260567	2.06090672787514	0.0011210	0.0265670	0.0238309	402
289	GO(BP): regulation of myeloid leukocyte mediated immunity	19	0.57650123438446	2.06016047096877	0.0011402	0.0265670	0.0238309	553
290	GO(BP): response to molecule of bacterial origin	76	0.440284590433657	2.05963036122198	0.0010101	0.0265670	0.0238309	717
291	GO(BP): regulation of lymphocyte migration	24	0.542916164503764	2.05638722164257	0.0011111	0.0265670	0.0238309	612
292	GO(BP): negative regulation of response to biotic stimulus	40	0.485900114176301	2.05460536292161	0.0010537	0.0265670	0.0238309	653
293	GO(BP): G protein-coupled receptor signaling pathway	120	0.416249376360462	2.05452072404137	0.0009990	0.0265670	0.0238309	814

294	GO(BP): interleukin-6 production	46	0.473042216691643	2.05435766503021	0.0010351	0.0265670	0.0238309	624
295	GO(BP): regulation of interleukin-6 production	46	0.473042216691643	2.05435766503021	0.0010351	0.0265670	0.0238309	624
296	GO(BP): regulation of ERK1 and ERK2 cascade	48	0.468345154370894	2.05260956543118	0.0010341	0.0265670	0.0238309	699
297	GO(BP): regulation of inflammatory response to antigenic stimulus	13	0.638950269144313	2.0494876391849	0.0012210	0.0265670	0.0238309	351
298	GO(BP): positive regulation of B cell mediated immunity	14	0.627519475709929	2.04784163824778	0.0012180	0.0265670	0.0238309	345
299	GO(BP): positive regulation of immunoglobulin mediated immune response	14	0.627519475709929	2.04784163824778	0.0012180	0.0265670	0.0238309	345
300	GO(BP): T cell cytokine production	17	0.59059978979148	2.04672437291981	0.0011655	0.0265670	0.0238309	568
301	GO(BP): regulation of T cell cytokine production	17	0.59059978979148	2.04672437291981	0.0011655	0.0265670	0.0238309	568
302	GO(BP): regulation of myeloid cell differentiation	42	0.476823807468322	2.04556660592651	0.0010449	0.0265670	0.0238309	684
303	GO(BP): inositol lipid-mediated signaling	36	0.490705694621479	2.04314464612086	0.0010626	0.0265670	0.0238309	405
304	GO(BP): leukocyte adhesion to vascular endothelial cell	13	0.635936171692191	2.03981966380444	0.0012210	0.0265670	0.0238309	569
305	GO(BP): negative regulation of T cell proliferation	26	0.530568745760497	2.03981082295781	0.0011025	0.0265670	0.0238309	666
306	GO(BP): interleukin-4 production	18	0.575684980033671	2.0348203481168	0.0011454	0.0265670	0.0238309	624
307	GO(BP): regulation of interleukin-4 production	18	0.575684980033671	2.0348203481168	0.0011454	0.0265670	0.0238309	624
308	GO(BP): positive regulation of interleukin-4 production	14	0.621792021474779	2.02915071355476	0.0012180	0.0265670	0.0238309	624
309	GO(BP): negative regulation of cytokine production	78	0.430917508334525	2.02641247000649	0.0010080	0.0265670	0.0238309	814
310	GO(BP): mast cell activation involved in immune response	19	0.566990897595397	2.02617473295149	0.0011402	0.0265670	0.0238309	601
311	GO(BP): regulation of regulatory T cell differentiation	19	0.566853026083313	2.02568204113693	0.0011402	0.0265670	0.0238309	553
312	GO(BP): negative regulation of cell killing	17	0.58437744646272	2.02516083367076	0.0011655	0.0265670	0.0238309	318
313	GO(BP): dendritic cell migration	17	0.584154677697165	2.02438882821145	0.0011655	0.0265670	0.0238309	597
314	GO(BP): chemokine-mediated signaling pathway	30	0.50866945725827	2.02400658904676	0.0010799	0.0265670	0.0238309	345
315	GO(BP): inflammatory response to antigenic stimulus	22	0.548436326788186	2.02125274138895	0.0011337	0.0265670	0.0238309	351
316	GO(BP): granulocyte chemotaxis	34	0.493939982305381	2.01973588594561	0.0010649	0.0265670	0.0238309	382
317	GO(BP): regulation of leukocyte chemotaxis	36	0.484792860475902	2.01852545877425	0.0010626	0.0265670	0.0238309	382
318	GO(BP): CD4-positive, alpha-beta T cell differentiation involved in immune response	29	0.511509844605614	2.01786011303813	0.0010869	0.0265670	0.0238309	597
319	GO(BP): peptidyl-tyrosine phosphorylation	66	0.438643543677224	2.01767750436156	0.0010060	0.0265670	0.0238309	678
320	GO(BP): peptidyl-tyrosine modification	66	0.438643543677224	2.01767750436156	0.0010060	0.0265670	0.0238309	678
321	GO(BP): positive regulation of interleukin-2 production	14	0.618263730878374	2.0176365204901	0.0012180	0.0265670	0.0238309	561
322	GO(BP): ERK1 and ERK2 cascade	49	0.456497964418065	2.01657654270382	0.0010288	0.0265670	0.0238309	699
323	GO(BP): defense response to Gram-positive bacterium	20	0.552754365993778	2.01563960978312	0.0011235	0.0265670	0.0238309	624
324	GO(BP): exocytosis	56	0.446584564679896	2.01360995637991	0.0010172	0.0265670	0.0238309	461
325	GO(BP): receptor signaling pathway via JAK-STAT	38	0.477973307401014	2.01332101064886	0.0010559	0.0265670	0.0238309	519
326	GO(BP): receptor signaling pathway via STAT	38	0.477973307401014	2.01332101064886	0.0010559	0.0265670	0.0238309	519
327	GO(BP): response to lipopolysaccharide	71	0.433481800637233	2.01286004013109	0.0010060	0.0265670	0.0238309	717
328	GO(BP): mast cell degranulation	17	0.578329041769799	2.00420007900024	0.0011655	0.0265670	0.0238309	448
329	GO(BP): positive thymic T cell selection	10	0.833668341708543	2.45073398588754	0.0012787	0.0267793	0.0240213	341
330	GO(BP): regulation of T cell differentiation in thymus	11	0.756881062560535	2.30329041443026	0.0012610	0.0267793	0.0240213	349
331	GO(BP): regulation of cell adhesion mediated by integrin	10	0.778513878298639	2.2885964652579	0.0012787	0.0267793	0.0240213	351

332	GO(BP): positive regulation of T cell migration	11	0.727815287710858	2.21483936985958	0.0012610	0.0267793	0.0240213	345
333	GO(BP): T cell lineage commitment	11	0.724558815569011	2.20492948911319	0.0012610	0.0267793	0.0240213	399
334	GO(BP): positive regulation of calcium-mediated signaling	11	0.693722753619103	2.11109122386749	0.0012610	0.0267793	0.0240213	351
335	GO(BP): B cell homeostasis	11	0.669318618651451	2.03682617361865	0.0012610	0.0267793	0.0240213	583
336	GO(BP): natural killer cell differentiation	11	0.660719348206898	2.01065744227395	0.0012610	0.0267793	0.0240213	416
337	GO(BP): regulation of B cell receptor signaling pathway	10	0.682090983561296	2.00514217854939	0.0012787	0.0267793	0.0240213	423
338	GO(BP): negative regulation of cytokine production involved in immune response	9	0.802611752887996	2.2654844762119	0.0013210	0.0274707	0.0246414	402
339	GO(BP): CD4-positive, CD25-positive, alpha-beta regulatory T cell differentiation	9	0.730620375494093	2.06227869543415	0.0013210	0.0274707	0.0246414	399
340	GO(BP): gamma-delta T cell differentiation	9	0.729561090428052	2.05928871445722	0.0013210	0.0274707	0.0246414	351
341	GO(BP): negative T cell selection	8	0.844442879264409	2.28680111922289	0.0013513	0.0277788	0.0249178	200
342	GO(BP): alpha-beta T cell lineage commitment	8	0.753441603782668	2.04036429829353	0.0013513	0.0277788	0.0249178	399
343	GO(BP): CD4-positive or CD8-positive, alpha-beta T cell lineage commitment	8	0.753441603782668	2.04036429829353	0.0013513	0.0277788	0.0249178	399
344	GO(BP): CD4-positive, alpha-beta T cell lineage commitment	8	0.753441603782668	2.04036429829353	0.0013513	0.0277788	0.0249178	399
345	GO(BP): negative thymic T cell selection	7	0.83561412673753	2.1776148491214	0.0013755	0.0282106	0.0253052	200
346	GO(BP): antigen processing and presentation of lipid antigen via MHC class Ib	5	0.907769423558897	2.08610732393129	0.0014577	0.0289024	0.0259257	189
347	GO(BP): antigen processing and presentation, endogenous lipid antigen via MHC class Ib	5	0.907769423558897	2.08610732393129	0.0014577	0.0289024	0.0259257	189
348	GO(BP): antigen processing and presentation, exogenous lipid antigen via MHC class Ib	5	0.907769423558897	2.08610732393129	0.0014577	0.0289024	0.0259257	189
349	GO(BP): natural killer cell cytokine production	6	0.843530591775326	2.07503997346434	0.0014306	0.0289024	0.0259257	318
350	GO(BP): regulation of natural killer cell cytokine production	6	0.843530591775326	2.07503997346434	0.0014306	0.0289024	0.0259257	318
351	GO(BP): regulation of gamma-delta T cell activation	6	0.826980942828485	2.03432872547128	0.0014306	0.0289024	0.0259257	351
352	GO(BP): T-helper cell lineage commitment	6	0.823943161910263	2.02685594748623	0.0014306	0.0289024	0.0259257	235
353	GO(BP): cell recognition	23	0.536188760489474	2.01220140479305	0.0022421	0.0419441	0.0376243	314
354	GO(BP): male genitalia development	4	-0.879258517034068	-2.13228361587864	0.0028901	0.0523156	0.0469275	246
355	HALLMARK_ALLOGRAFT_REJECTION	109	0.654457952477157	3.13686497053739	1e-10	2.05e-09	8.4210526	499
356	HALLMARK_INTERFERON_GAMMA_RESPONSE	103	0.539590000212048	2.56687316096843	1e-10	2.05e-09	8.4210526	616
357	HALLMARK_KRAS_SIGNALING_UP	34	0.615563899157586	2.53152112658311	1.0285931	1.4057440	5.7745582	599
358	HALLMARK_INFLAMMATORY_RESPONSE	61	0.501157707166195	2.24836876226245	8.4021416	8.6121951	3.5377438	615
359	HALLMARK_PROTEIN_SECRETION	16	-0.644153225806452	-3.26702176133847	1.3381003	1.0972422	4.5072854	723
360	HALLMARK_ADIPOGENESIS	27	-0.48429110022344	-3.15867454386599	3.7826866	2.5848358	1.0618067	888
361	HALLMARK_COMPLEMENT	45	0.524754125123553	2.25311443211423	4.7636117	2.7901154	1.1461321	657
362	HALLMARK_MYOGENESIS	20	-0.532150495316646	-2.99232620626682	1.2811928	6.5661134	2.6972481	759
363	HALLMARK_OXIDATIVE_PHOSPHORYLATION	11	-0.663147310206134	-2.72082198265307	8.4291895	0.0003839	0.0001577	682
364	HALLMARK_PEROXISOME	17	-0.502269288956127	-2.66438199234054	0.0001471	0.0006034	0.0002478	1005

365	HALLMARK_EPITHELIAL_MESENCHYMAL_TRANSITION	33	-0.344673018450739	-2.4925880029223	0.0001750	0.0006526	0.0002680	750
366	HALLMARK_ESTROGEN_RESPONSE_EARLY	33	-0.335801639683451	-2.42843243778954	0.0002670	0.0009124	0.0003748	768
367	HALLMARK_IL6_JAK_STAT3_SIGNALING	34	0.493349517789174	2.0289115862416	0.0003938	0.0012420	0.0005102	568
368	Reactome: TCR signaling	39	0.672647131742127	2.80292255329653	0.0010504	0.0092608	0.0055059	376
369	Reactome: Immunoregulatory interactions between a Lymphoid and a non-Lymphoid cell	81	0.587189012421114	2.74016500594368	0.0010121	0.0092608	0.0055059	532
370	Reactome: Generation of second messenger molecules	22	0.739908174661676	2.72304325533513	0.0011376	0.0092608	0.0055059	417
371	Reactome: Adaptive Immune System	213	0.537955900233354	2.70715454842698	0.001	0.0092608	0.0055059	580
372	Reactome: Downstream TCR signaling	28	0.678248814977203	2.62828409877528	0.0010857	0.0092608	0.0055059	488
373	Reactome: Costimulation by the CD28 family	32	0.655171239851981	2.59891116840428	0.0010787	0.0092608	0.0055059	434
374	Reactome: Immune System	446	0.492625118133771	2.57355172806112	0.0009990	0.0092608	0.0055059	636
375	Reactome: PD-1 signaling	19	0.71564872818373	2.5109196112871	0.0011574	0.0092608	0.0055059	417
376	Reactome: Phosphorylation of CD3 and TCR zeta chains	17	0.738730245112413	2.50189820854051	0.0011806	0.0092608	0.0055059	417
377	Reactome: Cytokine Signaling in Immune system	185	0.490527815889159	2.44902221823706	0.001	0.0092608	0.0055059	573
378	Reactome: Translocation of ZAP-70 to Immunological synapse	16	0.736103467998089	2.43919581688018	0.0011918	0.0092608	0.0055059	417
379	Reactome: RAC1 GTPase cycle	35	0.574858900484465	2.34011501297706	0.0010660	0.0092608	0.0055059	391
380	Reactome: Interferon Signaling	57	0.523996538528476	2.33716189482596	0.0010256	0.0092608	0.0055059	610
381	Reactome: Fc epsilon receptor (FCERI) signaling	29	0.595509093839593	2.31649444706295	0.0010869	0.0092608	0.0055059	593
382	Reactome: Hemostasis	92	0.489399289142995	2.31209478449933	0.0010070	0.0092608	0.0055059	421
383	Reactome: Cell surface interactions at the vascular wall	26	0.601668409815204	2.28953401920434	0.0011013	0.0092608	0.0055059	408
384	Reactome: Interferon gamma signaling	45	0.534689331848426	2.2860970189742	0.0010460	0.0092608	0.0055059	672
385	Reactome: Interleukin-2 family signaling	25	0.597695363362487	2.25992550089098	0.0011074	0.0092608	0.0055059	729
386	Reactome: TNFR2 non-canonical NF-kB pathway	30	0.564063201127008	2.20964500482122	0.0010822	0.0092608	0.0055059	573
387	Reactome: Constitutive Signaling by Aberrant PI3K in Cancer	15	0.66991278103234	2.19585441157902	0.0012004	0.0092608	0.0055059	423
388	Reactome: Host Interactions of HIV factors	25	0.576404971313768	2.17942512751652	0.0011074	0.0092608	0.0055059	488
389	Reactome: ER-Phagosome pathway	24	0.580802516464053	2.17940203006833	0.0011223	0.0092608	0.0055059	488
390	Reactome: CDC42 GTPase cycle	26	0.570720736272657	2.17176856860896	0.0011013	0.0092608	0.0055059	425
391	Reactome: Signaling by Interleukins	113	0.446923285153707	2.15287039059296	0.0010040	0.0092608	0.0055059	569
392	Reactome: Innate Immune System	198	0.428497467679774	2.14985920126154	0.0009990	0.0092608	0.0055059	636
393	Reactome: Chemokine receptors bind chemokines	27	0.554949704069439	2.14293565255158	0.0010893	0.0092608	0.0055059	345
394	Reactome: Antigen processing-Cross presentation	34	0.529092021238866	2.13245031027992	0.0010718	0.0092608	0.0055059	498
395	Reactome: Signaling by the B Cell Receptor (BCR)	30	0.544077418700541	2.1313532740048	0.0010822	0.0092608	0.0055059	500
396	Reactome: Class A/1 (Rhodopsin-like receptors)	56	0.471175117936394	2.0902022641121	0.0010319	0.0092608	0.0055059	900
397	Reactome: GPCR downstream signalling	98	0.439311629482937	2.08662432391228	0.0010090	0.0092608	0.0055059	609
398	Reactome: HIV Infection	34	0.509297210684273	2.05266938708581	0.0010718	0.0092608	0.0055059	502
399	Reactome: RHO GTPase cycle	69	0.443892856263626	2.02712786043181	0.0010172	0.0092608	0.0055059	425
400	Reactome: Platelet activation, signaling and aggregation	52	0.463180168633088	2.02527662323185	0.0010309	0.0092608	0.0055059	486
401	Reactome: G alpha (q) signalling events	29	0.519414396084488	2.02049066370492	0.0010869	0.0092608	0.0055059	595
402	Reactome: Signaling by GPCR	105	0.41748088159525	2.00262582550917	0.0010060	0.0092608	0.0055059	779

403	Reactome: GPVI-mediated activation cascade	13	0.703763711665854	2.22562366295748	0.00123762	0.00932535	0.0055443	351
404	Reactome: DAP12 signaling	14	0.686282684606398	2.22262781393522	0.00241545	0.01397515	0.0083088	351
405	Reactome: DAP12 interactions	18	0.624759531212061	2.14040204263718	0.00236127	0.01397515	0.0083088	492
406	Reactome: Negative regulation of the PI3K/AKT network	18	0.598882937838207	2.05174983239169	0.00236127	0.01397515	0.0083088	423
407	Reactome: PI5P, PP2A and IER3 Regulate PI3K/AKT Signaling	18	0.598882937838207	2.05174983239169	0.00236127	0.01397515	0.0083088	423
408	Reactome: Beta-catenin independent WNT signaling	22	0.553890798332614	2.03845376256516	0.0022753	0.01397515	0.0083088	306
409	Reactome: PI3K/AKT Signaling in Cancer	18	0.592960959270586	2.03146136236547	0.00236127	0.01397515	0.0083088	423
410	Reactome: Interferon alpha/beta signaling	23	0.545856285717041	2.02995062911858	0.00225479	0.01397515	0.0083088	610
411	Reactome: Parasite infection	16	0.606501690163984	2.00973973075017	0.00238379	0.01397515	0.0083088	351
412	Reactome: Leishmania phagocytosis	16	0.606501690163984	2.00973973075017	0.00238379	0.01397515	0.0083088	351
413	Reactome: FCGR3A-mediated phagocytosis	16	0.606501690163984	2.00973973075017	0.00238379	0.01397515	0.0083088	351
414	Reactome: Translation	12	-0.643360160965795	-2.78325257667321	0.00476190	0.02324580	0.01382060	722
415	Reactome: Glycosaminoglycan metabolism	12	-0.566008214810453	-2.44861885747563	0.00476190	0.02324580	0.01382060	678
416	Reactome: Metabolism of steroids	13	-0.557815695657282	-2.5703922907265	0.00515463	0.0238586	0.01418494	576
417	Reactome: COPI-mediated anterograde transport	15	-0.681767076977396	-3.43325595698382	0.00591715	0.02590750	0.01540313	543
418	Reactome: Protein localization	15	-0.593471881953643	-2.98861729001809	0.00591715	0.02590750	0.01540313	701
419	Reactome: Collagen biosynthesis and modifying enzymes	15	-0.49624451930422	-2.49899783893242	0.00591715	0.02590750	0.01540313	738
420	Reactome: COPI-dependent Golgi-to-ER retrograde traffic	18	-0.543931594949584	-2.92900047020978	0.0064516	0.02714704	0.01614005	562
421	Reactome: Golgi-to-ER retrograde transport	18	-0.543931594949584	-2.92900047020978	0.0064516	0.02714704	0.01614005	562
422	Reactome: ER to Golgi Anterograde Transport	19	-0.668349318525997	-3.63480639602318	0.00724637	0.0297193	0.01766937	677
423	Reactome: Collagen formation	19	-0.376052276274213	-2.04515390552825	0.00724637	0.0297193	0.01766937	738
424	Reactome: Transport to the Golgi and subsequent modification	22	-0.651668351870576	-3.84685666573104	0.00813008	0.03252032	0.01933469	712
425	Reactome: XBP1(S) activates chaperone genes	10	-0.541739185111768	-2.05186979732798	0.00904977	0.03409449	0.02027060	677
426	Reactome: IRE1alpha activates chaperones	10	-0.541739185111768	-2.05186979732798	0.00904977	0.03409449	0.02027060	677
427	Reactome: Post-translational protein phosphorylation	13	-0.492702566683442	-2.27035361120848	0.01030927	0.03704503	0.02202482	1022
428	Reactome: Biosynthesis of the N-glycan precursor (dolichol lipid-linked oligosaccharide, LLO) and transfer to a nascent protein	13	-0.477634225639736	-2.20091930171465	0.01030927	0.03704503	0.02202482	674
429	Reactome: Intra-Golgi and retrograde Golgi-to-ER traffic	26	-0.448623033556657	-2.83076937818189	0.01063829	0.03706245	0.02203518	562
430	Reactome: Metabolism of carbohydrates	30	-0.380804370914346	-2.56969195608817	0.0128205	0.04282315	0.02546010	694
431	Reactome: Diseases of metabolism	38	-0.27993550958653	-2.12614991074165	0.01724137	0.05122529	0.03045558	678
432	Reactome: Asparagine N-linked glycosylation	46	-0.583020161486996	-4.77431653523144	0.02173913	0.06178489	0.0367337	712
